# TOXICOLOGICAL PERSPECTIVE ON THE OSMOREGULATION AND IONOREGULATION PHYSIOLOGY OF MAJOR IONS BY FRESHWATER ANIMALS: TELEOST FISH, CRUSTACEA, AQUATIC INSECTS, AND MOLLUSCA

**DOI:** 10.1002/etc.3676

**Published:** 2016-12-30

**Authors:** Michael B. Griffith

**Affiliations:** Office of Research and Development, National Center for Environmental Assessment, US Environmental Protection Agency, Cincinnati, Ohio, USA

**Keywords:** Ionoregulation, Teleost fish, Aquatic invertebrates, Major ions, Toxicity mechanisms, Freshwater toxicity

## Abstract

Anthropogenic sources increase freshwater salinity and produce differences in constituent ions compared with natural waters. Moreover, ions differ in physiological roles and concentrations in intracellular and extracellular fluids. Four freshwater taxa groups are compared, to investigate similarities and differences in ion transport processes and what ion transport mechanisms suggest about the toxicity of these or other ions in freshwater. Although differences exist, many ion transporters are functionally similar and may belong to evolutionarily conserved protein families. For example, the Na^+^/H^+^-exchanger in teleost fish differs from the H^+^/2Na^+^ (or Ca^2+^)-exchanger in crustaceans. In osmoregulation, Na^+^ and Cl^−^ predominate. Stenohaline freshwater animals hyperregulate until they are no longer able to maintain hypertonic extracellular Na^+^ and Cl^−^ concentrations with increasing salinity and become isotonic. Toxic effects of K^+^ are related to ionoregulation and volume regulation. The ionic balance between intracellular and extracellular fluids is maintained by Na^+^/K^+^-adenosine triphosphatase (ATPase), but details are lacking on apical K^+^ transporters. Elevated H^+^ affects the maintenance of internal Na^+^ by Na^+^/H^+^ exchange; elevated HCO_3_^−^ inhibits Cl^−^ uptake. The uptake of Mg^2+^ occurs by the gills or intestine, but details are lacking on Mg^2+^ transporters. In unionid gills, SO_4_^2−^ is actively transported, but most epithelia are generally impermeant to SO_4_^2−^. Transporters of Ca^2+^ maintain homeostasis of dissolved Ca^2+^. More integration of physiology with toxicology is needed to fully understand freshwater ion effects.

## INTRODUCTION

The salinity, ionic strength, or total concentration of mineral ions in freshwater has increased in many regions from anthropogenic sources such as road salt and effluents from wastewater treatment plants (Table 1). Total ion concentrations are easily measured by testing for specific conductivity or total dissolved solids [1]. However, anthropogenic sources differ in their constituent ions (Table 1). Thus, elevated concentrations of some ions may not be the same as those in Na^+^- and Cl^−^ dominated marine waters or Ca^2+^- and HCO_3_^−^-dominated freshwaters [2]. Moreover, ions have differing physiological roles in freshwater organisms [3] and are required in different concentrations within cells.

Salinity is a primary environmental gradient differentiating freshwater, marine, or estuarine ecosystems. The evolution of the many freshwater groups, such as fish, Crustacea, and most Mollusca, involved invasion of freshwater simply by migration from at least estuarine waters [4–6]. The dual role of gills in gas exchange and ionoregulation in fish, Crustacea, and Mollusca reflect this evolution. Although some fish and Crustacea have diadromous life histories and ionoregulatory adaptations that facilitate movement among the extremes of this salinity gradient [7], many taxa have distinct lineages that are stenohaline and limited to freshwater [8]. Conversely, the evolutionary ancestors of insects and pulmonate gastropods migrated first to terrestrial environments and secondarily to freshwaters [5,9–12]. Insects adapted to terrestrial life partly by adding a lipid layer to their exoskeleton’s epicuticle to minimize water loss [13], and this layer still makes the insect cuticle relatively impermeable.

Insect groups have migrated to freshwater multiple times [9,14,15]. Some aquatic insects have evolved mechanisms to continue to breathe air, including siphons, spiracles modified to pierce the aerenchyma of aquatic plants, or ways to carry air bubbles that act as temporary physical or compressible gills. Other aquatic insects use cutaneous respiration or tracheal gills to exchange O_2_ and CO_2_ with the water [16]. Tracheal gills in aquatic insects are unlike fish or crustacean gills because O_2_ and CO_2_ diffuse across a membrane into the gas-filled tracheal system rather than to the hemolymph [17,18]. Although ionocytes or chloride cells occur on the tracheal gills or other respiratory surfaces, such as the anal organ of dragonfly nymphs, ionocytes can occur on nonrespiratory body surfaces or be clustered in specialized ion-absorption organs, such as chloride epithelia and anal papillae [19,20].

Like the aquatic insects, fully aquatic Hygrophila (Gastropoda) include species that breathe air through a diffusional lung in their mantle cavity. Others rely on cutaneous diffusion of O_2_ from the water [10]; some larger species in this group have developed neomorphic gills.

Hypo-osmotic freshwaters are very different environments in their ionoregulatory requirements from hyperosmotic marine waters [21]. Freshwaters expose all organisms inhabiting them to similar osmoregulatory and ionoregulatory requirements [21–23]. Most freshwater animals, including fish [21,23], unionid mussels [24,25], crayfish and other Crustacea [3,7], and aquatic insects [26,27], are hyperregulators that maintain greater ion concentrations in their blood or hemolymph than is found in freshwaters. Because the external medium is more dilute than body fluids, these species deal with continuous diffusional loss of salts and absorption of water across their permeable membranes. Water balance is accomplished by excretion of dilute waste fluids by their renal systems, whereas salt concentrations are maintained by various proteins on gill or renal-system membranes that actively transport ions against concentration gradients [23,24,28]. Most freshwater species, unlike saltwater species, drink little water, thereby limiting water absorption through the gastrointestinal system and dilution of the hemolymph or blood [28]. Even so, water turnover is generally greater in freshwater animals [29]. Freshwater animals reabsorb ions from their urine to produce urine that is more isotonic with the freshwater and reduce their energy expenditure for osmoregulation by 80% to 90% [30].

Taxa differ in how osmoregulation changes if the water salinity changes along a natural gradient from freshwater to saltwater. In most fish and amphibians, the ion concentrations or osmolality of intracellular (i.e., the cytosol or fluids within cells) and extracellular (i.e., the fluids outside of cells, particularly the blood or hemolymph) fluids remain relatively constant as the water osmolality increases. Most invertebrates, including unionid mussels [25] and freshwater Crustacea, are hyperregulators in freshwater and become isosmotic when placed in brackish or salt waters [3,31,32]. Hemolymph ion concentrations in freshwater molluscs are lower than in either fish or arthropods [24].

The present review assesses the current understanding of the ionoregulatory and osmoregulatory physiology of selected animal groups in freshwater ecosystems. The major ions considered include the cations H^+^, Na^+^, K^+^, Ca^2+^, and Mg^2+^ and the anions Cl^−^, HCO_3_^−^, and SO_4_^2−^. The review focuses particularly on cellular membrane transport processes that regulate the concentrations of these ions both in the cytosol of epithelial cells and in the blood or hemolymph (i.e., ionoregulation). However, the review also touches on the interaction between ionoregulation and processes that regulate movement of water into and out of these compartments (i.e., osmoregulation). A comparative approach is taken to understand how these processes may be similar or different among freshwater fish and 2 major phyla of freshwater invertebrates, the Mollusca and Arthropoda. Within the Mollusca, the focus is on bivalves in the family Unionidae, but some data on the Corbiculidae and Dreissenidae and on freshwater Gastropoda is also included. Among the Arthropoda, the focus is on the Crustacea, particularly the Decapoda and Cladocera, and aquatic Insecta. Because of their dominance among the Decapoda in inland North American freshwaters, the crayfish (Astacidea) is the primary focus, but data are also included on freshwater shrimp (Caridea) when similar studies have not been done on crayfish. The ultimate intent is to use this physiological literature to investigate 2 ecological questions. How are the transport processes for specific major ions similar or different among these freshwater animal taxa (fish, crustaceans, aquatic insects, and molluscs)? What do the transport mechanisms for each of these ions suggest about the potential of these or other ions, such as metals, Br^−^, and NO_2_^−^, to affect aquatic animal assemblages in freshwater ecosystems, when concentrations are greater than or less than concentrations normally occurring in freshwater habitats? As a shorthand, square brackets ([]) are used throughout the present review to indicate concentrations of ions or compounds.

The present review is particularly intended to benefit aquatic ecotoxicologists. An understanding of the mechanisms by which aquatic animals ionoregulate and osmoregulate is needed for ecotoxicologists to interpret the data from toxicity studies and to place the major ions, which are components of natural waters and of intracellular and extracellular fluids, into an adverse outcome pathways conceptual framework [33]. However, the review also highlights current gaps in this understanding—information that may be provided by environmental physiologists.

## GENERAL OSMOREGULATION

For freshwater animals, physiological homeostasis is attained by limiting uptake of water and maintaining higher concentrations of ions in intracellular and extracellular fluids. Although ions, such as H^+^, K^+^, and HCO_3_^−^, can contribute to the osmolality of intracellular and extracellular fluids and are also important in movement of solutes between these compartments and the external environment, Na^+^ and Cl^−^ are generally the 2 most dominant ions in terms of maintaining this hyperosmotic state in freshwater animals [21,22,34,35]. Because of their importance as primary osmolytes in intracellular and extracellular fluids, elevated Na^+^ and Cl^−^ may not have adverse effects on many freshwater animals unless the water concentrations become hyperosmotic (Supplemental Data, Table S1). Experiments with bivalves model the process whereby many invertebrates shift from hyperregulation in the low salinities characteristic of freshwaters to iso-osmolality if exposed to higher salinities, such as those characteristic of estuarine or marine waters. Whether the higher salinity causes adverse effects, such as mortality, may depend on species-specific tolerance to higher salinities and the relative concentrations of the various ions [36]. This tolerance to increased or variable salinity can be very species specific, varying among species within the same family or even genus (Supplemental Data, Table S1).

Although there is evidence for paracellular pathways, or mechanisms that allow diffusion (i.e., the random movement of molecules because of their kinetic energy causing them to intermingle and disperse from areas of higher concentrations in solutions where there are no barriers to such movement) of certain solutes through tight or septate junctions between cells [37], ionoregulation generally occurs by transport through the epithelial cells (i.e., transcellular pathways) using transporters that will be described in the following sections with water being transported along with the ions [38]. Infolding of the cell membranes helps this process by increasing the surface area across which transport occurs. Some channels across the basolateral membrane of ionocytes, such as the Cl^−^-channel and K^+^-channel (KC), are pathways out of the cell for water [39]. These channels are involved in volume regulation [40] because they transport water along with the ions across the epithelial basolateral membrane cells and into the blood or hemolymph. The water is then transported to the renal system for excretion in dilute urine. Aquaporins are another channel that increases the permeability of membranes to water; limited available data indicate that aquaporins are up-regulated in freshwater animals [41,42], where the osmoregulatory problem is water influx (Supplemental Data, Table S1).

Septate junctions can be compromised if solute concentrations in the aquatic medium exceed those of the intracellular and extracellular fluids. This may be particularly important for freshwater bivalves because these molluscs maintain lower ion concentrations in intracellular and extracellular fluids than either fish or arthropods [43] (Supplemental Data, Table S1). *Dreissena polymorpha*, a relatively recent immigrant to freshwater, appears to be particularly susceptible to hyperosmotic effects of a sugar solution because it maintains lower hemolymph osmolarity, and its septate junctions have not been tightened to minimize paracellular diffusion of solutes, a characteristic of most freshwater hyperosmoregulators [44]. One strategy that is not available to all animals is the accumulation of organic osmolytes (Supplemental Data, Table S1).

Freshwater organisms balance a combination of ion uptake with ion reabsorption that appears to minimize energy expenditure for ionic regulation. Exposure of an organism to a different salinity may increase energy expenditures as the organism attempts to adjust to this stress (Supplemental Data, Table S1). However, in *Eurytemora affinis*, an estuarine and salt marsh copepod that has independently invaded freshwater habitats multiple times in the last century, Lee et al. [45] found that freshwater populations maintain greater hemolymph osmolality than do saline populations. Such maintenance of greater hemolymph osmolality seems to require greater energy expenditure.

## ION-TRANSPORTING EPITHELIAL MEMBRANES AND ION TRANSPORT

Cell membranes contain transporter proteins that move specific solutes across the membrane [46]. Some proteins are permeases, which conserve energy by reducing the energy required for movement of ions across the membrane bilayer [47]. These proteins are similar to enzymes in that the flux of ions through the transporter follows Michaelis–Menten kinetics. Here, ion flux is related to the ion concentration by variables, *V*_max_ (i.e., maximal velocity) and *K*_m_ (i.e., Michaelis constant), which are the maximum flux rate when it is saturated by the ion concentration and the ion concentration that is half that at *V*_max_, respectively. The affinity of a transporter for an ion increases as *K*m decreases, because the transporter can transport the ion from a solution with a lower ambient ion concentration. The capacity of a transporter for an ion increases as *V*_max_ increases, because the ion flux rate increases for a solution of a particular ambient ion concentration. These proteins differ between the apical plasma membranes in contact with the external environment (or the lumen of organs that open to the external environment, such as the renal or gastrointestinal systems) and basolateral plasma membranes that interface with the extracellular fluids. Ion transfers across epithelial membranes are 2-step processes. During ion uptake, the ion is transported first across the apical membrane into the intracellular fluids and then across the basolateral membrane into extracellular fluids.

Often ion transport occurs against a gradient of electrical charge (i.e., membrane potential), solute concentration, or both (i.e., an electrochemical gradient) [47] and requires energy (i.e., active transport). Exchangers trade 2 cations or 2 anions across a membrane whereby movement of 1 ion down its electrochemical gradient supplies energy to move the other ion up its electrochemical gradient [47]. Cotransporters move electroneutral combinations of cations and anions across a membrane with movement of 1 ion along its electrochemical gradient supplying energy for transport of the other ions. Transmembrane ATPases use energy supplied by hydrolysis of adenosine triphosphate to transport either single ions or exchange different ions. The energy demand is enough that glycogen-rich cells containing glycogen phosphorylase surround ionocytes in tilapia (*Oreochromis mossambicus*) gills [48]. Fish energy expenditure for ionoregulation and osmoregulation increases as environmental salinity varies from isotonicity, and more energy is required for up-regulation of ion transporters [49,50]. In cutthroat trout (*Oncorhynchus clarkii*), O_2_ consumption decreased in isolated gills treated with 0.5 mM ouabain to inhibit Na^+^/K^+^-adenosine triphosphatase (ATPase; NKA) or 1 μM bafilomycin A_1_ to inhibit vacuolar-type H^+^-ATPase (VHA) [51]. Approximately 1.8% of resting metabolic rate was used for Na^+^ transport by these 2 transporters. Also, O_2_ consumption of cutthroat trout gills in freshwater was slightly greater than that in saltwater (3.9% vs 2.4% of resting metabolic rate, respectively).

Conversely, channels transport a single ion across a membrane, usually down its electrochemical gradient. This is a type of passive transport [47]. Because of their ion specificity, channels often create a difference in electrical potential across an epithelial membrane.

Although ion transporters are central to maintenance of hypertonic extracellular ion concentrations in freshwater, mechanisms that regulate ion loss from diffusive leakage across the gill epithelia are also important. Most aquatic animal epithelia, despite their permeability to water, gases, and some ions, are resistant (or tight) to penetration by nonelectrolytes [37], and this tightness allows these animals to hyperregulate in freshwater. This tightness involves maintaining septate junctions in invertebrates and tight junctions in vertebrates [52,53]. These intercellular junctions, although not homologous [54], are barriers to solute diffusion through the spaces between adjoining epithelial cells.

The tight junctions between fish gill epithelial cells are characterized by a cross-linked complex of multiple proteins, including transmembrane proteins, such as claudins and occludins, along with zona occludens and cingulin, plaque proteins that anchor the transmembrane proteins to the actin cytoskeleton [55]. Claudins and occludin are integral components of tight junctions between fish gill epithelial cells (Supplemental Data, Table S2).

Cells involved in transport have limited distributions on the epithelia of most organisms. Whereas cells in the gastrointestinal system absorb nutrients and energy from food and those in the renal system remove waste products and retain ions, the gills are a particularly important interface for ionoregulation. Because gill membranes are involved in gas exchange with the external environment, they are by necessity very thin. The membranes are also in direct contact with the surrounding water, which is a source of ions for aquatic organisms. In larval fish and some aquatic insects that lack gills, other epithelial membranes on the body surface are involved in ion transport.

## TRANSPORTERS AND PHYSIOLOGY OF INDIVIDUAL IONS

### Sodium (Na^+^)

The primary cation involved in osmoregulation in animals is Na^+^. Uptake of Na^+^ across the apical plasma membrane is accomplished by exchange with H^+^, a product of metabolism [56]. Research with fish and other freshwater organisms supports a model in which Na^+^ is exchanged for H^+^ across the apical membrane by 1 or both of 2 molecular systems [57,58]. Because Na^+^ is exchanged for H^+^, Na^+^ also is involved in acid-base regulation in these animals [15,59–61].

#### Teleost fish.

**Figure 1.**
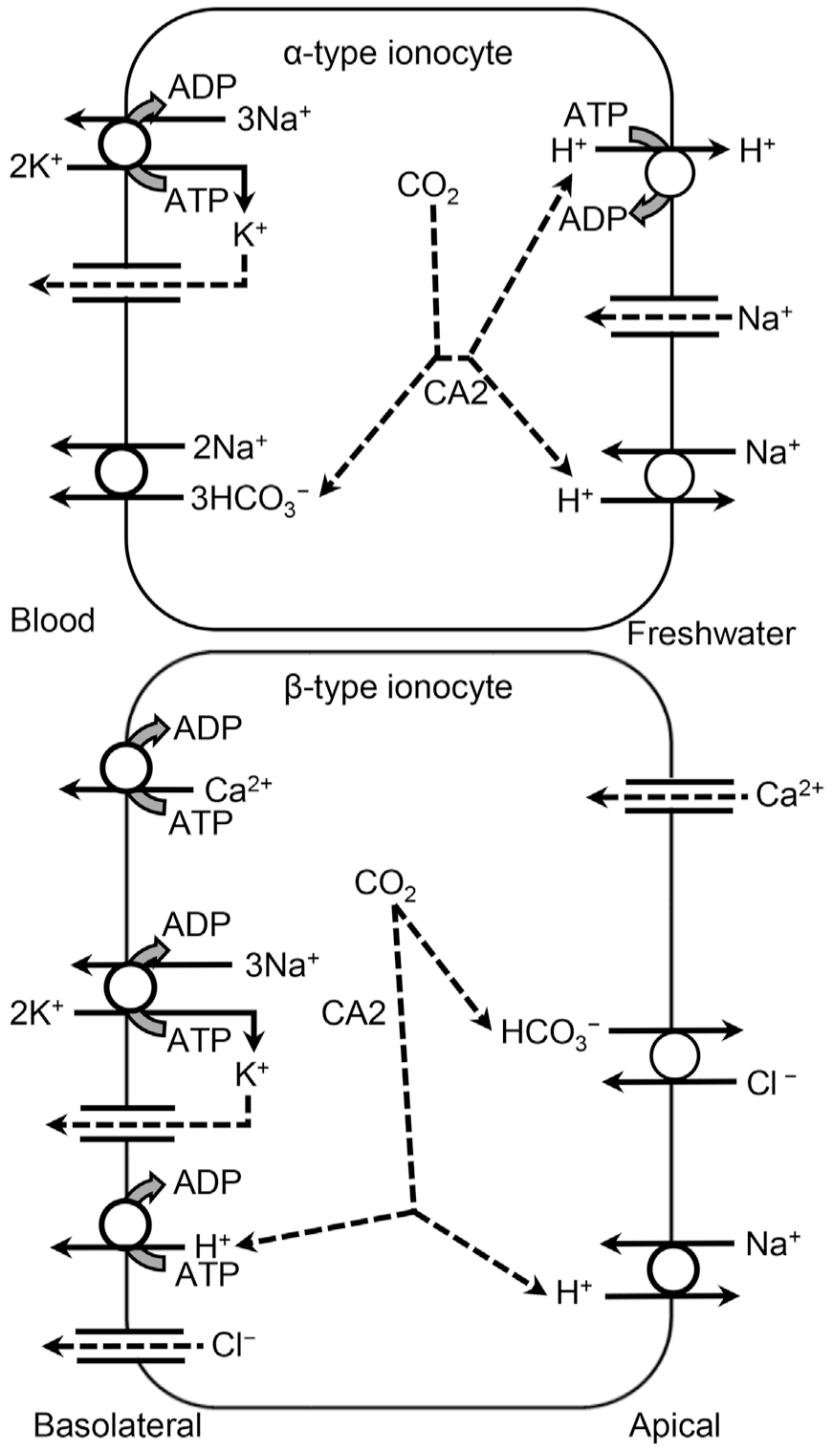
Current model for transporters on α-type and β-type ionocytes of fish such as salmonids. In the α-type ionocyte, along the apical membrane are vacuolar-type H^+^-adenosine triphosphatase (ATPase; VHA), apical Na^+^-channel, and Na^+^/H^+^-exchanger (NHE), whereas along the basolateral membrane are Na^+^/K^+^-ATPase (NKA), K^+^-channel (KC), and Na^+^/HCO_3_^−^-cotransporter (NBC). In the β-type ionocyte, along the apical membrane are the epithelial Ca^2+^-channel (ECaC), anion exchanger (AE), and NHE, whereas along the basolateral membrane are plasma membrane Ca^2+^-ATPase (PMCA), NKA, KC, VHA, and Cl^−^-channel. The carbonic anhydrase type 2 enzyme is CA2. Dashed arrows indicate diffusion, whereas solid arrows indicate active transport. Arrows that split indicate reactions. Modified from Dymowska et al. [303]. ATP=adenosine triphosphate; ADP=adenosine diphosphate.

**Figure 2.**
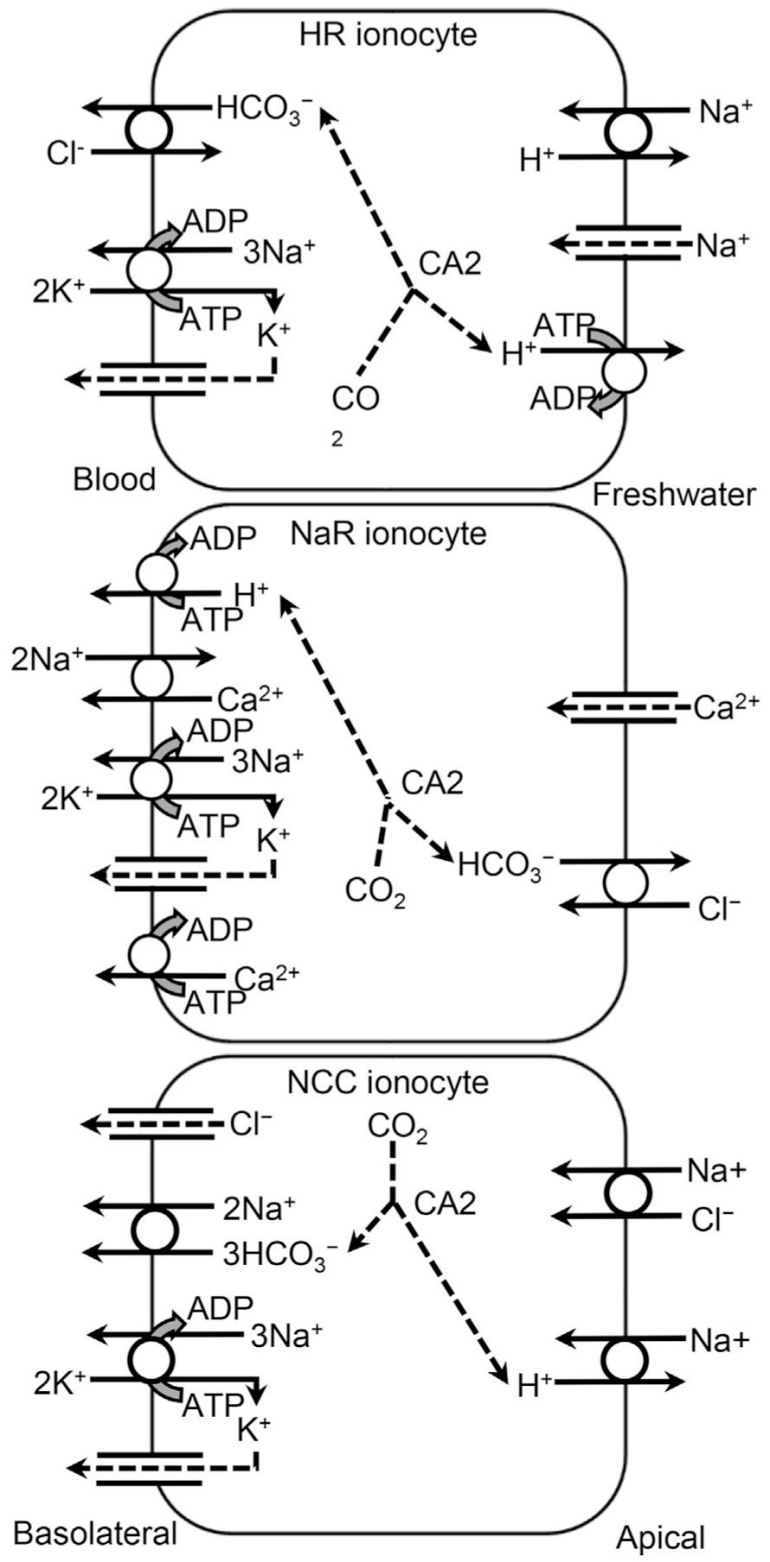
Current model for transporters on H^+^-adenosine triphosphatase (ATPase)-rich (HR), Na^+^/K^+^-ATPase-rich (NaR), and Na^+^/Cl^−^-cotransporter (NCC) ionocytes of fish, such as zebrafish. In the HR ionocyte along the apical membrane are Na^+^/H^+^-exchanger (NHE), apical Na^+^-channel, and vacuolar-type H^+^-ATPase (VHA), whereas along the basolateral membrane are the anion exchanger (AE), Na^+^/K^+^-ATPase (NKA) and K^+^-channel (KC). In the NaR ionocyte, along the apical membrane are the epithelial Ca^2+^-channel (ECaC) and anion exchanger, whereas along the basolateral membrane are VHA, Na^+^/Ca^2+^-exchanger (NCX), NKA, KC, and plasma membrane Ca^2+^-ATPase (PMCA). In the NCC ionocyte, along the apical membrane are NCC and NHE, whereas along the basolateral membrane are the Cl^−^-channel, Na^+^/HCO_3_^−^-cotransporter (NBC), NKA, and KC. Dashed arrows indicate diffusion, whereas solid arrows indicate active transport. Arrows that split indicate reactions. Modified from Dymowska et al. [303]. CA2=carbonic anhydrase type 2 enzyme; ATP=adenosine triphosphate; ADP=adenosine diphosphate.

In fish, an electroneutral Na^+^/H^+^-exchanger (NHE) transports 1H^+^ across the apical membrane in exchange for 1Na^+^ [62,63]. The second system involves 2 transporters: 1) VHA transports H^+^ out across the apical membrane [64,65], creating an electrochemical gradient for 2) Na^+^ to diffuse across the apical membrane through an amiloride-sensitive apical Na^+^-channel that may also be permeable to K^+^ [23,66,67]. Molecular research has been unable to identify messenger ribonucleic acid (mRNA) or proteins associated with an epithelial Na^+^-channel (ENaC) as described in mammals [68] in gill ionocytes of tilapia or zebrafish (*Danio rerio*) [69–71]. However, a related acidsensing ion channel (ASIC), specifically ASIC 4, was recently identified on the ionocyte apical membranes of rainbow trout (*Oncorhynchus mykiss*) and zebrafish that appears to act as an Na^+^-channel [72,73]. Parks et al. [74] questioned whether both types of molecular systems could function in freshwater, but their subsequent research has identified both systems in freshwater fish [75,76] (Supplemental Data, Table S3). Ionocytes have morphological characteristics that create localized microenvironments with low [Na^+^] or higher [H^+^] that facilitate operation of NHE [63] (Supplemental Data, Table S3). Carbonic anhydrase (CA) plays a role in Na^+^ transport by catalyzing production of H^+^ from the hydrolysis of CO_2_ [77] (Figures 1 and 2 and Table 2).

Uptake of Na^+^ from food occurs in the gastrointestinal system of freshwater fish. However, the relative importance of this source of Na^+^ is not clear (Supplemental Data, Table S3).

The relative concentrations of Na^+^ and K^+^ differ between the intracellular and extracellular fluids of animal cells [47]: intracellular fluids contain greater [K^+^] compared with [Na^+^] whereas the reverse occurs in extracellular fluids. The difference can be as much as 10-fold. These opposing concentration gradients are maintained by active transport involving NKA on the basolateral membrane [66] (Supplemental Data, Table S3).

Fish gills have important roles in gas exchange, ionoregulation, and acid–base regulation [61]. The 2 apical Na^+^ transport systems have different roles in these functions and are accordingly segregated among different ionocyte types (Figures 1 and 2 and Supplemental Data, Table S3). An NHE isoform, NHE3b, is expressed predominantly in the gills and is relevant to Na^+^ uptake under low Na^+^ or acidic conditions. Particularly under acidic conditions at least in larval zebrafish, zNHE3b appears to be tied to a metabolon that includes excretion of NH_3_ by the Rhesus protein Rhcg1 [78] (Supplemental Data and Supplemental Data, Table S3).

#### Crustacea.

**Figure 3.**
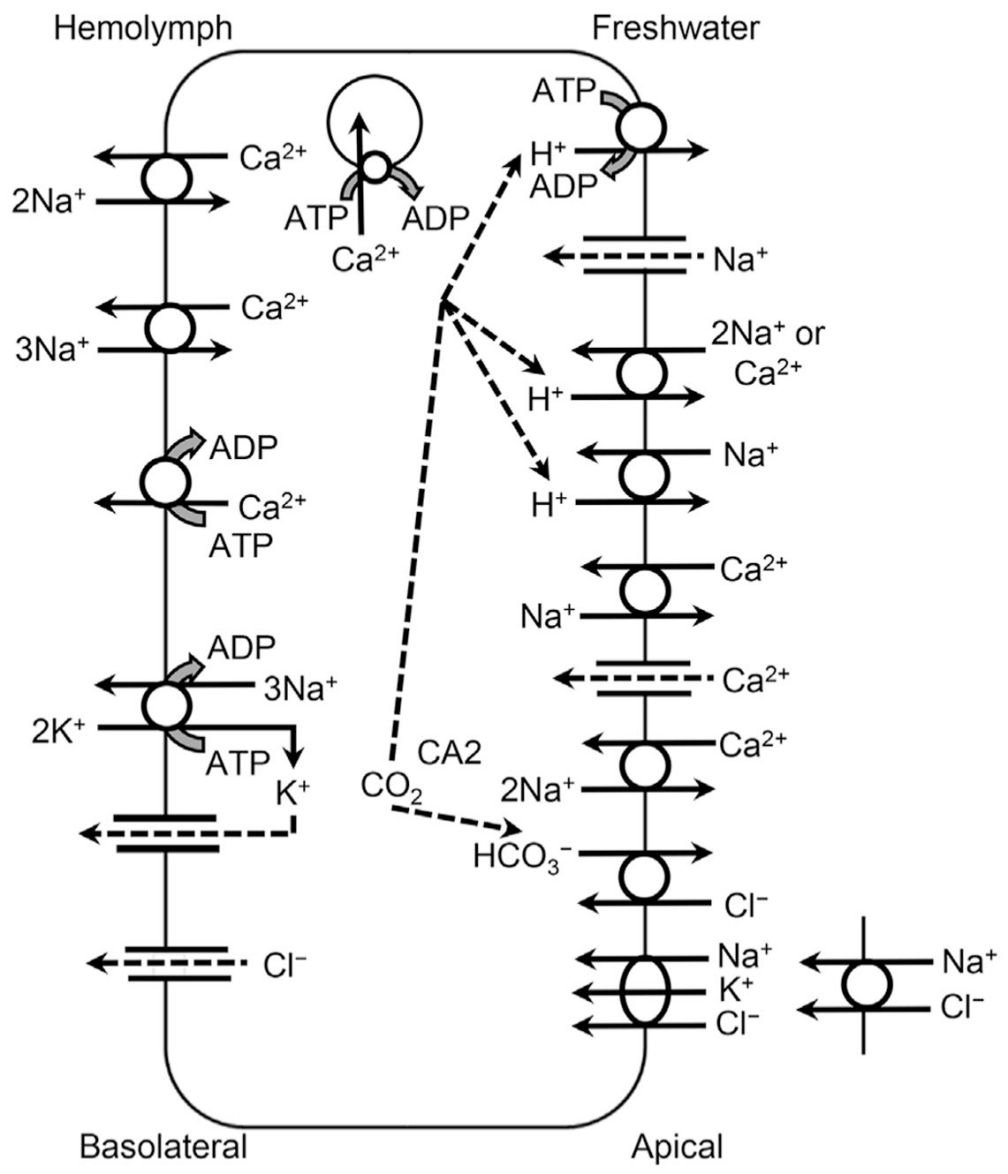
Generalized model for transporters on gill ionocytes of freshwater Crustacea based on transporters identified by the present review. A single cell is shown because no studies have identified different ionocyte types. Along the apical membrane are vacuolar-type H^+^-adenosine triphosphatase (ATPase; VHA), apical Na^+^-channel, electrogenic H^+^/2Na^+^ (or Ca^2+^)-exchanger [H2Na(Ca)E], electroneutral Na^+^/H^+^-exchanger (NHE), Na^+^/Ca^2+^-exchanger (NCX), epithelial Ca^2+^-channel (ECaC), electroneutral NCX (2Na^+^/Ca^2+^), anion exchanger, and Na^+^/K^+^/Cl^−^-cotransporter (NKCC; *Daphnia magna* adults) or Na^+^/Cl^−^-cotransporter (NCC; *D*. *magna* neonates). On the basolateral membrane are the electroneutral NCX (2Na^+^/Ca^2+^), electrogenic NCX (3Na^+^/Ca^2+^), plasma membrane Ca^2+^-ATPase (PMCA), Na^+^/K^+^-ATPase (NKA), K^+^-channel (KC), and Cl^−^-channel. Plasma membrane Ca^2+^-ATPase may also move Ca^2+^ into organelles within the cells. Dashed arrows indicate diffusion, whereas solid arrows indicate active transport. Arrows that split indicate reactions. CA2=carbonic anhydrase type 2 enzyme; ATP=adenosine triphosphate; ADP=adenosine diphosphate.

Similar ion transporters (Figure 3 and Table 2) that exchange H^+^ for Na^+^ exist in crayfish [67,79], but while the paired VHA and apical Na^+^-channel are potentially homologous to those in fish, Crustacea possess an electrogenic H^+^/2Na^+^- (or Ca^2+^) exchanger [H2Na(Ca)E] [80–83] on gill epithelia apical membranes [84]. This exchanger also transports Ca^2+^, and elevated external [Ca^2+^] can competitively inhibit Na^+^ uptake [80,81,85]. However, an electroneutral, amiloride-sensitive NHE was identified on the gill epithelium apical membrane of *Orconectes limosus* opposite from NKA on the basolateral membrane [86]. As in fish, Na^+^ movement across the apical membrane of crayfish is facilitated by maintenance of intracellular [Na^+^] that are lower than hemolymph [Na^+^] by Na^+^ transport across the basolateral membrane by NKA.

#### Aquatic insects.

In freshwater mosquito (Culicidae) or midge (Chironomidae) larvae, 4 anal papillae on abdominal segment 10 are the primary sites of Cl^−^ and Na^+^ absorption [87–89]. Vacuolar-type H^+^-ATPase is found on the apical membrane of anal papillae epithelial ionocytes, whereas P-type NKA is found on the basolateral membrane [88] (Supplemental Data, Table S3).

**Figure 4.**
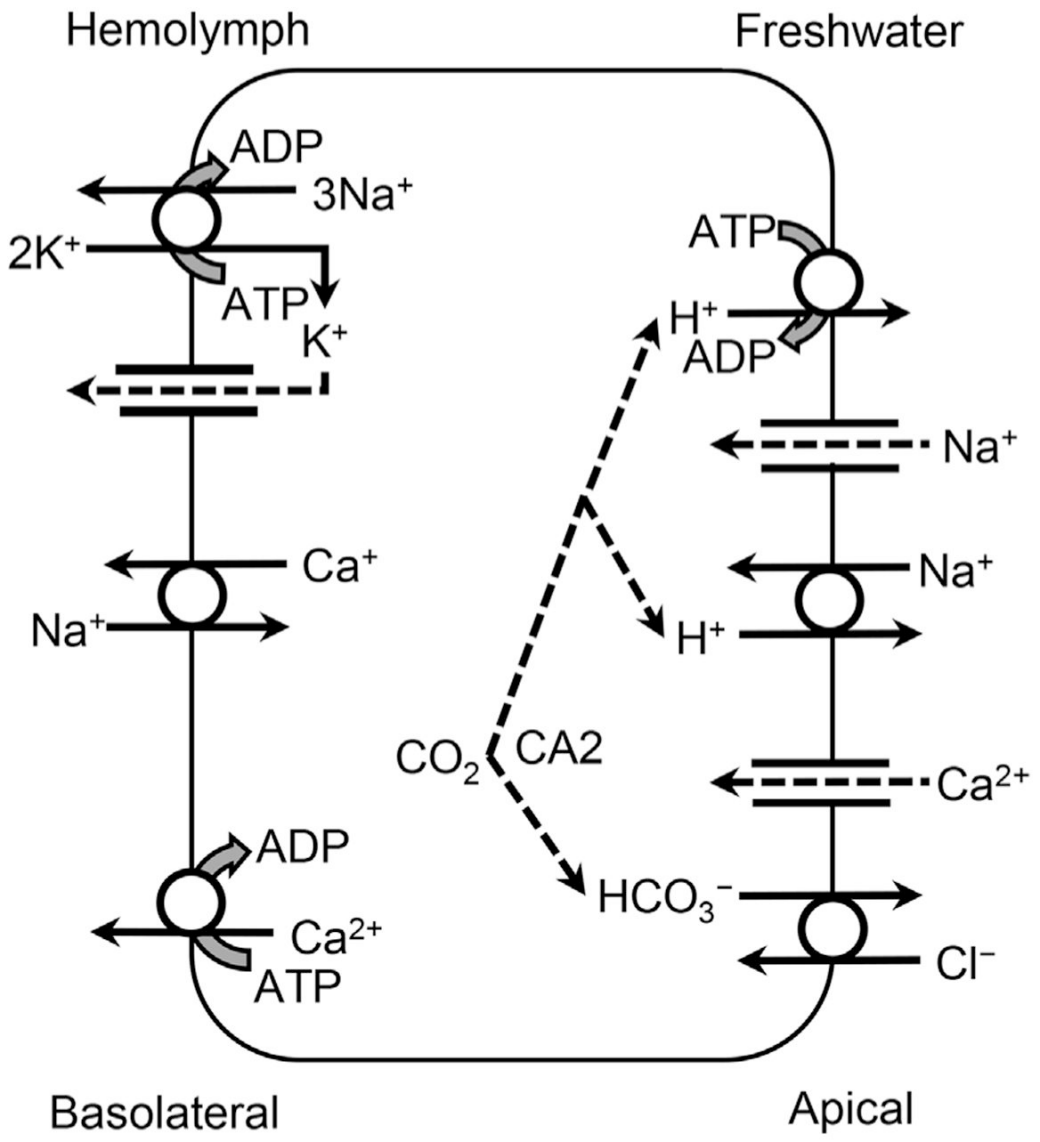
Generalized model for transporters on epithelial ionocytes of aquatic insects based on transporters identified by the present review. A single cell is shown because no studies have identified different ionocyte types, although research with mosquito larvae suggests that transporters for at least K^+^ and Ca^2+^ are not collocated with those for Na^+^ and Cl^−^ on the anal papillae. Also, the evidence suggests variation among aquatic insect orders. Along the apical membrane are vacuolar-type H^+^-adenosine triphosphatase (ATPase; VHA), apical Na^+^-channel, Na^+^/H^+^-exchanger (NHE), epithelial Ca^2+^-channel (ECaC), and anion exchanger (AE). On the basolateral membrane are Na^+^/K^+^-ATPase (NKA). K^+^-channel (KC), Na^+^/Ca^2+^-exchanger (NCX), and plasma membrane Ca^2+^-ATPase (PMCA). Dashed arrows indicate diffusion, whereas solid arrows indicate active transport. Arrows that split indicate reactions. CA2=carbonic anhydrase type 2 enzyme; ATP=adenosine triphosphate; ADP=adenosine diphosphate.

Export of H^+^ across the apical membrane creates a negative potential that favors counter, electrodiffusive movement of Na^+^ possibly through an apical Na^+^-channel [90] with NKA maintaining a lower concentration gradient across the apical membrane by transporting Na^+^ across the basolateral membrane (Figure 4 and Table 2). Also present is NHE, but it differs at least pharmacologically from mammalian NHE [91] (Supplemental Data, Table S3).

In early experiments to elucidate interactions between different anions and cations and Na^+^ influx in *Chironomus tentans*, Wright [92] observed inhibition of Na^+^ uptake by elevated [NO_3_^−^], [SO_4_^2−^], [Li^+^], [Rb^+^], [NH_4_^+^], or [H^+^] in the external medium. Based on current models of Na^+^ transport, elevated [NH_4_^+^] or [H^+^] affect the counter export of H^+^ by VHA required for Na^+^ influx by an apical Na^+^-channel. Substitution of Li^+^ for Na^+^ in the apical Na^+^-channel [93] appears to result in competitive inhibition of Na^+^ uptake, whereas Rb^+^ substitution for K^+^ may affect NKA, which maintains the concentration gradients of Na^+^ across both the apical and basolateral membranes. The effects of NO_3_^−^ and SO_4_^2−^ are not readily explained, but Wright [94] suggested that Na^+^ transport between the water and hemolymph is coupled with independent Cl^−^-transport, such that a relatively impermeant anion, such as SO_4_^2−^ or NO_3_^−^, cannot substitute for Cl^−^. Data on Na^+^-transport in other aquatic insect classes are more limited (Supplemental Data, Table S3).

#### Mollusca.

**Figure 5.**
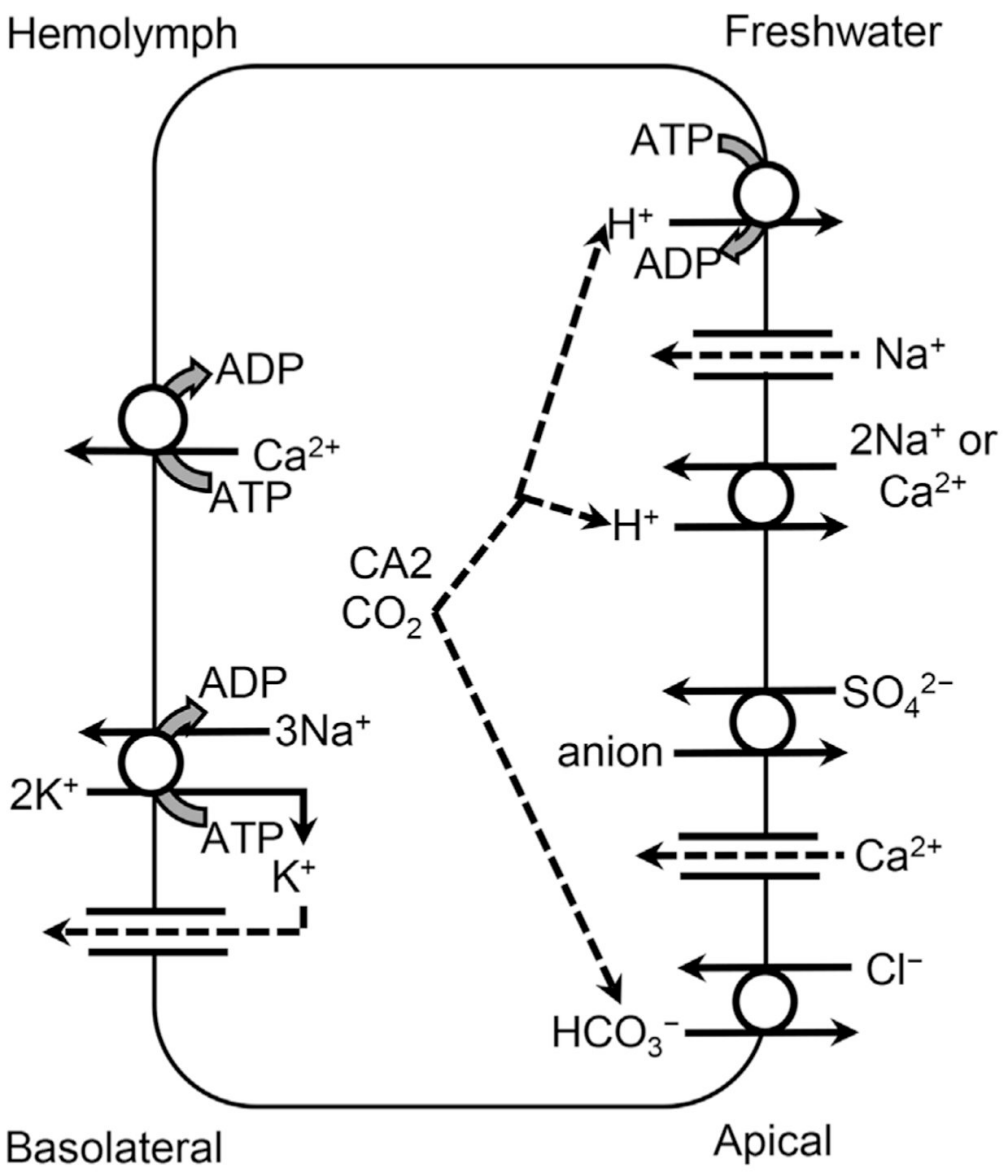
Generalized model for transporters on gill ionocytes of Unionidae (Mollusca) based on transporters identified by the present review. A single cell is shown because no studies have identified different ionocyte types. Along the apical membrane are vacuolar-type H^+^-adenosine triphosphatase (ATPase; VHA), apical Na^+^-channel, electrogenic H^+^/2Na^+^ (or Ca^2+^)-exchanger [H2Na(Ca)E], anion/SO_4_^2−^-exchanger, epithelial Ca^2+^-channel (ECaC), and anion exchanger. On the basolateral membrane are plasma membrane Ca^2+^-ATPase (PMCA), Na^+^/K^+^-ATPase (NKA), and K^+^-channel (KC). Dashed arrows indicate diffusion, whereas solid arrows indicate active transport. Arrows that split indicate reactions. CA2=carbonic anhydrase type 2 enzyme; ATP=adenosine triphosphate; ADP=adenosine diphosphate.

In freshwater bivalves, Na^+^ uptake across the apical epithelial membrane occurs by exchange for H+ [35,95] and at least includes an NHE (Figure 5 and Table 2). In unionid mussels, maintenance of ionic homeostasis relies more on active uptake of Na^+^ than uptake of Cl^−^. However, whether the NHE is electroneutral, as in fish, or electrogenic, as in Crustacea, is unknown. In freshwater, NKA activity is greater in the unionid gills than in the gills of oligohaline bivalves [96], suggesting that this transporter plays a role in maintaining a [Na^+^] gradient across the epithelial membrane [95,97], as in fish.

#### Other Na^+^ transporters.

A Na^+^/HCO_3_^−^-cotransporter (NBC) on the basolateral membrane of gill epithelia helps maintain very low [Na^+^] in the cytosol and also removes HCO_3_^−^ from cells that primarily export H^+^ in some fish [21,69,70,98,99]. The NBC occurs in rainbow trout, zebrafish, and Osorezan dace (*Tribolodon hakonensis*) gill ionocytes [69,99] but not in American eel (*Anguilla rostrata*) [100].

In some fish, there is a transporter for both Na^+^ and Cl^−^ on the apical membrane of specific epithelial cell types. This electroneutral Na^+^/Cl^−^-cotransporter (NCC) has been detected in the apical membrane of type II ionocytes of tilapia embryos reared in freshwater [69] and in NCC ionocytes of zebrafish (Figure 2 and Table 2) [70,99,101].

In neonates of the crustacean *Daphnia magna*, an NCC has been detected by inhibition of Na^+^-uptake with either bumetanide (NCC and Na^+^/K^+^/Cl^−^-cotransporter [NKCC] inhibitor) and thiazide (NCC inhibitor) [102]. There is an ontogenic change between the neonates and adults, because NKCC was detected in adult *D. magna* by the inhibition of Na^+^-uptake by bumetanide and not by thiazide. The function of NKCC in these cladoceran adults (i.e., ion uptake) differs from that in fish, where an NKCC is active in fish from more saline habitats and is involved in ion excretion [69,103–105].

#### Effects on Na^+^ transport by other ions in the water.

Low pH inhibits influx of Na^+^ by increasing the [H^+^] gradient against which the NHE or VHA act [106–108]. Low pH also increases gill epithelial ion permeability, resulting in increased Na^+^ efflux [109–112]. The result is a large decrease in blood [Na^+^] in fish, although sensitivity can vary significantly among species. Similar loss of Na^+^ has been observed in crayfish, various classes of aquatic insects, and unionid mussels (Supplemental Data, Table S3).

Elevated Ag^+^ and Cu^2+^ are also associated with reductions in blood [Na^+^] resulting from decreased Na^+^ in fish [113]. Research on metal uptake indicates that the ENaC is involved in the uptake of Cu^2+^ and Ag^+^ by fish, particularly in low [Na^+^] freshwaters. Interactions between Na^+^ and these 2 metals suggest noncompetitive inhibition at the apical Na^+^-channel, through which these ions all can be transported [114,115] (Supplemental Data, Table S3). Copper is transported by the apical Na^+^-channel, because the ion crosses as Cu^+^ rather than Cu^2+^ [116,117]. Reduced Na^+^ uptake is because of inhibition of NKA by Cu^2+^ or Ag^+^ [118]. Also, acute Ag^+^ exposure inhibits CA activity, which limits the availability of H^+^ for exchange with Na^+^. This is also observed in freshwater Crustacea and Mollusca (Supplemental Data, Table S3), although Cu^2+^ may also be transported through an electrogenic H2Na(Ca) E [119–121]. Other divalent metals are known to reduce Na^+^ uptake by inhibiting basolateral NKA or CA activity (Supplemental Data, Table S3). *Chironomus* spp. have demonstrated a similar relationship between whole-body [Na^+^] and NKA inhibition by metals (Supplemental Data, Table S3), but Na^+^ uptake by *Maccaffertium pudicum* (Ephemeroptera), several Plecoptera, and another dipteran (*Atherix* sp.) showed variable effects with exposure to Ag^+^ or Cu^2+^ [122], suggesting some differences in the Na^+^ transporters between these insects and other freshwater animals.

#### Synthesis.

Across both freshwater invertebrate and fish species, evidence exists for exchange of Na^+^ for H^+^ on the apical membrane of ionocytes either on gill or other epithelia involved in ion exchange between the organisms and water. There is also significant evidence that increasing [H^+^] in the water inhibits Na^+^ uptake, either by competition for external attachment sites on the NHE between these 2 cations [123] or by increasing the concentration gradient for export of H^+^. However, there is much variability; 1 factor affecting species-specific sensitivity to acidification may be the epithelial membrane’s permeability to Na^+^ loss under low pH conditions [124]. This leads to 1 hypothesis that the reason Ephemeroptera, in particular, exhibit sensitivity to acidification, metals, and other ionoregulatory challenges is because of the high permeability of their larval integument. Unlike other aquatic insects, the ionocytes of Ephemeroptera are scattered as single cells or cell complexes not just located on the tracheal gills but also over the rest of the body integument [125]. Moreover, each ionocyte is separated from the external medium by only a thin porous plate (≈0.1 mm). Therefore, epithelial membrane permeability might be greater than in other aquatic insects.

Among the species that have been studied, VHA and NKA are functionally very similar and may be biochemically similar enough to be classified in the same molecular families [126,127]. However, there has been minimal research with the aquatic insects, which because of their evolutionary history, could be the most divergent. In the case of the NHE, at least the Crustacea have evolved an electrogenic transporter that transports either Na^+^ or Ca^2+^ owing to these animals’ requirements for Ca^2+^ in their exoskeleton. However, there is uncertainty here because newer molecular methods used to study vertebrates have not yet been applied to invertebrates.

There have been no extensive comparisons even within phyla, but there is evidence that the affinity and capacity of these transporters as measured by *K*_m_ and *V*_max_ can be variable among species and even populations within a species. This can result in significant intraspecific variance in the tolerance of animals to low [Na^+^].

In research that tried to assess the relative toxicity of various major ions for *Ceriodaphnia dubia, D. magna*, and fathead minnows (*Pimephales promelas*), Na^+^ was not a significant variable in the resulting regressions [128]. A number of recent studies of the relationship between hardness and acute effects of Na^+^ salts have found toxicity for a range of freshwater fish, crustaceans, aquatic insects, and molluscs at relatively high concentrations: 5.3 mM to 147.1 mM for Na_2_SO_4_ [129–133] and 3.7 mM to 170.1 mM for NaCl [134,135]. As the primary osmoregulatory cation in animals, Na^+^ is regulated by the apical and basolateral membrane transporters and maintained at levels in the extracellular and intracellular fluids that exceed ambient [Na^+^] of most natural freshwaters. In diadromous species that migrate between freshwater and brackish or saltwater as part of their life cycle, the arrangement and molecular isoforms of these transporters often change, in part to shift from hyper-regulation and uptake of Na^+^ in hypotonic freshwaters to hyporegulation or isoregulation and excretion of Na^+^ in hypertonic estuarine or marine waters [136–138]. Many stenohaline freshwater species are unable to make such changes and tolerate [Na^+^] that is hypoionic but begin to exhibit adverse effects as the ambient [Na^+^] approaches or exceeds that of intracellular fluids and blood or hemolymph [139–144] and the directions of water movement and ion diffusion change.

### Potassium (K+)

Potassium is important in Na^+^ transport from the cytosol across the basolateral membrane to extracellular fluids by NKA. Furthermore, the greater cytosol [K^+^] allows diffusion of K^+^ back across the basolateral membrane to the extracellular fluids through a KC. The primary transporter for Na^+^ across the basolateral membrane is Na^+^/K^+^-ATPase [21,88].

The KC, which moves K^+^ back across the basolateral membrane to the extracellular fluids, is structured so that it is also somewhat permeable to larger alkali metal ions, such as Rb^+^ and Cs^+^, but relatively impermeable to Na^+^ and Li^+^ [145]. This channel limits its permeability to K^+^ but also positions ions attached to the transporter so that the repulsive force between pairs of K^+^ ions enhances their conduction through the transporter [145].

#### Teleost fish.

The flux of K^+^ across the apical membrane of gill epithelia in freshwater fish, such as rainbow trout, occurs by active transport against a concentration gradient [146], which is maintained, in part, by the exchange of Na^+^ for K^+^ by NKA across the basolateral membrane (Figure 1 and Table 2). This has been shown in a few studies that have used either ^42^K as a radiotracer or ^86^Rb as an analog for K, although Rb^+^ penetrated fish tissues much less than K^+^ [146]. Some studies have suggested that the amiloride-sensitive apical Na^+^-channel may also be permeable to K^+^ [23,66,67,147], but no research has conclusively identified an apical transporter involved in K^+^ uptake.

In zebrafish embryos, complementary deoxyribonucleic acid was identified as *kcnj1*, an ortholog of *Kir1.1b* that encodes the mammalian kidney K^+^-channel (ROMK) [148]. Although expression of *kcnj1* occurred in the duct midsegment of the pronephros, the gene was also expressed in the ventrolateral integument and in a cell population in the gill primordia that also expressed the *a1a.4* subunit isoform of NKA. Abbas et al. [148] suggested that this cell population is a fourth ionocyte type in which the encoded protein, kcnj1, is located on the apical membrane and is involved in K^+^ excretion, not uptake. Similarly, a ROMK is expressed in gill ionocytes of saltwater-acclimated tilapia, where it is also involved in K^+^ excretion [149]. However, bands for ROMK were not detected by Western blot analysis in the gills of freshwater-acclimated tilapia, suggesting that this transporter is not active in freshwater. Because this evidence is equivocal for freshwater fish, this potential transporter or the potential ionocyte is not illustrated in Figure 2. Uptake of K^+^ also occurs from food in the gastrointestinal tract (Supplemental Data, Table S4).

#### Crustacea.

As with Na^+^, uptake of K^+^ increases in crayfish during the postmolt period to counteract the dilution of ion hemolymph concentrations that result from absorption of water to increase body volume following ecdysis [150,151]. However, no research has identified a mechanism for apical K^+^ uptake.

An NKCC has been detected in adult *D. magna* [102]. This transporter is involved in Na^+^ and presumably K^+^ uptake.

#### Aquatic insects.

Although influx of Na^+^ and Cl^−^ and efflux of H^+^ from the anal papillae of *Chironomus riparius* demonstrate that the papillae are a site of exchange for these ions, K^+^ fluxes are near 0 [89,152], suggesting that the anal papillae are not the location of K^+^ uptake. Conversely, when the anal papillae of *Aedes aegypti* larvae were removed there was less uptake of K^+^ when the larvae were placed in 1.7 mM KCl for 12 h to 14 h [153].

Pullikuth et al. [154] identified a K^+^/2H^+^-exchanger, possibly energized by VHA [155], in the midgut of mosquito larvae, and maximum absorption of K^+^ occurred in the midgut of the midge *C. riparius* [152]. A hypothesized relationship between the K^+^/2H^+^-exchanger and VHA was tested by inhibiting VHA with amiloride or increasing the luminal [K^+^], but neither decreased the alkalization, suggesting that these 2 transporters are independent [156]. A similar K^+^/2H^+^-exchanger has been studied extensively in terrestrial larvae of *Manduca sexta* (Supplemental Data, Table S4).

The whole-animal [Na]:[K] ratio in *A. aegypti* larvae was approximately 2.5:1, whereas the ratio in the hemolymph was 25:1 [153]. Moreover, larvae survived for 3 wk or more in distilled water, suggesting that the tissues held a reserve of K^+^ for maintaining the low hemolymph concentrations. Other information on K^+^-transport in aquatic insects is limited (Supplemental Data, Table S4).

#### Mollusca.

Freshwater mussels require a minimal [K^+^] but also do not tolerate high concentrations [157,158]. Optimal [K^+^] in artificial freshwater for *D. polymorpha* ranged from 0.5 mM to 1.0 mM, and survival times decreased when [K^+^] exceeded 1.5 mM [36,159]. Peak survival of *D. polymorpha* was observed when the [K^+^]:[Na^+^] ratio ranged from 0.01 to 0.02, and this bivalve tolerated artificial saltwater with a [K^+^]:[Na^+^] ratio in this range [36]. A similar [K^+^]: [Na^+^] ratio in the blood is found for Mollusca from various salinities [160]. The requirement for K^+^ is related to its role in internal ion transport (Supplemental Data, Table S4), particularly the transport of Na^+^ across the basolateral membrane via the NKA (Figure 5 and Table 2) [95]. Potassium also plays a role in cell volume regulation (Supplemental Data, Table S4).

#### Effects on K^+^ transport by other ions in the water.

In trout exposed to water with some combination of low pH, low Ca^2+^, and Al, either whole-body [K^+^] decreased or net efflux of K^+^ increased. Similar increases in efflux or decreases in hemolymph [K^+^] have been observed in crayfish exposed to low pH (Supplemental Data, Table S4). If K^+^ transport across the apical epithelial membrane is by exchange with H^+^, as has been suggested for some fish [23,147], inhibition of this transport may explain these observations, although increased efflux through more permeable epithelial tight junctions are also likely involved.

#### Synthesis.

K^+^ is a dominant intracellular cation and plays a well-studied role in transporting Na^+^ across the basolateral membrane between the intracellular fluids and hemolymph or blood by NKA. Nevertheless, few details are available on how freshwater organisms obtain K^+^ from the external environment, other than a few conjectured pathways with little evidence that NHE may transport K^+^ and the presence of NKCC in adult *Daphnia*. Of the major ions, K^+^ generally has the lowest ambient concentrations in freshwaters [161], so the concentration gradient across the gill epithelial apical membrane may be relatively steep. For example, hemolymph [K^+^] in *D. polymorpha* was 0.4 ± 0.0 mM K+ compared with 0.05 mM K^+^ in pond water [60], and intracellular [K^+^] is expected to be greater. There is evidence for active transport of K^+^ from ambient freshwater for fish, crustaceans, and molluscs. The data for active uptake, at least through the anal papillae of freshwater Diptera, is mixed. However, there may be some evidence for uptake through the gastrointestinal system in aquatic insects.

Molluscs are sensitive to elevated water [K^+^] to the point that K^+^ is used as a molluscicide for *D. polymorpha* in water intake structures [159,162]. The most toxic of the major ions for *C. dubia, D. magna*, and fathead minnow was K^+^ [128]. The available research with Mollusca suggests that these effects may be related to ionoregulatory disturbances associated with the role of K^+^ in Na^+^ transport and homeostasis and in volume regulation.

### Calcium (Ca^2+^)

In freshwater osmoregulators, extracellular free [Ca^2+^] can range from 0.25 mM to 7.7 mM [163], whereas intracellular free [Ca^2+^] is much lower, 0.1 mM to 1 μM [163,164]. Although more extracellular and intracellular Ca is present, most is bound chemically to organic molecules, such as proteins and phospholipids [164] because intracellular free [Ca^2+^] beyond these limits has adverse neural, muscular, and cardiovascular effects [165]. Even low ionic strength freshwaters generally have minimum [Ca^2+^] of 2 μM to 230 μM and many exceed 2mM [161,163]. Therefore, the normal gradient across the apical membrane in freshwaters is down a concentration gradient, unlike most other ions.

#### Teleost fish.

Because the electrochemical gradient across the apical membrane favors Ca^2+^ entry, Ca^2+^ uptake across the gill apical membrane can occur by diffusion through a selective epithelial Ca^2+^-channel (ECaC) [23,70,163,166,167]. Such a channel has been identified on the gill apical membrane ionocytes in rainbow trout (Figure 1 and Table 2) [168] and zebrafish (Figure 2 and Table 2) [167,169]. The second step, Ca^2+^ transport across the basolateral membrane, occurs against an electrochemical gradient and requires active transport [163]. In freshwater fish, this occurs by the combined action of a plasma membrane Ca^2+^-ATPase (PMCA) and a Na^+^/Ca^2+^-exchanger (NCX) [23,70,163,167,170]. The reverse concentration gradient for Na^+^ across the basolateral membrane used by the NCX is in turn maintained by NKA, which is collocated in the same ionocyte type [171–174].

At the low levels of free intracellular Ca^2+^ in freshwater fish (0.1 – 1 μM), PMCA may be more important than the NCX because the PMCA activity exceeds that of NCX until the [Ca^2+^] approaches 1 μM, but only by a factor of 1.8 at 0.1 μM [175]. Increased cortisol, either as a result of exposure to low Ca^2+^ waters or by injection, increases Ca^2+^ uptake, primarily by increasing ionocytes with PMCA [176]. However, the NCX alone can maintain relatively low intracellular [Ca^2+^] (Supplemental Data, Table S5).

Increased NKA can be an adaption to low [Ca^2+^]. The ECaC also can increase in response to low [Ca^2+^]. In rainbow trout, the transporters for Ca^2+^ are located on β-type ionocytes (Supplemental Data, Table S5).

In addition to uptake through the gills, Ca^2+^ is absorbed from food through epithelia of the fish gastrointestinal system. This occurs by very similar transporters [177,178] (Supplemental Data, Table S5).

#### Crustacea.

Crayfish have been used frequently to study Ca^2+^ ionoregulation [179–182]. Because Ca is lost with molted exoskeletons, the Ca^2+^ requirement for Crustacea is substantial, and its uptake occurs mainly during postmolt following ecdysis [150,151,183–185]. During intermolt, crustacean Ca^2++^ requirements are met by reabsorption of up to 95% of the Ca^2+^ in the primary urine [186]. At ecdysis in *Procambarus clarkii*, only 17% of Ca^2+^ was retained, mostly in gastroliths, 42% was shed with the exuviae, and 41% had been excreted during the premolt stage [34]. Gastrolith Ca^2+^ was used to harden essential body parts, such as mouthparts and gastric ossicles for feeding and cactyles of the walking legs.

Because the movement is down a concentration gradient across the apical membrane, Ca^2+^ uptake is by an ECaC, which may be inhibited by verapamil [182]. However, this Ca^2+^ uptake is supplemented by at least 1 of 2 other exchangers (Figure 3 and Table 2). One is the amiloride-sensitive electrogenic H2Na(Ca)E, which was also discussed in relation to Na^+^, where Ca^2+^ competes with Na^+^ for external binding sites. This exchanger can transport either 2 Na^+^ or 1 Ca^2+^ because it has 2 external binding sites, and because of competition for these binding sites, these ions competitively and reciprocally inhibit the other’s passage [80,81,182,183,187]. This shared electrogenic exchanger for Na^+^ and Ca^2+^ is also affected by the interaction between pH and Na^+^ or Ca^2+^ [187]. As pH decreases in the external medium, the increased [H^+^] gradient across the apical cell membrane inhibits export of H^+^ and reduces influx of Na^+^ or Ca^2+^ [188]. There is also evidence that 1 Na^+^ can be exported by this exchanger in exchange for 1 Ca^2+^ [34,183], but H^+^ is the preferred substrate [189].

There is also an electroneutral 2Na^+^/Ca^2+^-exchanger that has a lower binding affinity for Ca^2+^ but a greater *V*_*max*_ [183]. Unlike the electrogenic H2Na(Ca)E, Na^+^ is the favored antiport substrate rather than H^+^, but this transporter can act as an electroneutral 2H^+^/Ca^2+^-exchanger, which is identifiable by the insensitivity to amiloride [183]. Because of the differences in the direction of movement of Ca^2+^ during different stages of molt, these transporters can move Ca^2+^ both ways across epithelial membranes [190].

On the basolateral or other internal membranes, Ca^2+^-ATPase generally maintains cytosolic Ca^2+^ at lower concentrations than found either in the water or in extracellular fluids [47,183,191,192]. This active cation transporter system is part of the family of P-type ATPases that also includes NKA. Although PMCA appears adequate to meet the transport requirements for Ca^2+^ during intermolt, the transport requirements are much greater during premolt and postmolt [182]. First, during premolt, Ca^2+^ is solubilized from the exoskeleton, transported through the epithelium to the hemolymph, and transferred for temporary storage. Then, during postmolt, the Ca^2+^ is mobilized from temporary storage, transported back across the epithelium, and deposited into the new exoskeleton [182]. Much of this increased transport across the basolateral membrane is accomplished by either an electrogenic 3Na^+^/Ca^2+^-exchanger or an electroneutral 2Na^+^/Ca^2+^-exchanger, both of which have greater transport capacities than PMCA [182,183]. However, PMCA is also up-regulated during the premolt and postmolt stages of *P. clarkii*, both in the gill and particularly in the antennal gland [193]. The electroneutral 2Na^+^/Ca^2+^-exchanger, the electrogenic 3Na^+^/Ca^2+^-exchanger, the amiloride-sensitive electrogenic H2Na(Ca)E, and Ca^2+^-ATPase, also occur in the labyrinth of the antennal gland, where they reabsorb Ca^2+^ from the primary urinary filtrate [186,189]. In *P. clarkii* during the postmolt period, a portion of this Ca^2+^ is stored in the antennal gland, in part as a source of Ca^2+^ to maintain extracellular and intracellular fluid homeostasis [194].

#### Aquatic insects.

Insect cuticle is not calcified and is mostly chitin and proteins [15,195]. Therefore, the calcium requirements of aquatic insects are much less than either crustaceans or molluscs, and aquatic insects may not require very high water [Ca^2+^]. Also, organic detritus, which is a common trophic resource for aquatic insects, may be a significant source of Ca^2+^ along with Mg^2+^ and K^+^ [196].

Research with larvae of the mosquito *A. aegypti* found that uptake of Ca^2+^ followed Michaelis–Menten kinetics and was inhibited by Ruthenium red, a PMCA inhibitor, but unlike the transporters for Na^+^ and Cl^-^, PMCA does not appear to occur in cells of the anal papillae (Supplemental Data, Table S5). Therefore, the location of divalent cation absorption is unclear.

As in other animals, much of the Ca^2+^ in the hemolymph appears to be bound to organic molecules, and there is relatively little free Ca^2+^ [197].

#### Mollusca.

The Ca^2+^ requirements for freshwater Mollusca can be substantial. In addition to needing Ca^2+^ for their shells, Unionidae form calcium concretions in their gills. These calcium concretions provide calcium for the shells of the glochidia, which develop from up to several hundred thousand eggs in a brooding chamber formed in the female’s gill [198–200].

*Dreissena polymorpha* maintained greater hemolymph [Ca^2+^] in pond water, which contained less Ca^2+^, than in artificial saltwater [60] (Supplemental Data, Table S5). The same authors [60] suggested that increased hemolymph [SO_4_^2-^] could be affecting the solubility of Ca^2+^ (SO_4_^2-^ and Ca^2+^ being the least soluble anion and cation, respectively).

Unionids also regulate ion concentrations in extrapallial fluids separately from the hemolymph (Supplemental Data, Table S5). In *Amblema plicata*, extrapallial fluid [Ca^2+^] was maintained at 3 to 4 times the water concentration.

*Lymnaea stagnalis* embryos have sufficient maternally supplied Ca^2+^ in the perivitelline fluid and gelatinous matrix surrounding the eggs for initial shell formation. However, after metamorphosis, Ca^2+^ concentrations decrease to less than ambient water concentrations. Embryos raised in Ca^2+^-free water exhibit reduced growth rates and longer times to hatch [201], suggesting that an external source of Ca^2+^ is needed for completion of embryonic development. Results with pharmacological agents (Supplemental Data, Table S5) [202] suggest that uptake of Ca^2+^ is accomplished both by an L-type ECaC and by Ca^2+^exchange for H^+^, with the H^+^ being supplied by the action of CA.

#### Effects on Ca^2+^ transport by other ions in the water.

Uptake of Ca^2+^ is inhibited by other divalent metals, because these divalent metals can also be transported by the ECaC across the apical membrane [203–206] and interact with PMCA on the basolateral membrane [207,208]. Competition between Ca^2+^ and other divalent metals results in inhibition of Ca^2+^ uptake by the divalent metals and vice versa [209–213] (Supplemental Data, Table S5). Variability in the relative affinity of PMCA for divalent metals versus Ca^2+^ among species explains, in part, the variation in these metals’ toxicity.

The electrogenic H2Na(Ca)E also transports divalent ions, such as Zn^2+^ and Cd^2+^, in addition to Ca^2+^ across the apical membrane [80] (Supplemental Data, Table S5). Therefore, competition for the external binding sites with Ca^2+^ explains the effect of water hardness on bioavailability and toxicity of these metals and Cu^2+^ in crustaceans [214]. This exchanger on vacuolar membranes is involved in metal sequestration into phosphate or sulfate concretions, a detoxification mechanism in crustaceans, and Ca^2+^ is sequestered into similar concretions until needed during molting [80]. In Crustacea, as in fish, Cd^2+^ and Ca^2+^ also compete for the ECaC (Supplemental Data, Table S5).

Although the location of Ca^2+^ transporters on *C. riparius* (midge) epithelia is unknown, Ca^2+^ uptake is inhibited by Cd^2+^ as in fish and Crustacea (Supplemental Data, Table S5). In Ephemeroptera and Trichoptera, which have tracheal gills, uptake mechanisms for divalent metals involve Ca^2+^ transporters associated with ionocytes (Supplemental Data, Table S5). Differences in the effect of verapamil, an inhibitor of ECaC, suggest differences in ECaC between these orders [215]. Also, differences in the relative affinity of the shared transporters for Ca^2+^ and Cd^2+^ or Zn^2+^ in these insects suggest that these transporters differ from those in Crustacea or fish (Supplemental Data, Table S5).

As in other freshwater animals, inhibition of divalent metal uptake by elevated ambient [Ca^2+^] suggests the presence of at least an ECaC on the gill apical epithelial membrane of Mollusca (Figure 5 and Table 2). Divalent metal uptake was inhibited by elevated Ca^2+^ in freshwater bivalves, and Ca^2+^ uptake was inhibited by divalent metals in *L. stagnalis* (Supplemental Data, Table S5). Elevated [Ca^2+^] in the hemolymph and extrapallial fluids occurs, because Cd^2+^ inhibits PMCA [216] and CA [217], which assist in movement of dissolved Ca^2+^ into the shell and into concretions in the soft tissues (Supplemental Data, Table S5).

As with Na^+^ and K^+^, inhibition of exchange of Ca^2+^ for H^+^ may explain the observation that negative net Ca^2+^ flux occurred in crayfish, such as *Orconectes propinquus*, exposed to pH 4.0 [218]. However, the same authors [218] also suggest that CaCO_3_ was demineralized from the crayfishes’ exoskeletons. Other crayfish can vary in their sensitivity to low pH (Supplemental Data, Table S5).

#### Synthesis.

It is clear that Ca^2+^ is required by aquatic organisms, and most Ca^2+^ uptake is from ambient water by epithelial ionocytes with a relatively common set of transporters, particularly the ECaC on the apical membrane and PMCA on the basolateral membrane. These similarities are evident from the similarities in uptake and competitive inhibition of Ca^2+^ uptake by a number of divalent metals. However, as with the transporters for Na^+^, some uncertainties exist for the aquatic insects because there is less research on this group (the transporter locations of the epithelial ionocytes have not been identified).

Particularly, Crustacea, which harden their exoskeleton with Ca^2+^ and then lose a significant proportion of that Ca^2+^ during molting, have a number of both apical and basolateral transporters that contribute to the uptake of Ca^2+^, particularly the potentially unique electrogenic H2Na(Ca)E, which has the shared potential for both Ca^2+^ and Na^+^ uptake. Because of this transporter’s role in Crustacea, there is potential for mutual competitive inhibition of Ca^2+^ and Na^+^ uptake. This may explain at least in part the observed relationship between hardness (i.e., [Ca^2+^]) and the toxicity of Na^+^-salts, such as NaCl and Na_2_SO_4_, in bioassays particularly with Crustacea [129,131–135]. This also explains the inhibition of Ca^2+^uptake by alterations in the concentration gradient for H^+^, either by decreased ambient pH or inhibition of CA activity in Crustacea [182,183,187,218]. Although freshwater Mollusca also accumulate Ca^2+^ in their shells, the turnover is not as great as in Crustacea, and the process appears to be more gradual and involves formation of Ca concretions in their soft tissues. However, research on the evolutionary relationships among functionally similar ion transporters has not been conducted for these invertebrate groups.

It is clear that Ca^2+^ contributes to the osmolarity of freshwaters, and elevated dissolved Ca^2+^ in intracellular and extracellular fluids can cause adverse effects. However, animals have very efficient homeostatic mechanisms to move Ca^2+^ out of the dissolved form and into other forms, such as organic molecules or particulates, like CaCO_3_ or Ca_3_(PO_4_)_2_, which can be stored in bone, concretions, shells, or exoskeleton. Most toxicity associated with Ca salts in a study of 2 cladocerans and a fish could be attributed to the salt’s associated anion; the Ca^2+^ cation itself was relatively nontoxic [128]. Conversely, there appears to be potential for the Michaelis–Menten uptake variables *K*_m_ and *V*_max_ for Ca^2+^ uptake to vary significantly among families and species [219]. Such physiological variation might underlie the differences in tolerance to low [Ca^2+^] and the resulting distribution of species along natural gradients, such as between soft and hard waters [220].

### Magnesium (Mg^2+^)

In extracellular fluids, Ca^2+^ is predominant over Mg^2+^, while within the cells, [Mg^2+^] is generally greater, often being at least the third most abundant cation [221] and [Ca^2+^] is relatively low [222]. Magnesium is a cofactor for enzymes that transfer phosphate groups, such as the ATPases, involved in energizing the pumps for H^+^ and Ca^2+^ and the exchanger for Na^+^ and K^+^ [221,223], and most cellular Mg^2+^ is associated with ATP [224]. As a result, Mg^2+^ deficiency has been implicated in imbalances of Ca^2+^, Na^+^, and K^+^, including shifts in the [K^+^]:[Na^+^] ratio in fish [225].

In its ionic geometry, Mg^2+^ is unusual among common cations, because it has a high hydrated radius but low ionic radius. Thus, Mg^2+^ requires a transporter protein with a large binding site and a dehydration mechanism to fit the cation through a relatively small pore [226]. In terrestrial vertebrate plasma membranes, Mg^2+^-channels in the solute carrier (SLC) 41 family have been identified and are homologous to an Mg^2+^ transporter family identified from prokaryotes [227]. However, no similar transporters have yet been identified in fish or aquatic invertebrates.

#### Teleost fish.

The freshwater fish absorb most required Mg^2+^ from their diet across the intestinal epithelia [228] (Supplemental Data, Table S6). Transport of Mg^2+^ across the apical membrane of the intestinal epithelia is by electrodiffusive transport along a negative potential gradient associated with K^+^ across the membrane, and transport can be inhibited by some divalent metals, particularly Co^2+^ and Ni^2+^ [229]. Transport across the basolateral membrane is by an anion/Mg^2+^-cotransport mechanism using membrane-permeable anions, such as Cl^-^, SO_4_^2-^, or NO_3_^−^ [230,231]. The amount of Mg^2+^ absorbed through the gill epithelia directly from the water is variable among fish species. Common carp (*Cyprinus carpio)*, for example, absorb at least 84% of Mg^2+^ from food through the intestinal tract, and the remainder is absorbed through the gills from the water [232]. Moreover, Mg^2+^ absorption by the gills can be insufficient to meet the need for Mg^2+^ (Supplemental Data, Table S6), particularly in quickly growing juvenile fish [233]. Conversely, tilapia fed a low Mg diet continued to grow (Supplemental Data, Table S6), which indicates that these fish absorb sufficient Mg^2+^ from the water through their gills [170]. Although 1.9 mM Mg^2+^ in water was sufficient to meet the requirements of juvenile rainbow trout fed a low Mg^2+^ diet, exposure to high [Mg^2+^] caused mortality (Supplemental Data, Table S5). However, that study did not investigate a mechanism for these adverse effects [234]. Loss of Mg^2+^ is primarily by renal excretion [235], but Mg^2+^ retention is efficient because the element is reabsorbed by the renal epithelium [236].

#### Crustacea.

In the freshwater prawn *Macrobrachium rosenbergii* hemolymph [Mg^2+^] peaked at 1.56mM Mg^2+^ at 2 d to 3 d premolt and decreased postmolt to a minimum during the intermolt period [237]. Similar patterns of variation in Mg^2+^ were observed in the exoskeleton, hepatopancreas, and whole body. Premolt [Mg^2+^] was similar to the water [Mg^2+^] (1.8 mM), suggesting that the increase resulted from movement of water and diffusion of Mg^2+^ across an increasingly permeable epithelial membrane and was perhaps associated with the role of Mg^2+^ as a cofactor in the ATPases involved in ion transport during the molt [237].

#### Aquatic insects.

In *Aedes campestris*, a mosquito that lives in alkali ponds dominated by NaHCO3 and, particularly in summer, Mg^2+^, the larvae drank the water (as do most animals living in hypertonic waters), and most of the ingested Mg^2+^ was absorbed into the hemolymph from the midgut [238]. Hemolymph [Mg^2+^] was maintained at <5 mM compared with a water [Mg^2+^] of up to 100 mM. This difference was because of the Mg^2+^ being excreted by the Malpighian tubules and salt gland [239]. Urine [Mg^2+^] exceeded the water [Mg^2+^] [238].

#### Mollusca.

Bivalves, possibly because they are filter feeders and are not as strong hyper-regulators in freshwaters, appear to depend more on absorption of Mg^2+^ through the gill epithelia from the water [240] (Supplemental Data, Table S6). When *D. polymorpha* were placed in different salt solutions, solutions lacking Mg^2+^ caused 100% mortality in less than 20 d, as did solutions lacking Na^+^ or Cl^−^. However, unionids and *Corbicula* appear to have ionoregulation mechanisms to avoid this depletion of Mg^2+^, because these bivalves have been maintained in deionized water for much longer [240].

#### Effects on Mg^2+^ transport by other ions in the water.

Toxicity testing in support of a biotic ligand model for acute Cu toxicity suggested that increased [Ca^2+^], [Na^+^], and [Mg^2+^] all increase the 48-h 50% effects concentration values for Cu^2+^ [214]. Although both Na^+^ and Ca^2+^ may competitively inhibit uptake of Cu^2+^ by the electrogenic H2Na(Ca)E, this transporter is not known to transport Mg^2+^, and the interaction between Mg^2+^ and Cu^2+^ is unclear. Moreover, Ni^2+^, which is known to move through Mg^2+^ transporters in prokaryotes [226], reduced Mg^2+^ influx and whole-body [Mg^2+^] in *Daphnia* spp. exposed to 0.8 μM to 59 μM Ni^2+^ [241,242]. In *L. stagnalis*, soft tissue [Mg^2+^] decreased after acute and chronic exposures to 8 μM and 0.29 μM Ni^2+^, respectively [242,243]. These observations suggest the presence of an Mg^2+^ transporter similar to those found in prokaryotes.

#### Synthesis.

Although the role of Mg^2+^ in cells, particularly as a cofactor for ATPases, is clear, relatively little has been published on the transepithelial transport of Mg^2+^ in aquatic animals. Much of the Mg^2+^ in juvenile and adult freshwater fish appears to be obtained from food, but there is interspecific variability. Studies of crustaceans suggest that transport of Mg^2+^ from ambient water across the gill epithelium may be down a concentration gradient, and some studies of Mollusca agree. However, the study of *D. polymorpha* in dilute pond water suggests a more active Mg^2+^ transport mechanism across the gill epithelium [240]. Several studies suggest that Mg^2+^ can cause mortality at greater concentrations [234], and the relative toxicity of Mg^2+^ has been ranked as similar to HCO_3_^−^ but less than K^+^ [128].

Several toxicological papers have suggested that Mg^2+^ has a role in the uptake and toxicity of metals, apparently based mostly on the protective effect of water hardness on some metals and experiments conducted with Ag^+^ and Cu^2+^ [214,244,245]. However, no effects on accumulation of Ag^+^ measured as total gill [Ag^+^] were found for water [MgSO_4_] of 0mM to 210 mM or water [CaSO_4_] of 0mM to 8.6 mM in rainbow trout [244]. No relationship between [Ca^2+^]:[Mg^2+^] ratios and Cu^2+^ toxicity was found for fathead minnows *C. dubia*, or *Gammarus* spp. [245]. Other studies have shown that uptake of both Ag^+^ and Cu^2+^ occurs by an Na^+^ transporter and not the ECaC, unlike other divalent metals, and Mg^2+^ should have little effect on the uptake of these 2 metals, particularly if one assumes a relationship with Ca^2+^. However, there is no evidence that Mg^2+^ is transported by a Ca^2+^ transporter. Moreover, it is unclear why Cu^2+^ toxicity, as measured by an acute median lethal concentration (LC50), increases in fingerling rainbow trout and decreases in *D. magna* with increasing [Mg^2+^] [245]. More research is needed on Mg^2+^ transporters in freshwater animals to better understand the ionoregulation of this ion.

### Chloride (Cl^−^)

Similar to uptake of Na^+^ in exchange for H^+^, uptake of Cl^−^ is in exchange for an ion that is a product of metabolism, HCO_3_^−^. Both vertebrate and invertebrate apical membranes possess a Cl^−^/HCO_3_^−^-exchanger (or anion exchanger) [3,23,75,166,246–249]. Although Cl^−^ uptake is clearly independent of the Na^+^ uptake [246], VHA activity appears to enhance uptake of Cl^−^ by the anion exchanger, particularly when the [Cl^−^] is very low [250].

#### Teleost fish.

Research on fish [251] first suggested that HCO_3_^−^ was exchanged for Cl^−^. Moreover, inhibition of CA, which catalyzes formation of HCO_3_^−^ and H^+^, decreased the uptake of Cl^−^ in rainbow trout gills [252]. The uptake affinity and capacity for zebrafish of Cl^−^ was more than 3 times greater in soft water (43 μM Cl^−^) than in hard water (1625 μM Cl^−^) [250], and uptake was inhibited by bafilomycin, a VHA inhibitor. Inhibition of Cl^−^ uptake by ethoxzolamide, a CA inhibitor, in both hard and soft water, adds evidence that Cl^−^ uptake occurs by exchange for HCO_3_^−^. Moreover, in soft water this exchange appears to be inhibited by reduction of the concentration gradient for HCO_3_^−^ across the apical membrane [250]. Molecular analyses of zebrafish implicate anion transporters of the SLC26 family as the anion exchanger most involved in Cl^−^ transport and located these transporters on the apical membrane of base-excreting ionocytes (Figure 2 and Table 2) [253] (Supplemental Data, Table S7). On the yolk-sac membrane of tilapia larvae, the proportion of active ionocytes (indicated by the presence of concanavalin-A), which also stained for NKA, increased in larvae acclimated for 48 h to low [Cl^−^] artificial freshwater (2–7 μM) compared with larvae in high Cl^−^ artificial freshwater (7500–7900 μM) [254]. These ionocytes were inactivated at high Cl^−^ by being covered by pavement cells. This interaction, which also involves CA, occurs because of an ion-transport metabolon [255] (Supplemental Data, Table S7). However, variation in anion exchanger among fish species is suggested by pharmacological differences between goldfish (*Carassius auratus*) and neon tetra (*Paracheirodon innesi*) [256] (Supplemental Data, Table S7). In addition to the anion exchanger, NCC, which is discussed in relation to Na^+^ uptake (*Sodium (Na*^+^) - *Other Na*^+^ *transporters*), uses the Na^+^ gradient created by combined action of NKA and the NBC on the basolateral membrane to transport Cl^−^ against its concentration gradient across the apical membrane between freshwater and the cytosol [99,101,166].

Some Cl^−^ uptake can occur from food in the gastrointestinal system. Rainbow trout absorbed approximately 80% of the Cl^−^ in a single experimental meal that supplied 2.65 mmol Cl^−^ kg^−1^ fish body mass [257].

Cytosol [Cl^−^] in epithelial ionocytes is maintained at higher levels than in extracellular fluids and allows Cl^−^ transport across the basolateral membrane by a Cl^−^-channel [3,166]. Limited research has been conducted to characterize such basolateral channels in fish gill ionocytes, and 2 types of Cl^−^-channels have been identified. One similar to the Cl^−^ channel 3 has been identified in spotted green pufferfish (*Tetraodon nigroviridis*) [258] and another is related to the cystic fibrosis transmembrane regulator [166,259]. In rainbow trout, a maxi Cl^−^-channel has been described in pavement cells that may fulfill this function [260]. Although most research has been conducted on terrestrial vertebrates, the SLC26 family includes diverse anion exchangers that couple exchange of Cl^−^ with other anions, including HCO_3_^−^. The family also includes several transporters that can act as either an obligatory coupled exchanger or as an ion channel [261].

#### Crustacea.

As a major hemolymph osmolyte along with Na^+^, Cl^−^ uptake increases (*J*_net_^Cl^ = 250–800 μmol kg^−1^ h^−1^) during the postmolt period in crayfish, primarily to counteract the dilution of hemolymph ion concentrations by water absorption and the increase in body volume following ecdysis [150,151]. Uptake occurs in exchange for HCO_3_^−^ and is reduced by acetazolamide, which inhibits CA [79]. As with Na^+^, renal filtration and reabsorption of Cl^−^ in the antennal gland exceeds fluxes through the gills during intermolt because recycling of these ions is more energy efficient [34]. In addition to the anion exchanger, NCC and NKCC, which is discussed in relation to Na^+^ uptake (*Sodium (Na*^+^) - *Other Na*^+^ *transporters*), have been detected pharmacologically in neonates and adults, respectively, of the cladoceran *D. magna* [102].

In freshwater Decapoda, gas and ion exchange is separated among different gill filaments and lamina on the branchiae [262,263] (Supplemental Materials, Table S6). This separation of functions between gill filaments and lamina also separates the 2 functions of CA [264,265], shifting the equilibrium toward HCO_3_^−^ for exchange of Cl^−^ at the apical membranes of the lamina ionocytes, while shifting the equilibrium toward CO2 at the basolateral membranes of the second filament type for excretion across the epithelia [266]. Most research with Decapoda indicates that inhibition of CA affects Cl^−^ uptake more than Na^+^ uptake [267]. Similarly, in the cladoceran *D. magna, 2* types of gill epithelial cells, called dark and light cells, are hypothesized to be involved in ionoregulation and gas exchange, respectively [268].

#### Aquatic insects.

Stobbart [269] provided evidence for exchange of Cl^−^ for HCO_3_^−^ during uptake of Cl^−^ across the anal papillae by larvae of *A. aegypti* via active transport mechanisms [270]. Application of methazolamide (CA inhibitor), 4-acetamido-4’-isothiocyanatostilbene-2-2’-disulfonate (anion exchanger inhibitor), or 4,4’-diisothiocyanatostilbene-2,2’-disulfonic acid (DIDS; anion exchanger inhibitor) to the anal papillae of *A. aegypti* reduced Cl^−^ uptake by 79%, 80%, and 40%, respectively [271]. Bumetanide (NKCC) had no effect on Cl^−^ uptake, supporting the role of anion exchanger but not an NKCC in Cl^−^ uptake across the anal papillae (Figure 4 and Table 2). In *Anopheles albimanus*, an apical anion exchanger on dorsal anterior rectal cells in the rectum uses HCO_3_^−^ produced by CA in either ventral anterior or posterior rectal cells to exchange for Cl^−^, resorbing this ion from the insect’s primary urine [272].

One factor in the sensitivity of freshwater-restricted mosquitoes such as *Culex quinquefasciatus* to increased NaCl salinity may lie in the permeability of their anal papillae to influx of Cl^−^ compared with euryhaline species such as *Culex tarsalis* [273] (Supplemental Data, Table S7). Similar variation in permeability was observed among euryhaline and freshwater of caddisflies (Supplemental Data, Table S7).

However, the Cl^−^ uptake process is clearly independent from that of Na^+^. When *A. aegypti* larvae were transferred from freshwater to 30% saltwater and back [90], the *K*_m_ for Na^+^ uptake was altered, whereas that for Cl^−^ remained relatively constant, and recovery of *V*_max_ for Na^+^ was much slower than that for Cl^−^ (20 h vs 5 h) when the larvae were returned to freshwater.

In Ephemeroptera, autoradiography and histochemical precipitation showed active Cl^−^ absorption across a steep concentration gradient [274] by ionocytes on the tracheal gill epithelia [275]. The Cl^−^ uptake rate was directly related to the water hypotonicity, showing that the cells were involved in ionoregulation. Similar results were observed for ionocytes in aquatic Hemiptera nymphs in Notonectidae and Naucoridae [276].

As with Na^+^, the anal papillae of the midge C. *riparius* are a site of Cl^−^ ion uptake [89]. The anal papillae of marsh beetle larvae, *Elodes minuta* and *Odeles marginata* (Coleoptera, Scirtidae), are also a site of active Cl^−^ uptake [277]. However, none of these authors measured HCO_3_^−^ and did not demonstrate exchange of these anions. Similarly, chloride epithelia in the rectal chamber of dragonfly nymphs (*Uropetala carovei* and *Aeshna cyanea*) were involved in active uptake of Cl^−^ in addition to Na^+^ [278–281], although the mechanisms for Cl^−^ uptake were not elucidated.

#### Mollusca.

Although unionids have anion exchangers [95], these river mussels maintain approximately equal amounts of Cl^−^ and HCO_3_^−^ in their extracellular fluids, and pH maintenance relies more on exchange of Na^+^ and H^+^ [282] (Figure 5 and Table 2). Transport of Cl^−^ in *Ligumia subrostrata* was inhibited by DIDS (anion exchanger inhibitor) and thiocyanate (Cl^−^ transporter inhibitor), but not furosemide (NKCC) [283]. As with other freshwater groups, exchange of HCO_3_^−^ for Cl^−^ is independent of Na^+^ in freshwater mussels (Supplemental Data, Table S7). Also, hemolymph [Cl^−^] increases and become isoionic with increasing salinity (Supplemental Data, Table S7). It was hypothesized that HCO_3_^−^-dependent ATPase is involved in this Cl^−^/HCO_3_^−^ exchange in 2 freshwater gastropods (Architaenioglossa), *Viviparus contectus* and *Pomacea canaliculata* [284], but reanalysis suggests the authors may have misinterpreted the activity of VHA, which in fish and decapods removes H^+^ and maintains the supply of HCO_3_^−^ from CA-catalyzed hydrolysis of CO_2_ to the anion exchanger [250,285] (Supplemental Data, Table S7).

#### Effects on Cl^−^ transport by other ions in the water.

In fish and crustaceans, exposure to pH of 4.0 to 4.3 causes net flux of Cl^−^ from the extracellular fluids along with decreased [HCO_3_^−^] and pH; *P*_CO2_ is unchanged or increased (Supplemental Data, Table S7). Current models suggest that inhibition of HCO_3_^−^ production by decreased extracellular pH decreases the availability of HCO_3_^−^ to exchange for Cl^−^. At the same time, HCO_3_^−^ is retained to maintain extracellular acid–base balance.

Uptake of NO_2_^−^ can occur through the anion exchanger in both fish and Crustacea. This transporter has an affinity for several anions in addition to Cl^−^, including Br_−_ but not SO_4_^2−^ or PO_4_^3−^ [286,287]. It competitively inhibits uptake of Cl^−^ and reduces blood [Cl^−^] while blood [NO_2_^−^] increases [288,289]. Conversely, elevated Cl^−^ or BP can inhibit uptake of NO_2_^−^ [287]. In crayfish, increased hemolymph [HCO_3_^−^] as a result of induced hypercapnia increased NO_2_^−^ uptake because HCO_3_^−^ is the counter ion for anion exchanger [286] (Supplemental Data, Table S7). In fish, NO_2_^−^ has an added effect on respiration (Supplemental Data, Table S7).

In rainbow trout and several tetra species acutely exposed to Ag^+^, Cl^−^ uptake decreased, as did Na^+^ uptake [113,290]. There was also a decrease in CA activity, which would limit the supply of HCO_3_^−^ for exchange with Cl^−^.

#### Synthesis.

The most important anion for osmoregulation in freshwater animals is Cl^−^. Although its transport across gill epithelial membranes is independent of Na^+^ transport, there are a number of parallels because the transporters for Cl^−^ and Na^+^ exchange these ions for HCO_3_^−^ and H^+^, respectively. In turn, HCO_3_^−^ and H^+^ are produced by hydration of CO_2_ by CA and have opposing roles in maintaining acid-base balance in intracellular and extracellular fluids. In most freshwater animals, an anion exchanger moves Cl^−^ across the apical epithelial membrane, while some type of Cl^−^-channel moves Cl^−^ across the basolateral epithelial membrane. As with H^+^ and the NHE or VHA, elevation of ambient water [HCO_3_^−^] can inhibit Cl^−^ uptake by increasing the [HCO_3_^−^] gradient across the apical membrane.

Elevation of a number of other ions in the water has been shown to inhibit uptake of Cl^−^ by the anion exchanger: H^+^ or low pH inhibit Cl^−^ uptake because they appear to inhibit VHA by increasing the H^+^ gradient across the apical epithelial membrane. Also, removal of H^+^ helps maintain the [HCO_3_^−^] gradient required for the anion exchanger [255]. Anions such as NO_2_^−^ and BP inhibit uptake of Cl^−^ by competing for attachment sites on the exchanger.

Because of the importance of Cl^−^ in osmoregulation, any effects associated with elevated Cl^−^ appear to be related to osmotic imbalances. Particularly in stenohaline freshwater animals, extracellular [Cl^−^] generally remains greater than that in increasingly saline water until the water becomes isotonic with the hemolymph. Then, at greater salinities, extracellular [Cl^−^] either increases and remains approximately isotonic with the water (as in invertebrates) or becomes hypotonic with the water (as in fish). At this point mortality often becomes significant [139,140]. As a result, Cl^−^ ranks low in relative toxicity [128].

### Bicarbonate (HCO_3_^−^)

As in all aerobic organisms, CO_2_ is produced by respiration and eliminated from the bodies of freshwater animals. However, because CO_2_ is more water soluble than O_2_, the greater ventilation rates of gas-exchange surfaces in water-breathing organisms render changes in *P*_CO2_ relatively ineffective at controlling intracellular and extracellular pH [291]. Although a significant proportion of molecular CO_2_ is still eliminated by passive diffusion across the epithelia [291], CA in the cytosol catalyzes the hydration of CO_2_ and shifts the equilibrium to H^+^ and HCO_3_^−^ making these ions available for exchange across the apical membrane and for regulation of the concentrations of Na^+^ and Cl^−^, respectively, [292]. This exchange of acid–base equivalents also regulates the acid–base balance of intracellular and extracellular fluids [15,292,293].

#### Teleost fish.

In a 2-substrate enzyme model, the rate of ion transport is dependent on the availability of the internal substrate, which in this case is H^+^ or HCO_3_^−^, as well as the external substrates, Na^+^ and Cl^−^, respectively [294,295]. If metabolic acidosis occurs, [H^+^] increases while [HCO_3_^−^] decreases, which in turn increases exchange of H^+^ for Na^+^ and decreases exchange of HCO_3_^−^ for Cl^−^, thereby increasing acid excretion and decreasing hemolymph [Cl^−^] (Supplemental Data, Table S8). The decreased exchange of HCO_3_^−^ for Cl^−^ may occur because the anion exchanger on ionocyte apical membranes (Figure 1 and Table 2) are physically covered by adjacent pavement cells [296]. However, the decrease in blood [Cl^−^] could result from a reversal of HCO_3_(influx)/Cl^−^(efflux) exchange by the apical anion exchanger based on observations that compensation for decreased pH from hypercapnia occurred more quickly in waters with greater [HCO_3_^−^] (5.3 mM) than in those with lower [HCO_3_^−^] (0.13 mM) [297]. If metabolic alkalosis occurs, H^+^ is retained and HCO_3_^−^ is exchanged for Cl^−^, increasing base excretion [298]. Together, this negative feedback system regulates acid-base balance [295]. Under balanced acid-base status, uptakes of Na^+^ and Cl^—^ are equal to maintain these conditions [299].

However, at least among rainbow trout, American eel, and brown bullhead *(Ameiurus nebulosus)*, there is variation in whether a decrease in pH (i.e., systemic acidosis) is countered by decreased excretion of HCO_3_^—^ or increased H^+^ excretion [291,292,299,300], and interspecies differences occur in the relative importance of Na^+^/H^+^ versus Cl^—^/HCO_3_^—^ exchange to maintaining acid-base balance (Supplemental Data, Table S8).

The transporters involved export of HCO_3_^—^ or H^+^ across the apical membrane and in acid–base regulation are also segregated among different epithelial cell types [21,66,70,71,249,255,292,301]. Depending on the fish species, various ion transporters may be segregated among 2 to 4 cell types [71,301–303] (Figures 1 and 2). This segregation allows these ions to be moved between the cytosol and hemolymph for maintaining acid–base balance and for use in transporters (e.g., NBC) on the basolateral membrane [99]. Moreover, this variation in the relative importance of Na^+^/H^+^ versus Cl^−^/HCO_3_^—^ may explain the variation in response to acidosis.

Although they did not investigate the physiological mechanisms, Harper et al. [304] assessed the toxic effects of NaHCO_3_ as a major constituent of produced waters from coalbed natural gas wells. The 96-h LC50 values for the larval stages (1–22 d posthatch [dph]) of white sucker (*Catostomus commersonii*), fathead minnow, walleye (*Sander vitreus*), rainbow trout, and northern pike (*Esox lucius*), respectively, were estimated as 13.6 mM, 38.5 mM, 58.9 mM, 93.4 mM, and 131.1 mM NaHCO_3_. For fathead minnows, the chronic LC50 for survival at 37 dph was 6.0 mM NaHCO_3_, whereas a 7-d growth test that began with 2-dph larval fish had a 20% effect concentration of 8.2 mM NaHCO_3_ [305]. The lower toxicity values appear to result from effects of HCO_3_^−^ rather than Na^+^.

#### Crustacea.

During premolt in crayfish, excretion of CO_2_ increases above intermolt rates. Immediately following ecdysis, CO_2_ excretion decreases [306], probably reflecting accumulation of HCO_3_^−^ with Ca^2+^ as CaCO_3_ in the exoskeleton [150].

When tissue and hemolymph acidosis (i.e., decrease of 0.1–0.5 pH units by 3 h) was induced by exposing *Pacifastacus leniusculus* to hyperoxia (*P*_O2_ = 67 kPa), this acidosis was corrected by metabolic accumulation of HCO_3_^−^ + CO_3_^2–^ by 48 h [59]. Consequent to this accumulation of HCO_3_^−^ + CO_3_^2–^ was a small net loss of Cl^−^ (Supplemental Data, Table S8).

A number of studies have shown that HCO_3_^−^ can cause adverse effects to Cladocera and freshwater shrimp (Table 3). The effects for NaHCO_3_ occur at lower molar concentrations or specific conductivity than those for NaCl, showing that there were effects in excess of those resulting from Na^+^ or Cl^−^.

#### Aquatic insects.

The role of HCO_3_^−^ and H^+^ in maintaining acid–base balance has not been directly investigated in aquatic insects. However, logically these ions likely play a role similar to that in other aquatic animals. One question that might be important in the case of aquatic insects is whether the CO_2_ is supplied by respiration in the individual ionocytes or whether there is some other mechanism that moves CO_2_ to the ionocytes, because gas exchange in most aquatic insects occurs separately via the tracheal system.

Studies of the adverse effects of HCO_3_^−^ in water for aquatic insects are few. A 96-h LC50 for *Chironomus dilutus* was 58.9 mM NaHCO_3_ [304]. An analysis was conducted of macroinvertebrates in streams of the central Appalachians, where the most abundant elevated anions were HCO_3_^−^ and SO4^2–^, and showed a field-based species sensitivity distribution of 95% extirpation concentration values for specific conductivity (a measure of the total ion concentration), which for aquatic insects ranged from 150 μS/cm to >11 000 μS/cm. The results also showed a 5% hazardous concentration of 300 μS/cm [307].

#### Mollusca.

In the unionid *Anodonta cygnea*, CA catalyzed hydration of CO_2_ supplies HCO_3_^−^ for transport of Cl^−^ [95]. Short-circuit current across the outer mantle epithelium of *A. cygnea* was inhibited by acetazolamide (CA inhibitor), DIDS (anion exchanger inhibitor), and dinitrophenol (VHA inhibitor), showing that the short-circuit current was affected by the [CO_2_] and [HCO_3_^−^] as well as [H^+^] in the shell-side solution and that acid–base transport was inhibited [308]. Therefore, acid-base balance across this outer mantle epithelium was maintained by both anion exchanger and VHA (Figure 5 and Table 2). However, in *D. polymorpha*, HCO_3_^−^ was not excreted to counter an increase in pH (Supplemental Data, Table S8).

Comparing an estuarine osmoconformer, *Rangia cuneata*, with 2 freshwater hyper-regulators, *L. subrostrata* and *Corbicula fluminea*, CA activity in the gills increased among these species to the extent that the animals’ hemolymph [Na^+^] and [Cl^−^] were regulated above the ambient water concentrations. This reflected the increased Na^+^/H^+^ and Cl^−^/HCO_3_^−^ exchange to maintain these gradients [309] (Supplemental Data, Table S8).

In molluscs, CA plays a role in exchange of Ca^2+^ between the dissolved ion form and solid forms, such as in concretions and the shell, by affecting the equilibrium between CO_3_^2–^ and HCO_3_^−^. Inhibition of CA increased [Ca^2+^] in the hemolymph and extrapallial fluids (Supplemental Data, Table S8).

A study of the effects of NaHCO_3_ as a constituent of produced water from coalbed natural gas wells included the unionid *Lampsilis siliquoidea*. Using absence of foot movement during a 5-min observation as the indicator of mortality, the 96-h LC50 and 7-d LC50 for juveniles were 13.8 mM and 12.6mM NaHCO_3_, respectively [304,305].

#### Synthesis.

HCO_3_^−^ and H^+^ play a primary role in acid-base balance of extracellular and intracellular fluids in freshwater animals. Because elevated [H^+^] in ambient waters inhibits exchange of H^+^ for Na^+^ by increasing the [H^+^] gradient across the apical epithelial membrane, elevation of ambient water [HCO_3_^−^] can also inhibit exchange of HCO_3_^−^ for Cl^−^, apparently in the same manner, by increasing the [HCO_3_^−^] gradient against which the anion exchanger is excreting HCO_3_^−^. Inhibition of HCO_3_^−^ excretion would have at least 2 direct effects. First, it could cause metabolic alkalosis, increasing hemolymph or blood pH. Second, it could, in particular, cause decreased hemolymph or blood [Cl^−^]. Adverse effects, including impaired reproduction and mortality, have been identified in some fish, crustaceans, aquatic insects, and molluscs. In terms of its acute relative toxicity to standard bioassay species, HCO_3_^−^ ranked only behind K^+^ [128].

### Sulfate (SO_4_^2−^)

Sulfate appears to be an impermeant anion to the gills of freshwater fish [310,311], crustaceans [312,313], and unionid molluscs [25]. However, some research has identified SO_4_^2−^ transporters, particularly in ionocytes of the gastrointestinal and renal systems.

#### Teleost fish.

Although similar data are lacking for fish, active transport of SO_4_^2−^ into *Bufo bufo* (European toad) has been localized to ionocytes in the skin [314] (Supplemental Data, Table S8). Larsen and Simonsen [314] suggested that SO_4_^2−^ was transported in exchange for HCO_3_^−^ via an exchanger similar to, if not the same as, the anion exchanger. Also, the transported ion is monovalent (i.e., XSO_4_^−^), and the antiporter is energized by movement of HCO_3_^−^ down its concentration gradient as in the anion exchanger.

Several SO_4_^2−^ transporters have been identified in the SLC26 and SLC13 families of anion transporters [315]. Although most research on these transporter families has been on terrestrial vertebrates [261], transporters that are homologs of the human anion exchanger SLC26a1 have been identified in the kidney of Japanese eel (*Anguilla japonica*) and rainbow trout [316,317]. In rainbow trout that were infused with solutions of MgSO_4_ or Na_2_SO_4_, most of the SO_4_^2−^ load was excreted by the renal tubules [235].

In Japanese eels in freshwater, most regulation of SO_4_^2−^ occurs by resorption in the kidney’s proximal tubule, the SO_4_^2−^ possibly being derived from metabolism of sulfur-containing compounds [316]. In these epithelial cells, 2 transporters are involved in SO_4_^2−^ transport. First, a 3Na^+^/SO_4_^2−^-cotransporter (SLC13s1) moves both Na^+^ and SO_4_^2−^ across the apical brush-border membrane driven by the Na^+^ gradient produced by NKA on the basolateral membrane. Then, the SO_4_^2−^ is moved across the basolateral membrane by an SO_4_^2−^/2HCO_3_^−^-exchanger (SLC26a1), which is energized by an SO_4_^2−^ gradient across the basolateral membrane [316]. In Japanese eels, SO_4_^2−^ may play a role in osmoregulation in freshwater because serum SO_4_^2−^ is elevated in freshwater (~19 mM vs ~1 mM in saltwater), whereas Cl^−^ is reduced compared with the Japanese eels in saltwater or other fish in freshwater [316,318].

#### Crustacea.

In a study of Na^+^ absorption from water by *Austropotamobius pallipes* using various anion salts, including Na_2_SO_4_ [312], there was no penetration of gill epithelial membranes by SO_4_^2−^. A review of a number of studies that used H_2_SO_4_ to study the physiological effects of acidification [313] found that, at most, SO_4_^2−^ permeates very slowly across gill epithelial membranes.

Like a number of other ions, there was a negative net flux of SO_4_^2−^ from the crayfish *O. propinquus* exposed to pH 4.0 [218]. In the absence of SO_4_^2−^ transporters on the gill epithelia, increased efflux may be because of increased paracellular permeability at low pH.

#### Aquatic insects.

In *A. campestris*, a mosquito that lives in hyperosmotic inland waters, including saline lakes dominated by Mg^2+^ and SO_4_^2−^, larvae maintained hemolymph [SO_4_^2−^] at approximately 2 mM to 7 mM, despite water [SO_4_^2−^] that was up to 80 mM. Ingestion of this water resulted in absorption of the SO_4_^2−^ through the midgut [319]. This balance resulted from the excretion of fluids containing high [SO_4_^2−^] (up to ≈95 mM) by the mosquito’ s Malpighian tubules, where active transport of SO_4_^2−^ occurs.

#### Mollusca.

In unionid mussels, SO_4_^2−^ is accumulated rather slowly (e.g., 0.04 μmol g^−1^ dry wt h^−1^) and is a relatively impermeant anion [25]. It occurs in the range of 0.23 mM to 0.30 mM in hemolymph of unionid mussels and is metabolized to form amino acids and mucopolysaccharides. The hemolymph [SO4^2−^] of 0.23 mM, although relatively low for an anion, was approximately 8 times the [SO_4_^2−^] in pond water [25].

Using the radioisotope ^35^SO_4_ to measure transport of SO_4_^2−^, uptake of SO_4_^2−^ by the unionid *Toxolasma texasiense* from pond water was inhibited by addition of DIDS, an anion exchanger inhibitor [25]. Furthermore, clearance of ^35^SO_4_ injected into *T. texasiense* individuals was 16% of a simultaneously injected chemical, polyethylene glycol, used to measure renal filtration rates, indicating that SO_4_^2−^ was reabsorbed by the kidney [25]. Similar results were obtained for pond water-acclimated *D. polymorpha* (Supplemental Data, Table S9). Although the transporter for SO_4_^2−^ is still unclear, the inhibition of the ion’s uptake by DIDS, an anion exchanger inhibitor, suggests an anion exchanger (Figure 5 and Table 2) [25,320].

#### Effects on SO_4_^2−^ transport by other ions in the water.

Hōbe [321] observed net influx of SO_4_^2−^ in white sucker and rainbow trout exposed to natural soft water acidified to pH 4.0 by addition of H_2_SO_4_ but not in controls (pH 6.8). Based on current models, this influx appears to have resulted from passive movement of SO_4_^2−^down a concentration gradient created by the addition of H_2_SO_4_ to the soft water ([SO_4_^2−^] was 0.20 mM to 0.40 mM in acidified soft water and 0.07 mM to 0.10 mM in control soft water) across tight junctions made more permeable by the low pH and low Ca^2+^ conditions, rather than the action of any transporter. As in other studies of acidic, soft waters, net effluxes of Na^+^, K^+^, Ca^2+^, Mg^2+^, and Cl^−^ were also observed.

#### Synthesis.

Data for various animals suggest that SO_4_^2−^ transporters may not be present on the gill epithelia of most freshwater animals because of the slow permeation of these epithelia. However, in the European toad, there is evidence for active transport of SO_4_^2−^ across the skin epithelia, which are at least functionally equivalent to gill epithelia. There is also evidence for active transport of SO_4_^2−^ from ambient water to the hemolymph in freshwater mussels, but the location of a transporter is not clear. In general, SO_4_^2−^ has not been well studied in freshwater animals compared with other ions.

Soucek and Kennedy [133] and Soucek [132] attempted to assess the relationships among hardness, Cl^−^, and acute effects associated with SO_4_^2−^ in bioassays with 2 crustaceans, although the dose consisted of solutions of the salt Na_2_SO_4_. Physiological research has generally suggested that gill epithelial membranes are impermeant to SO_4_^2−^, although SO_4_^2−^ transporters may occur in the renal system. A possible mechanism is related to the cation Na^+^. In the electrogenic H2Na(Ca)E on the crustacean gill, Ca^2+^ and Na^+^ can compete for attachment sites on this transporter, which would result in the positive relationship between hardness and the LC50 values for *Hyalella azteca* and *C. dubia*.

It is clear, however, that SO_4_^2−^ transporters are present in ionocytes of the renal system in fish where these transporters resorb SO_4_^2−^. The question remains whether there are SO_4_^2−^ transporters in either the gill or gastrointestinal epithelia of any freshwater animals that might be affected by elevated [SO_4_^2−^] as a result of anthropogenic sources, such as those associated with mining [322]. Although elevated [SO_4_^2−^] is present in freshwaters associated with acidic precipitation or acid mine drainage, the effects of low pH have been more obvious and, consequently, well studied in both the physiological and toxicological literatures. Thus, the potential for any effects related directly to SO_4_^2−^ has been overlooked.

## DISCUSSION

In general, there appears to be a significant level of evolutionary conservatism in ion transporters among the 4 freshwater groups reviewed. Where there are physiological data, transporters, such as VHA, anion exchanger, NKA, PMCA, and NCX, appear to be functionally similar, and where there are genomic data, these transporters usually belong to identified protein families that occur in species across various phyla. However, there are also differences (e.g., the electroneutral NHE in teleost fish compared with the electrogenic H2Na(Ca)E in crustaceans that helps crustaceans to meet their greater requirements for Ca^2+^ uptake during molting). Also, when the apparent relative affinities of the Ca^+^-channels of the groups are compared, it appears that the affinities for Cd^2+^ and Zn^2+^ in fish, crustaceans, and molluscs are greater than those for Ca^2+^, whereas in aquatic insects, the affinities for divalent metals may be less than those for Ca^2+^. There may also be differences between some aquatic insects and these other animal groups in the apical Na^+^-channel that also transports metals like Ag^+^ and Cu^2+^.

The most widespread difference of these transporters among species, and 1 that is more subtle, is the affinity and capacity of each transporter for its ion as measured by *K*_*m*_ and *V*_*max*_. These 2 variables seem to have the potential to describe the variation in the tolerance of different species particularly to low ion concentrations. However, few studies have compared these variables for an ion measured under the same conditions among more than 1 species in the same freshwater group (e.g., goldfish, zebrafish, and ayu *[Plecoglossus altivelis];* [219]), and no studies have done this among fish and various invertebrate groups (e.g., rainbow trout with crayfish).

Among the individual major ions, K^+^ appears to have the most potential for causing toxic effects to freshwater animals if water concentrations of this ion are elevated. The relative acute toxicity of K^+^ was ranked as the highest of the major ions studied [128], and it has been used as a molluscicide. Survival of *polymorpha* was greatest when the [K^+^]:[Na^+^] ratio was very low (0.01–0.02) [36]. The counterion forNKA is K^+^, which is important to maintaining the relative [Na^+^] in intracellular fluids and the blood or hemolymph, suggesting that this toxicity is related to an ionoregulatory imbalance. Lack of data on the identity of an apical membrane K^+^ transporter makes the details of such an imbalance unclear. However, K^+^ usually has the lowest concentrations in freshwaters of these major ions [161].

The toxic effects of H^+^ have been well studied, particularly in relation to the effects of acid precipitation on stream and lake assemblages, but the mechanistic link to inhibition of Na^+^ uptake by the NHE or VHA and apical Na^+^-channel and decreases in intracellular and extracellular [Na^+^] has seldom been mentioned in the toxicological literature. Similarly, elevated ambient HCO_3_^−^ appears to inhibit uptake of Cl^−^ by the anion exchanger and decreases intracellular and extracellular [Cl^−^]. Elucidation of the details of this mechanism are needed because HCO_3_^−^ ranked relatively high among the major ions in terms of toxicity [128]. Moreover, HCO_3_^−^ is a primary constituent of produced waters from coalbed natural gas development and effluents from mountaintop mines and valley fills [304,305,322].

Although the relative acute toxicity of Mg^2+^ was ranked as similar to that of HCO_3_^−^ [128], much of the available literature emphasizes the effects of a lack of Mg^2+^ in ambient waters or diet, although at least 1 study found toxic effects from the highest tested [Mg^2+^]. The bulk of toxicological research focuses on whether Mg^2+^ contributes to the ameliorative effects of water hardness on metal toxicity. Some fish appear to need Mg^2+^ in their diet, but there is also evidence for uptake of Mg^2+^ by ionocytes on the gill or other epithelial membranes from ambient waters. However, no studies have been found on the identity or nature of Mg^2+^ transporters on these epithelia, although there are some data on transporters in the epithelia of the intestine or in the renal system. Therefore, additional research is needed to understand the transport of Mg^2+^ in freshwater animals and fully understand its role in ionoregulation, especially in combination with other ions.

The literature is clear that Na^+^ and Cl^−^ generally dominate osmoregulation of freshwater animals and of most living organisms in general, although all inorganic ions and organic osmolytes contribute to intracellular and extracellular osmolarity. Increasing water [Na^+^] or [Cl^−^], such as occurs along the gradient between freshwater and estuarine or marine waters, is generally accompanied by increasing blood or hemolymph [Na^+^] or [Cl^−^] as the animals maintain the hypertonicity of their extracellular fluids. Diadromous fish, such as salmon or eels, adapt to the increasing salinity by inactivating ionocytes with freshwater transporter arrangements designed for ion uptake and activating ionocytes with saltwater transporter arrangements designed for ion excretion [103,323]. Invertebrates generally iono-conform, and most fish hyporegulate in greater salinity waters. Many freshwater species often hyper-regulate with increasing salinity until the animals are no longer able to maintain blood or hemolymph [Na^+^] or [Cl^−^] greater than in the water, and the animals become isotonic to the water. It is then that mortality occurs in stenohaline species, and this is why Na^+^ and Cl^−^ toxicity occurs at relatively greater concentrations compared with some other major ions. However, because Na^+^ and Cl^−^ are exchanged for H^+^ and HCO_3_^−^, respectively, the relative internal concentrations of these ions can reflect alterations of acid—base balance, which is often quantified as the difference between the equivalents of strongly disassociated cations (i.e., Na^+^, K^+^, Ca^2+^, and Mg^2+^) and of strongly disassociated anions (i.e., Cl^−^ and SO_4_^2−^)—the strong ion difference [60,295] that may be the result of increases in water salinity.

There has been little research on the physiology of SO4^2−^ in freshwater animals. Most available data suggest that SO_4_^2−^ is at most slowly permeable to gill epithelial ionocytes, and this appears to explain the ion’s relatively low toxicity to daphnids or fathead minnows [128]. However, SO_4_^2−^ transporters have been identified in skin ionocytes of European toads and active transport appears to occur in freshwater mussels. To fully understand SO_4_^2−^ transport or the lack thereof in freshwater animals, research is needed to establish whether SO_4_^2−^ transporters are present in gills or other external epithelial membranes or in the gastrointestinal system of these animals, and if present, to elucidate the roles of these transporters.

A statistically significant adverse effect could not be attributed to Ca^2+^ in toxicological studies of daphnids and fathead minnows [128]. The physiological literature suggests that the Ca^2+^ transporters help exert a strong homeostatic effect on dissolved Ca^2+^ in both the extracellular and intracellular fluids. Although there can be significant requirements for Ca^2+^, much of this ion is stored either bound to proteins or phospholipids within cells or as inorganic salts in the endo- or exoskeleton as well as in gastroliths in Crustacea and concretions in Mollusca. As a result, intracellular free Ca^2+^, the Ca species with the most potential for adverse effects, is maintained in the 0.1 pM to 1 pM range.

The availability of data on freshwater ionoregulation physiology is very uneven across the 4 taxonomic groups. The literature on teleost fish is the most extensive, and that for Crustacea is the second most extensive. Research on freshwater Mollusca and particularly bivalves is relatively limited; much of it was published before 2000, and does not include the genomic data that have become common in the fish literature.

The group most lacking in data is the aquatic insects, particularly the Ephemeroptera, Plecoptera, and Trichoptera, whose assemblages are important to the bioassessment of streams and rivers. Because aquatic insects migrated from terrestrial to freshwater environments, an unexplored potential exists that the mechanisms of ionoregulation differ from those in other freshwater animals that migrated from estuarine or marine to freshwater environments. Moreover, the separate migration of different orders of insects to freshwater suggests that there could be alternate evolutionary solutions to the problems of ionoregulation in freshwaters. This has been shown to be true of gas exchange in aquatic insects [16].

As evidenced by the literature reviewed, the 2 disciplines that meet at the nexus of freshwater ionoregulation and osmoregulation are physiology and toxicology. However, the questions usually asked by these 2 disciplines can be very different. The toxicologists want to know how much of an ion causes adverse effects to individuals and how that may be related to effects at higher levels of biological organization, such as populations and often communities. The physiologists want to know the mechanisms that allow maintenance of internal homeostasis for these ions, which have various functions in cells and the blood or hemolymph, particularly when these mechanisms are related to natural ionic gradients, such as from freshwater to saltwater or from soft to hard water. Both toxicologists and physiologists investigate when maintenance of internal homeostasis fails, such as in metal toxicity, where adverse effects occur after metal ions are inadvertently transported across apical ionocyte membranes by the ECaC or apical Na^+^-channel because the specific metal ions have a similar charge and atomic radius to Ca^2+^ and Na^+^, respectively. This is part of the physiological basis of the biotic ligand model, although this often has not been made clear in the toxicological literature.

Given the differing questions of freshwater toxicology and physiology, there are striking differences in the model organisms used by the 2 disciplines. Until recently, many common techniques in physiology either measured directly or sampled and measured ion or radioisotope concentrations in intracellular or extracellular fluids. A microprobe, cannula, or hypodermic needle, for example, would have been placed into the animal without disturbing normal functions. This often required larger, often adult species, such as rainbow trout or crayfish. In freshwater toxicology, there has been an advantage to using species that can be cultured in significant numbers and used when needed at a specific life stage (i.e., larval or neonate). As a result, daphnids and fathead minnows dominate the freshwater toxicological literature. The increasing use of zebrafish represents a change that is tied to the use of genomic data. The increasing use of molecular methods is slowly altering these size and natural history limitations on species used in toxicological studies.

Lack of understanding of ionoregulation in aquatic insects occurs, in part, because they do not meet the requirements for either physiology or toxicological studies. Aquatic insects are usually smaller than even crayfish. They also have complex life cycles in which the adult often does not live in water and is short-lived. Moreover, other large life stages, such as the penultimate and final instars, only persist for a short period and the entire life cycle is usually univoltine, although there are species with multivoltine life cycles that may persist for a month at warmer temperatures. Use of different model species, such as 1 of the Ephemeroptera, Plecoptera, or Trichoptera, may be required to close some gaps in our knowledge. A species of parthenogenic baetid mayfly, *Neocloeon triangulifer*, is currently being investigated for use in aquatic toxicology [324,325].

Finally, freshwater toxicology can gain a better understanding of the toxic mechanisms of major ions that may be elevated by anthropogenic sources from what is known about the physiology of ion transport. Although there is some interaction among those ions that share transporters, such as H^+^ and Na^+^ or HCO_3_^−^ and Cl^−^, it is not the type of interaction that has been assumed based on the example of divalent metals and hardness. For ions that have some biochemical or physiological role in animals, including nutrient metals, such as Cu and Zn, there are specific transporter proteins involved in their uptake across apical and basolateral membranes of ionocytes on the gills or other external epithelial surfaces or in the gastrointestinal systems of freshwater animals at normal concentrations. However, some ions have been incompletely studied, and we currently do not know the identity and characteristics of some of these transporters. This is particularly true of Mg^2+^ and SO_4_^2−^ but is also true of an apical K^+^ transporter. There are many more gaps in our knowledge of freshwater invertebrates, particularly aquatic insects. Finally, even in comparative physiological literature reviews, comparisons across phyla and classes of freshwater animals similar to what have been made in the present review do not exist.

## CONCLUSIONS

Where there are physiological data to compare, there are functional similarities among ion transport processes, particularly for freshwater fish, crustaceans, and molluscs. The similarities are at least partially the result of evolutionary conservatism in ion transporter proteins. However, there are differences, such as the electroneutral NHE in teleost fish, the electrogenic H2Na(Ca)E in crustaceans, and a pharmacologically different NHE in aquatic insects. Also, there is some evidence that the relative affinity of the ECaC for Ca^2+^ or the apical Na^+^-channel for Na^+^ differs between aquatic insects and fish, crustaceans, or molluscs compared with the metals that also can be transported by these channels.

Increased concentrations of ions in the water most directly affect ionoregulation or osmoregulation and cause adverse effects when they change osmotic or electrochemical gradients or otherwise interact with other ions. Increased external aqueous [H^+^] or [HCO_3_^−^] change the electrochemical gradients across the apical epithelial membrane whereby the movement of these ions down their electrochemical gradient energizes uptake of Na^+^ and Cl^−^, respectively. Inhibition of CA also affects this concentration gradient by reducing the [H^+^] and [HCO_3_^−^] within the cell. Anything affecting these 2 ion transport processes also affects the excretion of H^+^ or HCO_3_^−^ required for acid—base regulation. Along with K^+^, Na^+^ and Cl^−^ appear to be particularly important for osmoregulation, with K^+^ being important in the movement of Na^+^ from intracellular to extracellular fluids and for volume regulation. A large enough increase in the water [Na^+^] and [Cl^−^] or [total ions] can change the osmotic gradients maintained by freshwater animals. An ion that is not the primary ion transported, but that can pass through the transporter, may compete for attachment sites on the transporter and thereby inhibit uptake of the primary ion. This occurs in the ECaC with metals and the anion exchanger with NO2^−^. As a result, research to address the effects of increased salinity in freshwaters associated with different sources and compositions of ions needs to consider the interactions among individual ions along with the effect of total ion concentrations on the movement of water or ions across epithelial membranes.

## Supplementary Material

Supplement1

## Figures and Tables

**Table 1.** Dominant ions associated with different anthropogenic sources of salts

Source	Dominant ions	Reference
Use of salt to melt ice and snow	Na^+^, Cl−, Ca^2+^, Mg^2+^	Forman and Alexander [326], Kaushal et al. [327],
Weathering of concrete in urban drainage systems	K^+^, Ca^2+^, HCO_3_^−^	Kelting et al. [328] Davies et al. [329], [330]
Produced water from traditional oil and gas production	Na^+^, Cl^−^, SO_4_^2−^	Boelter et al. [331], Veil et al. [332]
Produced water from coalbed methane production	Na^+^, HCO_3_^−^, Cl^−^	Jackson and Reddy [333], Brinck et al. [334],
Flowback and produced water from shale gas production (i.e., hydraulic fracturing)	Na^+^, Cl^−^, Mg^2+^, Ca^2+^, Br^−^	Dahm et al. [335] Entrekin et al. [336], Haluszczak et al. [337]
Runoff and effluents from traditional coal mining	SO_4_^2−^, Na^+^, Cl^−^, Ca^2+^, Mg^2+^, K^+^	Kennedy et al. [338], Hopkins et al. [339]
Runoff from valley fills associated with mountaintop mining	Ca^2+^, Mg^2+^, HCO_3_^−^, SO_4_^2−^	Griffith et al. [322]
Coal combustion residue effluents	Ca^2+^, Mg^2+^, Cl^−^, SO_4_^2−^	Ruhl et al. [340]
Irrigation runoff	Na^+^, Mg^2+^, Cl^−^, F^−^, SO_4_^2−^	Leland et al. [341], Scanlon et al. [342]
Anthropogenic increases in geochemical weathering	Ca^2+^, HCO_3_^−^, SO_4_^2−^	Raymond and Oh [343], Kaushal et al. [344]
Industrial sources	Na^+^, Cl^−^	Echols et al. [345]
Wastewater treatment plants	Na^+^, Cl^−^, K^+^, SO_4_^2−^	Andersen et al. [346], Hur et al. [347]

**Table 2.** List of ion transporters identified by the present review in the physiological literature on freshwater fish, Crustacea, aquatic insects, and Mollusca

Transporter	Acronym	Function	Gene family
V-type H^+^-ATPase	VHA	Transports H^+^ across apical or basolateral membranes	ATPase H^+^-transporting subunits
Apical Na^+^-channel	—	Transports Na^+^ across usually apical membranes	?
Acid sensing ion channel	ASIC	Possibly transports Na^+^ across usually apical membranes	Acid sensing ion channel subunits
Na^+^/H^+^-exchanger	NHE	Exchanges Na^+^ for H^+^ across apical membranes	Solute carrier family 9
P-type Na^+^/K^+^-ATPase	NKA	Exchanges Na^+^ and K^+^ across basolateral membranes	ATPase Na^+^/K^+^-transporting subunits
K^+^-channel	KC	Transports K^+^ across basolateral membranes	K^+^ voltage-gated channel
Na^+^/HCO_3_^−^-cotransporter	NBC	Transports Na^+^ and HCO_3_^−^ across basolateral membranes	Solute carrier family 4
Cl^−^/HCO_3_^−^-exchanger	AE	Exchanges Cl^−^ for HCO_3_^−^ across apical membranes	Solute carrier family 4
Epithelial Ca^2+^-channel	ECaC	Transports Ca^2+^ across apical membranes	Transient receptor potential cation channel
Ca^2+^-ATPase	PMCA	Transports Ca^2+^ across basolateral membranes	ATPase Ca^2+^ transporting
Cl^−^-channel	—	Transports Cl^−^ across basolateral membranes	?
Carbonic anhydrase	CA	Catalyzes the hydrolysis of CO_2_ to form H^+^ and HCO_3_^−^	Carbonic anhydrase
Na^+^/Ca^2+^-exchanger	NCX	Exchanges Na^+^ for Ca^2+^ across basolateral membranes	Solute carrier family 8
Na^+^/Cl^−^-cotransporter	NCC	Transports Na^+^ and Cl^−^ across apical membranes	Solute carrier family 12
Na^+^/K^+^/Cl^−^-cotransporter	NKCC	Transports Na^+^, K^+^ and Cl^−^ across apical membranes	Solute carrier family 12
H^+^/2Na^+^(or Ca^2+^)-exchanger	H2Na(Ca)E	Exchanges 2Na^+^ or Ca^2+^ for H^+^ across apical membranes	Solute carrier family 9
Anion/SO_4_^2−^-antiporter	—	Exchanges another anion for SO_4_^2−^across apical membranes	?

ATPase = adenosinetriphosphatase; V-type = vacuolar-type; P-type = Purkinje cells-type; ? = not sufficiently characterized to place in a gene family.

**Table 3.** Results of bioassays with Crustacea comparing NaHCO_3_ and NaCl

Species	Endpoint	Effect	Concentration (mM)	Salt	Conductivity (μS cm ^−1^)	Reference
*Ceriodaphnia dubia*	EC10	Reproduction	19.9 ± 1.5	NaHCO_3_	1900	Lopez Vera et al. [348]
*C. dubia*	EC50	Reproduction	24.1 ± 0.3	NaHCO_3_		
*C. dubia*	EC10	Reproduction	ND	NaCl	2800	
*Daphnia magna*	48-h LC50	Mortality	15.1 ± 2.2	NaHCO_3_		Hoke et al. [349]
*D. magna*	48-h LC50	Mortality	81.3 ± 6.2	NaCl		
*C. dubia*	48-h LC50	Mortality	12.8 ± 1.5	NaHCO_3_		
*C. dubia*	48-h LC50	Mortality	13.5 ± 2.2	NaCl		
*C. dubia*	48-h LC50	Mortality	11.5	NaHCO_3_		Harper et al. [304]
*C. dubia*	EC20	Reproduction	4.5	NaHCO_3_		Farag and Harper [305]
*Paratya australiensis*	10-d LC10	Mortality	10.1 ± 1.4	NaHCO_3_		Lopez Vera et al. [348]
*P. australiensis*	10-d LC50	Mortality	15.2 ± 2.5	NaHCO_3_		

C10=10% effects concentration; EC20=20% effects concentration; LC10=10% lethal concentration; LC50=median lethal concentration; ND=not determined.
