# Supplementary material for: TOXICOLOGICAL PERSPECTIVE ON THE OSMOREGULATION AND IONOREGULATION PHYSIOLOGY OF MAJOR IONS BY FRESHWATER ANIMALS: TELEOST FISH, CRUSTACEA, AQUATIC INSECTS, AND MOLLUSCA
Source: Environ Toxicol Chem. Author manuscript; Available in PMC 2018 Aug 29. (PMC6114146; doi:10.1002/etc.3676)
Supplement: Supplement1 [file NIHMS1503989-supplement-Supplement1.docx]

| Aspect of Transport | Evidence |
| --- | --- |
| Aquaporins | In European eel (*Anguilla anguilla*), a homolog of mammalian aquaporin 3 (AQP‑3) occurs on the apical membrane of gill epithelial cells [1, 2]. AQP‑3 mRNA expression in the gills of adult silver eels (i.e., SW adult stage) in FW was 3 times that in yellow eels (i.e., FW adult stage). Moreover, in silver and yellow eels acclimated to SW for 3 wks, gill AQP‑3 mRNA expression was reduced 97 and 76%, respectively, of AQP‑3 mRNA expression in eels acclimated to FW. In Atlantic salmon (*Salmo salar*), 3 aquaporin isoforms were found in the gills [3]. Expression of 2 aquaporins, AQP‑1a and AQP‑3, was greater in gills of FW‑acclimated Atlantic salmon, while expression of the third, AQP‑1b, was greater in gills of SW‑acclimated Atlantic salmon, suggesting that the roles of these aquaporin isoforms in water transport differ. |
| Compromise of septate junctions by high ion concentrations in FW bivalves | When *Toxolasma texasiense* or *Corbicula fluminea* were exposed to AFW that was made hyperosmotic by addition of 100 mmol mannitol or glucose to avoid the confounding effects of elevated individual inorganic ion concentrations, some EC fluid ion concentrations increased initially (upto 36 h in *C. fluminea* and only up to 8 h in *T. texasiense*), probably as a result of water movement out of the animals along the altered osmotic gradient, because there were no changes in Na^+^ or Cl^−^ flux. After this initial period, most IC fluid ion concentrations decreased, with K^+^ decreasing below detection limits, and sugar concentrations increased despite the absence of sugar transporters. When lanthanum (as LaCl_3_, which forms an insoluble precipitate, La_2_(CO_3_)_3_, when it is exposed to endogenous HCO_3_^−^) was added to the hyperosmotic PW as an electron‑dense diffusion tracer, La precipitates were observed in the IC space between the epithelial gill cells [4]. |
| Tolerance of a stonefly nymph to increasing salinity | Many stenohaline FW species can tolerate elevated [Na^+^] and [Cl^−^] that are at most isotonic with their hemolymph. Nymphs of the stonefly *Paragnetina media* were tolerant (100% survival) of salinities ranging from DW up to 163 mM NaCl, which was approximately isotonic to their hemolymph [5]. However, at salinities of 188 and 205 mM NaCl, which were hypertonic to the nymphs’ hemolymph, 15 and 80% of the nymphs died by 72 h, respectively. |
| Interspecific variation in sensitivity to increased salinity among centrarchids | While the centrarchids, spotted sunfish (*Lepomis punctatus*) and spotted bass (*Micropterus punctulatus*), took more than 12 h to acclimate to increased salinity of up to 8‰, a third species, redear sunfish (*Lepomis microlophus*), acclimated in less than 1 h [6]. The acclimation times for spotted sunfish and spotted bass were sufficiently long that these two species are unlikely to adapt to habitats where salinity varies with the diurnal tidal cycle [6]. |
| Interspecific variation in sensitivity to increased salinity among *Daphnia* spp. | In *Daphnia*, a 48‑h EC50 for immobilization (49.6 mM NaCl for *D. longispina* vs. 101 mM NaCl for *D. magna*) and a 21‑d (chronic) EC50 for life history parameters (37.6 mM NaCl L^−1^ for *D. longispina* vs. 85.6 mM NaCl for *D. magna*) for *D. longispina* were nearly one-half those for *D. magna* [7]. |

| Aspect of Transport | Evidence |
| --- | --- |
| Variation in sensitivity to increased salinity among clonal groups of the *Daphnia pulex* complex | Even within some species that are not necessarily euryhaline, there can be great variability in tolerance to salinity. In a survey of FW rock bluff and tundra ponds along a salinity gradient related to their proximity to the shore of Hudson Bay, Canada, as many as 7 parthenogenetic clonal groups of the *D. pulex* complex inhabited ponds ranging in specific conductivity from 236 to 1,162 μS cm^−1^ up to 1,204 to 16,955 μS cm^−1^ [8]. Similarly, there was great variability in the fitness of clones from a brackish water lake and a FW pond exposed to different salinities [9]. |
| Ability of FW molluscs to use organic osmolytes to increase hemolymph osmolarity | Comparing two FW molluscs, the gastropod *Pomacea bridgesi* and unionid *Lampsilis teres* [10] were gradually acclimated to media ranging from FW to dilute SW with an osmolality of 400 mOsm (i.e., 200 mM NaCl) [10]. There was 100% survival in *L. teres* and 80−100% survival in *P. bridgesi* up to 200 mOsm (100 mM NaCl); survival decreased to 80 and 0%, respectively, in 400 mOsm. *P. bridgesi* remained hyperosmotic and maintained greater hemolymph [Na^+^], [Cl^−^], [K^+^], and [Ca^2+^] than the water up to 200 mOsm, whereas *L. teres* became isosmotic to the water at 50 mOsm (i.e., 25 mM NaCl) and maintained hemolymph concentrations of individual ions that were similar or slightly greater than the water. While amino acids that are osmolytes, such as alanine, glycine, and β‑alanine, increased in the gills of both molluscs, the increase was greater in *L. teres* than in *P. bridgesi,* which has less capacity to increase these amino acids in response to increasing salinity. In another unionid, *Ligumia subrostrata*, these amino acids constitute only 1% of hemolymph solutes but 11% of the solutes in IC fluids and are important in regulating cell volume [11]. |
| Shifts in the energy budget of juvenile common carp at near isotonic conditions | In stenohaline juvenile common carp, exposure to Na^+^ and Cl^−^ at near isotonic concentrations (i.e., 171 mM NaCl) for 28 d resulted in increased blood osmolality and ion concentrations, which remained slightly hypertonic for total ions and Cl^−^ and slightly hypotonic for Na^+^ [12]. Although, the strongest effect was reduced appetite (i.e., food ingestion decreased initially by 30% and later by 70%), juvenile growth rates were reduced to zero, which was a greater reduction than in reference fish placed in FW whose food ration was decreased to match the ingestion rates of the juveniles in isotonic water. Moreover, respiration rates were increased and glycogen stores in the liver were reduced in the juveniles reared in the isotonic water, suggesting a reallocation of energy to osmoregulation. |
| Mechanism for reducing energy expenditures for ion uptake by Amazonian oscar exposed to hypoxia | Amazonian oscar (*Astronotus ocellatus*), a fish of low‑oxygen black waters, is able to reduce efflux of Na^+^ and also K^+^ when exposed to hypoxia [13]. The fish reduce transcellular permeability by closing channels in the ionocytes, in part by covering apical crypts in pavement cells, thereby reducing energy expenditure for ion uptake at a time when O_2_ is not available for aerobic respiration. |
| AQP, aquaporin; AFW, artificial freshwater; DW, distilled water; EC, extracellular; EC50, median effect concentration; FW, freshwater; IC, intracellular; PW, pond water; SW, saltwater. | |

| Aspect of Transport | Evidence |
| --- | --- |
| Expression of claudin gene in fish in FW and SW | In green spotted pufferfish (*Tetraodon nigroviridis*), 2 of 4 claudin‑3 genes, all 4 claudin‑8 genes, and all 4 claudin‑27 genes found in this species were expressed in the gills [14, 15]. Expression of claudin mRNAs, *Tncldn3a*, *Tncldn3c,* and *Tncldn8d*, was greater in FW compared to SW, while expression of *Tncldn27a* and *Tncldn27c* mRNA was less in FW. However, expression of the 5 genes was not statistically different between FW and SW. Similar differences in expression of gill claudin isoform mRNAs in FW and SW were observed in Atlantic salmon [16] and tilapia *(Oreochromis mossambicus)* [17]. |
| Expression of occludin and claudins in goldfish (*Carassius auratus*) in ion-poor water | In goldfish, acute exposure to ion‑poor water after acclimation to FW increased occludin expression by 10 to 20% only during the first 3 h, while longer acclimation to low-ion water (14 to 28 d) increased occludin expression by 400 to 500% compared to exposure to FW only [18]. Transcript abundance also increased for 6 of 8 claudins, and tight junction depth increased in goldfish acclimated to low-ion water [19] suggesting that occludin and most claudins play roles in decreasing ion loss across the tight junctions. |
| Role of cortisol in expression of occludin and claudins | In cultured rainbow trout (*Oncorhynchus mykiss*) gill epithelia, the glucocorticoid hormone cortisol increased mRNA expression of occludin and most claudins, while transepithelial resistance increased and paracellular permeability decreased [20, 21]. In cultured spotted green pufferfish gill epithelia, cortisol had variable effects on mRNA expression of various claudin isoforms [22], and the heterogeneous changes in claudin abundances appear to differentially affect paracellular permeability. Cultured goldfish gill epithelia showed a much smaller response of occludin, claudins, and ZO‑1 mRNA to cortisol than did rainbow trout [23], which may explain goldfish stenohalinity, although the cultured gill epithelia included only pavement cells, not ionocytes. |
| FW, freshwater; SW, saltwater; Tncldn, *Tetraodon nigroviridis* claudin; ZO, zona occludins. | |

| Subsection | Aspect of Transport | Evidence |
| --- | --- | --- |
| Teleost fish | VHA with apical Na^+^‑channel | External treatments with bafilomycin, which inhibits VHA, have reduced Na^+^ uptake as much as 90% in juvenile tilapia and common carp (*Cyprinus carpio*) [24]. |
|  | NHE | Parks et al. [25] argued against an operable NHE, particularly in low-ionic FW, for thermodynamic reasons. However, Parks et al. [26] provided evidence for a Cl^−^-dependent NHE in rainbow trout. |
|  | NHE and apical Na^+^‑channel | Parks et al. [27] localized NHE to both α-type and β-type ionocytes, while also localizing the apical Na^+^-channel to α-type ionocytes in rainbow trout. |
|  | NHE and VHA with apical Na^+^-channel | In FW-reared tilapia, both types of Na^+^ transport mechanisms were identified [28]. |
|  | VHA | The β-subunit of VHA was cloned from both the gills and kidney of rainbow trout [29]. |
|  | NKA | In isolated tilapia gill MR ionocytes exposed to ouabain or copper (Cu^2+^), both of which inhibit NKA activity, intracellular [Na^+^] increased [30]. Addition of a membrane-permeant Cu^2+^ chelator to the Cu^2+^ treatment decreased intracellular [Na^+^] because the chelator reversed the inhibition of NKA activity. |
|  | NHE metabolon | Infolding the basolateral membrane brings that membrane near the apical membrane and allows the NKA to create areas of low [Na^+^] near the NHE [31]. Production of H^+^ by cytosolic CA also creates areas of greater [H^+^] close to the NHE. Finally, mucus on external gill surfaces may concentrate Na^+^ in the boundary layer outside the apical membrane [32]. |
|  | Regulation of intracellular [Na^+^] | Whatever the transporters, though, Na^+^ uptake rate across the apical membrane of ionocytes is regulated to match the extrusion rate of Na^+^ across the basolateral membrane to maintain intracellular [Na^+^] and cell volume [33]. |
|  | Uptake of Na^+^ from food | When fed a meal that provided 6.4 mmol Na^+^ kg^−1^ fish, rainbow trout absorbed ≈90% of the Na^+^ content of ingested food in their stomach but then appeared to excrete a similar amount of Na^+^ into the anterior intestine, resulting in net absorption of ≈9% [34]. Another study of rainbow trout observed similar absorption of dietary Na^+^ loads of 0.5 to 70 mmol kg^−1^ fish, but did not measure [Na^+^] in the intestine [35]. Gill epithelial Na^+^ uptake decreased across the range of dietary Na^+^ loads, and branchial Na^+^ efflux increased particularly at greater dietary Na^+^ loads (i.e., >18 mmol kg^−1^ fish), with little change in excretion via the kidneys [35]. Moreover, blood plasma [Na^+^] did not increase substantially in either study when dietary Na^+^ loads were similar. However, rainbow |
| Subsection | Aspect of Transport | Evidence |
| Teleost fish (continued) | Uptake of Na^+^ from food  (continued) | trout appear to use Na^+^ from their diet to replace losses associated with exposure to low pH [36]. |
|  | CA | CA plays a role in Na^+^ transport by catalyzing production of H^+^ from the hydrolysis of CO_2_. A CA isoform identified in rainbow trout gills and involved in acid‑base regulation [37], tCAc, is collocated on the apical membrane with NKA on the basolateral membrane of some gill ionocytes, although both were located separately in other ionocytes [38]. When respiratory acidosis was imposed on the trout by elevating the water *P*_CO2_, tCAc levels and net H^+^ excretion increased, while net H^+^ excretion was inhibited by treatment with acetazolamide, which inhibits CA. |
|  | Role of two apical Na^+^ transport systems in ionoregulation vs. acid‑base regulation | Sodium green, a Na^+^‑dependent fluorescent reagent, was used to collocate Na^+^ accumulation in a mitochondria-rich ionocyte subpopulation that exhibited high VHA levels but only moderate NKA levels [39]. To elucidate the Na^+^ uptake mechanisms of these cells, larval zebrafish (*Danio* rerio) were exposed to the ion transporter inhibitors, EIPA (inhibits NHE), amiloride (inhibits apical Na^+^-channel at low concentration, apical Na^+^-channel and NHE at high concentration), bafilomycin (inhibits VHA), metolazone (inhibits CA), ouabain (inhibits NKA) and ethoxzolamide (inhibits CA) [39]. Na^+^ uptake was inhibited by EIPA, bafilomycin, ethoxzolamide, and higher concentrations of amiloride, suggesting that NHE and not the apical Na^+^-channel was involved in the Na^+^ uptake. As CA supplies the H^+^ needed to exchange for Na^+^, its inhibition would affect the NHE, while the inhibition of VHA suggests that the electrochemical gradient created by VHA may help energize the NHE [39, 40], particularly in soft, very low [Na^+^] (35 μM) waters [41]. |
|  | Role of two apical Na^+^ transport systems in low vs. higher [Na^+^] waters | In soft, very low [Na^+^] waters, both Na^+^ uptake affinity and capacity were at least twice that in hard, higher [Na^+^] (1480 μM) waters. Na^+^ uptake in soft water was inhibited by a higher concentration of the VHA inhibitor bafilomycin (1 μM), but not in hard water [41]. At a lower concentration of bafilomycin (0.05 μM), Na^+^ uptake increased in hard water but not soft water, providing evidence for both transport mechanisms (i.e., VHA and apical Na^+^-channel in soft water and NHE in hard water). In such waters, both Na^+^ uptake affinity and capacity were at least twice that in hard, higher [Na^+^] (1480 μM) waters. |

| Subsection | Aspect of Transport | Evidence |
| --- | --- | --- |

| Teleost fish (continued) | NHE3 under acidic conditions | Immunohistochemical methods have identified NHE3-type NHE in the gills of rainbow trout [42] and zebrafish. In zebrafish, at least 8 NHE isoforms were identified [43]. Two isoforms, zNHE2 and zNHE3b, were expressed predominately in the gills, and zNHE3b was relevant to acidic conditions. The isoform zNHE3b was not located on MR ionocytes with NKA but near the opening of the apical membrane in H^+^-ATPase-rich ionocytes along with concanavalin A, an apical marker [44]. When the adult zebrafish were acclimatized to acidic water (pH 4.0−4.1), zNHE3b mRNA decreased to 94% of the control (pH 6.7−6.9), while *zatp6v0c* (subunit c of VHA) mRNA increased to 138% of the control. In zebrafish acclimatized to low [Na^+^] FW (0.04−0.05 mM), zNHE3b mRNA was upregulated by 157% compared with the high [Na^+^] control (10.0−10.5 mM), while *zatp6v0c* mRNA was downregulated to 58% of the control [43], showing that zNHE3b is involved in Na^+^ uptake and not acid regulation, while the converse is true of *zatp6v0c*. Similarly, zNHE3b was upregulated and *zatp6v1a* (subunit a of VHA) was downregulated in low [Na^+^] water (0.005 mM), and the reverse occurred in higher [Na^+^] water (0.5 m) [45]. |
| --- | --- | --- |
|  | NHE3 under acidic conditions and a NHE3/Rhcg1 metabolon | The role of zNHE3b in Na^+^ uptake, particularly under acidic conditions (pH 3.9−4.0), at least in larval zebrafish, may be tied to a metabolon that also includes the excretion of NH_3_ by the Rhesus protein, Rhcg1 [46]. Larvae exposed to acidified dechlorinated TW (pH 3.9−4.0) for 4 to 5 d after fertilization were compared with control fish exposed to unacidified water (pH 7.3−7.5). Pointing out that zebrafish along with the Osorezan dace (*Tribolodon hakonensis*) are among the few FW teleosts known to increase Na^+^ uptake in acidic water, the authors compared both NH_3_ excretion and Na^+^ uptake in control and test fish. In some series, test fish were acutely exposed to water (pH 7.3−7.5) to separate the effect of the rearing pH from the effect of the testing pH. In two series, the water was manipulated to inhibit NH_3_ excretion by maintaining high external [NH_3_/NH_4_^+^] and highly buffered conditions. EIPA, a NHE inhibitor, was also added to the unacidified water or the buffered water, and the VHA inhibitor bafilomycin A_1_ was added to the unacidified water in 3 series. A morpholino oligonucleotide designed to knockdown zebrafish Rhcg1 or a sham were injected into embryos used in two series. In these experiments, Na^+^ uptake was always at least two to 4 times greater in larvae acclimated to pH 3.9−4.0 than to pH 7.3−7.5. Except for the treatment with high external NH_3_/NH_4_^+^, which affected Na^+^ uptake in both control and acid‑acclimated larvae, the treatments reduced NH_3_ excretion in both control and acid‑acclimated larvae but reduced Na^+^ uptake only in the acid‑acclimated zebrafish larvae. |

| Subsection | Aspect of Transport | Evidence |
| --- | --- | --- |
| Crustacea | Electrogenic H^+^/2Na^+^(or Ca^2+^)-exchanger [H2Na(Ca)E] | This exchanger is electrogenic rather than electroneutral because it exchanges 2 Na^+^ for a 1 H^+^, which may allow the thermodynamic capacity to move H^+^ ions against a greater concentration gradient [47]. |
|  | Competitive inhibition of Na^+^ uptake by elevated [Ca^2+^] | Competitive inhibition of Na^+^ uptake by elevated external [Ca^2+^] has been observed in the crayfish *Austropotamobius pallipes* [48]. |
|  | Lack of homology with mammalian NHE | Use of a monoclonal antibody from mice putatively for the mammalian NHE suggests the crustacean H2Na(Ca)E differs structurally from the vertebrate NHE [49]. |
|  | H2Na(Ca)E transport in *Daphnia* | In *Daphnia* this electrogenic H2Na(Ca)E transports 1 Na^+^ instead of 2 Na^+^ at low [Na^+^], following Michaelis‑Menten kinetics. In this model, Na^+^ and H^+^ may interact cooperatively where H^+^ binds to 1 binding site, thereby facilitating binding of Na^+^ to the second binding site and promoting increased Na^+^ flux [50]. |
|  | Noncompetitive inhibition of Na^+^ uptake by humic substances | Humic substances noncompetitively affect Na^+^ uptake in Cladocera by absorbing to the gill epithelial membranes where they change membrane permeability. This increases *J*_max_ but does not necessarily alter the *K*_m_ for Na^+^ [51]. |
|  | Na^+^ uptake during molt | During molt in FW, Crustacea absorb water and increase body volume, resulting in dilution of ions in the hemolymph [52, 53]. In the Australian crayfish *Cherax destructor* activities of both NKA and VHA increased 2−3 times during postmolt relative to normal intermolt periods, and increased ATPase activities were associated with increased *J*_net_ of Na^+^ [52]. These ATPase activities were similar to those in intermolt crayfish that had Na^+^ depleted by placing them in Na^+^‑free artificial TW. Therefore, these ATPases are involved in uptake of Na^+^ across the apical gill membrane, particularly through the apical Na^+^-channel but probably also through the NHE. |
|  | Reabsorption of Na^+^ by antennal gland | Renal filtration and reabsorption in the antennal gland can be several times greater than absorption through the gills probably because such ion recyling is more energy efficient [54]. |
|  | Maintenance of hypertonic hemolymph | The hypertonic hemolymph in FW crayfish is also maintained, in part, by reabsorption of Na^+^ along with Cl^−^ by the antennal gland [55], and NKA activity can be much greater in the antennal gland than in the gills [56]. |
| Aquatic insects | Pharmacological evidence of the presence of VHA and CA | Bafilomycin, a VHA inhibitor, reduced H^+^ export and Na^+^ import across the anal papillae by 55 and 41% respectively, while methazolamide, a CA inhibitor, reduced H^+^ export by 74% in *Aedes aegypti* [57]. |
| Aquatic insects (continued) | Pharmacological evidence of the presence of an apical Na^+^-channel and NHE on anal papillae | In whole *A. aegypti* larvae treated with 100 μM phenamil (Na^+^-channel inhibitor), Na^+^ import decreased and Na^+^ was secreted across the anal papillae, while 5‑(*N*,*N*‑hexamethylene)amiloride (NHE inhibitor) had no effect on Na^+^ import [57]. However, Pullikuth et al. [58] showed that amiloride and ethyl-isopropyl amiloride (other NHE inhibitors) only inhibited ^22^Na^+^ uptake by 40% at concentrations that completely inhibited mammalian NHE. This suggests that AeNHE3, the NHE localized to the basolateral epithelial membrane of the Malpighian tubule, midgut, and gastric caeca of *A. aegypti*, is insensitive to the amiloride-type inhibitors used to distinguish NHEs [58] |
|  | Cloning evidence for NHE on other epithelia | An NHE, AgNHE1, has been cloned from the alimentary canal of larval *Anophele*[59]. In *Anopheles albimanus*, an NHE has been localized to the apical membrane of nondorsal anterior rectal cells [60] that interacts with NKA on the basolateral membrane to reabsorb Na^+^ from the primary urine. |
|  | Na^+^ transport in Ephemeroptera | In Ephemeroptera, ionocytes are associated with thin areas of cuticle called porous plates located particularly on the tracheal gills [61-63]. Histochemical methods have been used to localize Na^+^ along with Cl^−^ to these cells in both *Rhithrogena semicolorata* and *Cloeon dipterum* [62]. |
|  | Na^+^ transport in Plecoptera | In Plecoptera, Kapoor [64] detected activity of NKA in the gills of *P. media* nymphs and showed the activity increased by 84% when the nymphs were moved from CW with 3.4 mM Na^+^ to a more dilute mixture of half CW and half DW. NKA activity decreased by 21% when the nymphs were placed in a hypertonic solution (188 mM Na^+^). This ion exchange was localized to ionocytes in the proximal wall of the tracheal gills that are separate from the tracheoles [65-67]. Colby [68] concluded simply that *Pteronarcys californica* actively transports Na^+^ against a concentration gradient. |
|  | Na+-homeostasis in Trichoptera | The body wall of *Limnephilus affinis*, a caddisfly known to inhabit both FW and estuarine (i.e., salt‑marsh pools) habitats in Europe, was relatively impermeable to Na^+^ and more permeable to water [69]. At lower external [Na^+^] (< 100 mM), hemolymph Na^+^ was maintained at greater concentrations (75−100 mM), while at higher external [Na^+^] (up to 400 mM), hemolymph [Na^+^] remained less than the external concentrations. Like some euryhaline mosquitos, the difference in osmolarity was equalized by liberation of unidentified organic osmolytes into the hemolymph, and the urine was hyperosmotic to both the hemolymph and to the water at greater external salt concentrations [70]. In contrast, two species that inhabit FW, *Limnephilus stigma* and *Anabolia* |
| Aquatic insects (continued) | Na^+^-homeostasis in Trichoptera (continued) | *nervosa*, maintain hemolymph [Na^+^] that is greater than external [Na^+^] (up to 220 mM), although the difference between the hemolymph and external [Na^+^] narrows, particularly above 120 mM Na^+^ [71]. These 2 species lacked the organic osmolytes in their hemolymph that were found in *L.* *affinis*, and their body walls were more permeable to Na^+^. Note that this research preceded recognition that ion uptake occurred via the chloride epithelia in Trichoptera larvae [72]. In general, aquatic insects are thought to have lower ion permeability than other aquatic organisms [73]. |
|  | Na^+^ transport in Odonata | In dragonflies (Odonata), ionocytes are found on chloride epithelia in the anal chamber [74], and Na^+^ is actively transported into the hemolymph through the rectum of *Uropetala carovei* [75] and *Aeshna cyanea* larvae [76]. NKA was located on the basolateral membrane of chloride epithelial ionocytes, because Na^+^ uptake was inhibited by ouabain, a NKA inhibitor, and a second unidentified ATPase was present [77, 78], likely VHA. Activity of both ATPases increased with decreasing ion concentrations. |
|  | Na^+^ transport in other classes. | Some air‑breathing aquatic insects, such as the beetle *Dytiscus verticalis*, do not rely on Na^+^ uptake from water but obtain Na^+^ from their food [79, 80]. Shaw [81] concluded that the megalopteran *Sialis lutaria* absorbed Na^+^, K^+^, and Cl^−^ from ingested water through the gut. |
| Mollusca | Evidence for VHA and an apical Na^+^-channel in Unionidae | There is minimal evidence for an apical Na^+^-channel energized by VHA in Unionidae [82, 83], and amiloride, an apical Na^+^-channel inhibitor at low concentrations, inhibits uptake of Na^+^ in *T. texasiense* and *L. subrostrata* [84, 85]. Moreover, the VHA inhibitor bafilomycin inhibits H^+^ excretion from the extrapallial fluid across the mantle in *Elliptio complanata*, demonstrating the role of VHA in acid excretion by the apical membrane of mantle epithelia [86]. |
|  | Lack of evidence for an apical Na^+^-channel in other taxa | Na^+^ import is not inhibited by amiloride, an apical Na^+^-channel inhibitor, in either *C. fluminea* or *Dreissena polymorpha* [87]. |
| Other Na^+^ transporters | Occurrence and localization of NBC in rainbow trout | In rainbow trout, the NBC was localized to the basolateral membrane of ionocytes with an apical Na^+^-channel and VHA on the apical membrane where its activity induced cytosol acidification [88]. |
|  | Function of NCC | The NCC is energized by movement of Na^+^ down a concentration gradient [89]. There is some question whether the concentration gradient of Na^+^ between FW and the cytosol is sufficient (i.e., |
| Other Na^+^ transporters | Function of NCC (continued) | currently no data exists on whether cytosol [Na^+^] specifically in Type II cells or NCC cells is less than FW [Na^+^]) [90, 91]. However, these ionocytes also pair NKA with the NBC on the basolateral membrane [89, 92, 93], forming a transporter combination that may maintain very low cytosol [Na^+^]. |
|  | Regulation of Na^+^ transporters along a salinity gradient | In tilapia, NHE3 and an NCC were localized to the apical membrane and NKCC1a to the basolateral membrane of gill NaR ionocytes [94]. When tilapia were exposed to 4 different salinities: undiluted SW, 1/3-diluted SW, FW, and DI water [94], the NHE was upregulated in DI water, while the NCC was increasingly upregulated in FW and DI water. Both the NHE and NCC are involved in Na^+^ uptake, while the NCC also is involved in Cl^−^ uptake. Conversely, NKCC1a was increasingly upregulated in 1/3‑diluted SW and undiluted SW and is involved in Na^+^, K^+^ and Cl^−^ excretion. In these fish, VHA expression did not differ among the different salinities, suggesting it was less involved in Na^+^ regulation. |
| Effects on Na^+^ transport by other ions | Effect of low pH on rainbow trout | In rainbow trout exposed to pH 4.2 for 4 d, a large net H^+^ influx across the gills was countered by excretion of one-third to one-half of the H^+^ by the kidney [95], and there was a large Na^+^ efflux, mostly through the gills. |
|  | Effect of low pH on Osorezan dace | In Osorezan dace, which live in very low pH conditions (pH 3.4−3.8), the NBC is paired with NKA basolaterally on an ionocyte with an apical NHE [96]. Expression of mRNA for all 3 transporters along with that for CA increased when the fish were moved from neutral to acidic conditions, suggesting this arrangement is an adaptation for export of acid units (H^+^), along with retention of base units (HCO_3_^−^) and Na^+^ in response to external acidity [93]. |
|  | Adaptations to low [Ca^2+^] and low pH in tetra | Fish tolerant of low [Ca^2+^] and low pH waters possess adaptations that reduce Na^+^ loss, maintain Na^+^ uptake or both [97, 98]. In neon tetra (*Paracheirodon innesi*) exposed to pH 3.5, Na^+^ uptake did not decrease, but only a small, temporary increase in Na^+^ efflux occurred [99]. However, when the neon tetra were exposed to pH 3.25, Na^+^ efflux increased substantially, while Na^+^ uptake was maintained. In blackskirt tetra (*Gymnocorymbus ternetzi*) exposed to pH 4.5, Na^+^ uptake increased and Na^+^ efflux increased but to a greater amount resulting in net Na^+^ loss [100]. Moreover, Na^+^ uptake was only reduced 25% by amiloride, an apical Na^+^-channel or NHE inhibitor depending on concentration, suggesting a difference in molecular structure of the Na^+^ transporter. In 3 additional tetra species also native to low pH waters, Na^+^ uptake was not inhibited by amiloride [101]. |
| Effects on Na^+^ transport by other ions (continued) | Adaptations to low [Ca^2+^] and pH in Amazonian fish | Of the Amazonian species tested, members of the Cichlidae generally had greater Na^+^ uptake at low pH and greater Na^+^ efflux, while members of the Characidae had lower Na^+^ uptake at low pH but also had lower Na^+^ efflux [102]. However, by 24 h, both Na^+^ uptake and efflux had decreased again to near their original levels resulting in a net Na^+^ flux near zero. |
|  | Adaptations to low [Ca^2+^] and pH in temperate fish | In yellow perch (*Perca flavescens*), a temperate, acid-tolerant species, *K*_m_ for uptake of Na^+^ was greater and Ca^2+^‑related paracellular permeability and Na^+^ efflux were less than in less acid‑tolerant species, such as rainbow trout [103]. In the acid-tolerant banded sunfish (*Enneacanthus obesus*), Na^+^ uptake was nearly completely inhibited at pH 4.0 or less, but Na^+^ efflux was 0 at pH 4.0 and reduced at pH 3.25 after acclimation at pH 4.0 [104]. |
|  | Dominant Na^+^ transporter in zebrafish in acidic waters | Increased Na^+^ uptake in acidic water by zebrafish changes the dominant Na^+^ transporter to zNHE3b, which is not inhibited by [H^+^] partly due to a link to NH_3_/NH_4_^+^ excretion [105]. |
|  | Role of claudins, occludins, and Ca^2+^ in low paracellular permeability and Na^+^ loss | Although as many as 14 claudin and 2 occludin isoforms increased at some point during a 300‑h experiment, Na^+^ efflux did not decrease because [Ca^2+^] was low. Ca^2+^ interacts with these claudins and occludins to maintain low paracellular permeability across the tight junctions, and paracellular permeability increases at low [Ca^2+^]. In mammalian kidney epithelial cells, Ca^2+^ is described as a critical component of tight junction formation [106, 107]. Increased Na^+^ efflux has long been observed in low [Ca^2+^] water at low or more neutral pH in other fish, such as brown trout (*Salmo trutta*), rainbow trout, Eurasian minnow (*Phoxinus phoxinus*), and tambaqui (*Colossoma macropomum*) [97, 108-110]. |
|  | Loss of Na^+^ at low pH by crayfish | Exposure of the crayfish *Orconectes propinquus* to pH 4.0 resulted in net retention of H^+^, a decrease in pH of 0.5 units, and negative net Na^+^ flux from the hemolymph relative to crayfish exposed to pH 7.5 [111]. The negative net Na^+^ flux resulted from ≈ 50% inhibition of Na^+^ uptake with little change in Na^+^ efflux, pointing to inhibition of Na^+^/H^+^ exchange across the apical epithelial membranes. |
|  | Variation in Na^+^ loss among crayfish species | Similarly, net Na^+^ uptake was inhibited at pH < 5.5 in *A. pallipes* [48], and hemolymph [Na^+^] decreased in *Astacus astacus* exposed to pH 4.0 [112]. The inhibition of Na^+^ uptake results from competitive inhibition with the counter ion H^+^, which is elevated in the water [113]. However, no differences in hemolymph [Na^+^] in adults or whole body [Na^+^] in juveniles were observed in *Cambarus robustus* exposed to pH ranging from 7.5 to 3.8, whereas in *Orconectes rusticus*, adult |
| Effects on Na^+^ transport by other ions (continued) | Variation in Na^+^ loss among crayfish species  (continued) | hemolymph [Na^+^] and juvenile whole body [Na^+^] were reduced in pH 4.0 and 3.8, respectively, compared to crayfish in pH 7.5 to 5.0 [114]. |
|  | Interaction of [Ca^2+^] and pH on Na^+^ uptake in crayfish, *C. destructor* | In *C. destructor* exposed to pH 4.5 and high [Ca^2+^] (500 μM), hemolymph [Na^+^] decreased during the first 96 h but then recovered and was similar to that in control crayfish (pH 7.1, 500 μM Ca^2+^) after 288 h [115]. In *C. destructor* exposed to lower [Ca^2+^] (50 μM), hemolymph [Na^+^] was less than that of the crayfish in high [Ca^2+^] even when exposed to pH 7.1. In crayfish exposed to pH 4.5 and low [Ca^2+^], hemolymph [Na^+^] exceeded that of those exposed to pH 7.1 and low [Ca^2+^] after 288 h, suggesting an interaction between Na^+^ and Ca^2+^ in the H2Na(Ca)E. |
|  | Variation in effects of low pH on mosquitos that inhabit phytotelmata | Among the larvae of 4 different mosquitos that inhabit phytotelmata with low ionic water, hemolymph [Na^+^] ranged from 75 to 115 mM and uptake rates ranged from 0.075 to 0.2 nmol Na^+^ mg^−1^ h^−1^ when the larvae were placed in control media (0.02 mM Na^+^, 0.03 mM K^+^, 0.03 mM Ca^2+^, and 0.05 mM Cl^−^, pH 6.0−6.5) [116]. When placed in low pH water (pH 3.5 and other ions the same as in the control water), Na^+^ uptake was reduced by 42 to 86% [116], suggesting increased external [H^+^] inhibited Na^+^ uptake. Conversely, when three of the culicids were placed in more saline water (Na^+^ and Cl^−^ both 6 mM and other ions the same as in the control water), Na^+^ uptake increased by 6 to 28 times, suggesting differences in uptake affinity and capacity for Na^+^ among the species. |
|  | Effects of low pH on net loss of Na^+^ in tundra pond aquatic insects | In a study of the effect of pH on invertebrates from tundra ponds in the Smoking Hills, Northwest Territories, Canada, *Orthocladius consobrinus* exhibited net loss of Na^+^ along with Cl^−^ at pH 3.0 and not at pH 4.5 or 8.0, and mortality increased with decreasing pH [117]. In comparison, another chironomid, *Chironomus riparius*, did not exhibit a net loss of Na^+^ or Cl^−^, and there was no relationship between pH and mortality. The caddisfly *Limnephilus pallens* exhibited net loss of Na^+^ only at pH 2.8 and not at pH 3.5, 4.5, or 8.0. |
|  | Effects of low pH on whole-body [Na^+^] in Ephemeroptera | *Stenonema femoratum* nymphs exposed to pH 3.5 water for 96 h had lower whole‑body [Na^+^] compared to nymphs exposed to pH 6.5 water [118]. A similar decrease in whole‑body [Na^+^] was observed in *Leptophlebia cupida* nymphs exposed to pH 3.5 water at 96 h and 192 h but not to pH 4.5 water compared with nymphs exposed to pH 6.5 water [119]. |

| Subsection | Aspect of Transport | Evidence |
| --- | --- | --- |
| Effects on Na^+^ transport by other ions (continued) | Effects of low pH Na^+^ balance in acid-tolerant vs. acid‑sensitive Plecoptera | A comparison of acid‑tolerant (*Amphinemura sulcicollis, Protonemura meyeri,* and *Leuctra moselyi*) and acid‑sensitive (*Perla bipunctata, Dinocras cephalotes,* and *Isoperla grammatica*) species of Plecoptera found that acid tolerance, including tolerance to elevated Al, was related to the effect on Na^+^ balance [120]. In the acid‑sensitive species, Na^+^ uptake was depressed at low pH due to competitive inhibition of Na^+^ attachment to the enzyme carrier (presumably the NHE) by H^+^. The affinity constants of the enzyme carrier for Na^+^ compared to H^+^ were greater in the acid‑tolerant species (Aff. Na^+^/Aff. H^+^ > 4), whereas in the acid‑sensitive species, the ratio was less than 1, suggesting that at low pH, these enzyme carriers in acid‑tolerant species have greater discrimination between Na^+^ and H^+^ resulting in less competitive inhibition of Na^+^ by H^+^ [120]. A similar whole-body [Na^+^] decrease was observed in *Pteronarcys dorsata* nymphs placed in pH 3.0 water [121] and in *Acroneuria carolinensis* exposed to pH 4.0 water [122]. |
|  | Effect of low pH on [Na^+^] in an acid-tolerant Odonata | In the acid‑tolerant dragonfly *Ladona julia*, whole-body [Na^+^] in nymphs exposed to pH 2.3 water (21.2 μmol Na^+^ nymph^−1^) for 96 h was not less than in those exposed to control water at pH 5.7 (21.2 μmol Na^+^ nymph^−1^) but was less in those exposed to pH 2.3 water with 1.0 mM total Al (8.2 μmol Na^+^ nymph^−1^) [123]. In a second study with the same species, hemolymph [Na^+^] in nymphs exposed to pH 2.3 water (142 mM Na^+^) for 96 h was also not less than in those exposed to control water at 5.9 (156 mM Na^+^), but again, was less than in those exposed to pH 2.3 water with 37 μM total Al (104 mM Na^+^) [124]. |
|  | Effect of low pH on hemolymph [Na^+^] in unionid mussels | As with other aquatic animals, exposure of the unionids *Anodonta anatina*, *A. cygnea*, *Unio pictorum,* and *U. tumidus* to low pH (4.0−4.5) in either soft (0.11 mM Ca^2+^) or hard (0.46 mM Ca^2+^) water resulted in decreased hemolymph [Na^+^] [125]. |
|  | Evidence that Ag^+^ and Cu^2+^ interact with the apical Na^+^-channel in rainbow trout | Uptake of either Cu^2+^ or Ag^+^, as well as Na^+^, was inhibited in rainbow trout gills by either bafilomycin (68%), a VHA inhibitor, or phenamil (39%), an apical Na^+^-channel inhibitor [126, 127]. Moreover, uptake of either Cu^2+^ or Ag^+^ was inhibited by greater external [Na^+^] just as the uptake of Na^+^ was inhibited at greater external [Cu^2+^] or [Ag^+^] [127]. This Na^+^ transporter was confirmed to be an apical Na^+^-channel in α-type MR ionocytes in rainbow trout by showing that increased Na^+^ uptake stimulated by a 0.4 unit increase in the IC pH of isolated α-type MR ionocytes was inhibited by 50 nM bafilomycin (VHA inhibitor), 10 µM phenamil (apical Na^+^-channel inhibitor), 1 or 10 µM Ag^+^, or 1 or 10 µM Cu^2+^ [128]. Uptake of Cu^2+^ from water is also inhibited by increased dietary [Na^+^] in rainbow trout because uptake activity by apical Na^+^-channel is downregulated by increased internal [Na^+^] [129]; this Cu^2+^ uptake was also reduced by |
| Effects on Na^+^ transport by other ions (continued) | Evidence that Ag^+^ and Cu^2+^ interact with the apical Na^+^-channel in rainbow trout (continued) | dietary exposure to Cu^2+^ [130]. At greater [Cu^2+^] (≥ 0.79 μM) there was also loss of Na^+^, particularly at low pH (5.0). |
|  | Effects of [Ag^+^] at sublethal concentrations are transient | However, at [Ag^+^] less than the LC50, these effects are transient, and the decreases in blood [Na^+^], Na^+^ influx, and NKA activity returned to reference levels by 15 to 20 d as the fish acclimated to the sublethal exposures [131]. |
|  | Evidence that Cu^2+^ is not related to hardness but is related to pH | Cu^2+^ uptake was not related to hardness or alkalinity, but uptake was reduced when the water pH was decreased from 7.8 to 5.0 [132]. However, the 96‑h LC50 for Cu in fathead minnow (*Pimephales promelas*) decreased as pH was decreased from 7.2 to 5.5 [133], probably because H^+^ and Cu combined to competitively inhibit Na^+^ uptake. |
|  | Acute LC50s for Ag^2+^ are related to gill surface area in daphnids | Acute LC50s for Ag^+^ was directly related to whole body Na^+^ uptake rate and inversely related to body mass in daphnids because gill surface area, the site of Na^+^ uptake, per gram of body mass decreased with increasing body mass [134]. |
|  |  |  |
|  | Effect of Pb^2+^ on whole‑body [Na^+^] in *Lymnaea. stagnalis* | In *L. stagnalis* exposed to dissolved lead (Pb^2+^) (0.20−0.53 μM) in moderately hard water (1.4 mM CaCO_3_ hardness), whole-body [Na^+^] decreased from ~32 μmol g^−1^ in the control to 10−20 μmol g^−1^ [135]. |
|  | Effect of metals on EC [Na^+^] in *A. cygnea* | Similarly, in *A. cygnea*, exposure to Cd^2+^, Cu^2+^, or Pb^2+^ decreased [Na^2+^] in the hemolymph and extrapallial fluids [136, 137]. |
|  | Effect of Cu^2+^ on hemolymph osmolality in *L. stagnalis* | Both hemolymph osmolality, which is primarily related to the [Na^+^], and net Ca^+^ influx were reduced in *L. stagnalis* exposed to 0.76 μM Cu^2+^ [138]. |
|  | Mechanisms by which divalent metals affect Na^+^ uptake | Both Cd^2+^ and Pb^2+^ are transported by the apical ECaC but inhibit the basolateral NKA by interference with dephosphorylation and cycling of the enzyme [139]. Pb^2+^ is known to inhibit CA activity, which produces H^+^ that is exchanged for Na^+^ [140]. |

| Subsection | Aspect of Transport | Evidence |
| --- | --- | --- |
| Effects on Na^+^ transport by other ions (continued) | Effect of Cd^2+^ on whole‑body [Na^+^] in *Chironomus* spp. | In *C. riparius* exposed to Cd^2+^ (0.16−8.7 mM) in moderately hard water (1.4 mM CaCO_3_ hardness), whole‑body [Na^+^] decreased from ~85 μmol g^−1^ in the control to 55−75 μmol g^−1^ [141]. In *Chironomus staegeri*, the bioaccumulation of Cd^2+^ is affected by the water [Ca^2+^] or hardness because Cd^2+^ crosses the apical epithelial membrane by the ECaC but inhibits NKA, which transports Na^+^ across the basolateral membrane [142]. |

CA, carbonic anhydrase; CW, creek water; DI, deionized; DW, distilled water; ECaC, epithelial Ca^2+^ channel; EIPA, 5-(*N*-ethyl-*N*-isopropyl)amiloride; EC, extracellular; FW, freshwater; H2Na(Ca)E, H^+^/2Na^+^(or Ca^2+^) exchanger; IC, intracellular; J_max_, maximum uptake rate; *J*_net_, net uptake rate; *K*_m_, ion concentration at which the uptake rate is half of *J*_max_; LC50, median lethal concentration; MR, mitochondria-rich; NaR, Na^+^/K^+^‑ATPase-rich; NBC, Na^+^/HCO_3_^−^-cotransporter; NCC, Na^+^/Cl^−^‑cotransporter; NHE, Na^+^/H^+^-exchanger; NKA, Na^+^/K^+^-ATPase; NKCC, Na^+^/K^+^/Cl^−^‑cotransporter; P_CO2_, partial pressure of CO_2_; SW, saltwater; tCAc, trout cytosolic carbonic anhydrase isoform; TW, tap water; VHA, vacuolar-type H^+^-ATPase

| Subsection | Aspect of Transport | Evidence |
| --- | --- | --- |
| Teleost fish | K^+^ uptake from food | Rainbow trout absorbed 90% of the K^+^ from a single experimental meal that supplied 3.3 mmol K^+^ kg^−1^ fish body mass [34]. |
| Crustacea | Active transport of K^+^ | In an experiment with isolated gills of *A. pallipes*, the electrochemical potential of K^+^ was greater than that in the perfusion medium, indicating that K^+^ is actively transported across the gill epithelium into the hemolymph [143]. |
| Aquatic insects | KHE in terrestrial *Manduca sexta* larvae | A KHE has been studied in larvae of terrestrial lepidopteran *M. sexta* [144, 145], but this exchange transports H^+^ toward the hemolymph and K^+^ toward the lumen of the anterior midgut, alkalinizing the lumen and not resulting in K^+^ uptake [146]. Moreover, a CA, AgCA9, which was particularly localized to the gastric caeca and transitional zone between the anterior and posterior midgut, produces HCO_3_^−^ that buffers the high luminal pH , particularly when removal of the H^+^ from the lumen by VHA and a KHE further deprotonate the HCO_3_^−^ to form CO_3_^2−^ [147]. |
|  | Reabsorption of K^+^ | Reabsorption of K^+^ in the lower Malpighian tubules of mosquito larvae appears to be a very efficient mechanism for retention of K^+^ [148]. |
|  | Active transport of K^+^ in Plecoptera | In a simple experiment that compared hemolymph [K^+^] of starved Plecoptera nymphs in CW and DI water, Colby [68] concluded that *P. californica* actively transported K^+^ from the water against a concentration gradient. |
| Mollusca | K^+^ and maintenance of ion balance | When *D. polymorpha* was placed in solutions containing different combinations of salts, solutions lacking K^+^ did not cause significant mortality (i.e., more than 2 of 20 individuals) in 51 d if the solutions contained Na^+^, Mg^2+^, and Cl^−^, but these individuals were unable to maintain normal ion balance and had lower [Na^+^] and [Cl^−^] in their blood [149, 150]. |
|  | Role of K^+^ in volume regulation by Unionidae | Gill filament lateral ciliated cells shrank (i.e., cell height decreased 20 to 30%) when gills of the FW mussel *T. texasiense* were superfused with a hypertonic solution containing 90 mM NaCl and did not reverse after 20 to 30 min [151]. When 2 mM KCl was also added to the hypertonic solution, the shrinkage recovered by 40−50%. *D. polymorpha* exposed to 45 mM NaCl with less than 0.05 mM K^+^ lost cell volume, while elevated [K^+^] (1 mM) resulted in excess cell volume [150]. FW mussels have reduced their IC solutes by balancing the solute concentrations in their blood. This includes organic osmolytes, leaving K^+^ practically the sole IC osmolyte for regulating cell volume and maintaining electrochemical balance [151]. This type of regulation appears to make these bivalves much more sensitive to a balance of Na^+^ and K^+^ in ambient waters to maintain cell volumes. |
| Mollusca (continued) | K^+^ transport in *Dreissena* vs. unionids | The uptake rate of K^+^ was 0.7 μmol g^−1^ dry tissue h^−1^ for *C. fluminea* and 0.4 μmol g^−1^ dry tissue h^−1^ for the unionids *T. texasiense* and *L. subrostrata* placed in PW containing about 0.06 mM K^+^ [152]. However, the transport mechanism is unclear. In *Dreissena,* uptake of K^+^ is active and exhibits saturation kinetics [150, 153]. Efflux of K^+^ was greater than uptake in *C. fluminea,* but less than uptake in the unionids [152]. Therefore, net flux was negative in *C. fluminea* and positive in the unionids, but net flux increased and was positive in both *C. fluminea* and *T. texasiense* when [K^+^] was increased to 0.13 or 0.36 mM. When Rb^+^, which can substitute for K^+^ in this type of study at the low concentrations in AFW, was substituted, *K*_m_ increased in mussels that had been placed in DI water to deplete IC and EC salt concentrations and then returned to water with K^+^ and Rb^+^, suggesting that K^+^ uptake is under endogenous control in the epithelia of these mussels [152]. Moreover, 17% of the mussels’ K^+^ was exchanged daily, suggesting high turnover of K^+^ [154]. |
| Effects on K^+^ transport by other ions in the water | Effects of low pH on fish | In brook trout (*Salvelinus fontinalis*) larvae exposed to 4 40‑h pulses of low pH, low Ca^2+^ water with or without Al over a 28‑d test, whole-body K^+^ was decreased relative to the control but did not consistently differ with decreasing treatment pH level. Whole-body K^+^ increased in the presence of Al [155]. Rainbow trout exposed to pH 4.2 for 4 d lost 5.3 mmol K^+^ kg^−1^, three-quarters by efflux across the gills and one‑quarter by excretion through the kidneys, but despite this, blood [K^+^] remained relatively constant (2−3 mM). |
|  | Effects of low pH on crayfish | When exposed to pH 4.0, the crayfish *O. propinquus* exhibited greater net efflux of K^+^ compared to controls exposed to pH 7.5 [111]. Similarly, hemolymph [K^+^] decreased in *A. astacus* exposed to pH 4.0 water compared to controls in pH 7.9 [112]. |

AFW, artificial freshwater; CA, carbonic anhydrase; CW, creek water; DI, deionized; EC, extracellular; FW, freshwater; IC, intracellular; KHE, K^+^/H^+^‑exchanger; *K*_m_, ion concentration at which the uptake rate is half of *J*_max_; PW, pond water; VHA, vacuolar-type H^+^-ATPase.

| Subsection | Aspect of Transport | Evidence |
| --- | --- | --- |
| Teleost fish | NCX | In isolated tilapia gill MR ionocytes, replacement of EC Na^+^ with the organic ion *N*‑methyl‑d‑glucamine^+^ increased IC [Ca^2+^] because this replacement removed the counter ion for the NCX, Na^+^ [30]. |
|  | Role of NKA in Ca^2+^ transport | An α‑subunit isozyme of NKA (*zatp1a1a.1*), a FXYD protein believed to regulate the transport ability of NKA (zfxyd11) and ECaC were localized to NaR ionocytes of zebrafish and were upregulated in low [Ca^2+^] water [156]. |
|  | Increase in ECaC at low [Ca^2+^] | In a study that gradually decreased the exposure of zebrafish from hard water to soft water, the greatest change was the increase in both gene and protein expression for ECaC [157]. In rainbow trout, expression of mRNA and the ECaC protein increased after exposure to low [Ca^2+^] water (0.02−0.03 μM) vs. normal FW (0.2−0.3 μM Ca^2+^) or to hypercapnia (i.e., 1.0 kPa CO_2_) [158]. |
|  | Location of Ca^2+^ transporters in specific ionocyte types | Research on the segregation of different transporters among different gill epithelial ionocyte types in rainbow trout [159] found that uptake of both Cd^2+^ and Ca^2+^ occurred preferentially in β-type ionocytes. Also, Cd^2+^ accumulation was inhibited when the experiment was conducted in Cl^−^‑free, phosphate-buffered saline (i.e., Cl^−^ salts were replaced by gluconate salts) [159], and ECaC were collocated with an AE on the apical membrane of β-type ionocytes [160]. The ECaC was located on the apical membranes of both pavement ionocytes and to MR ionocytes that also expressed NKA, presumably the β-type ionocytes [158]. |
|  | Absorption of Ca^2+^ from food | Rainbow trout absorbed 28% of the Ca^2+^ in a single experimental meal that supplied 5.9 mmol Ca^2+^ kg^−1^ fish body mass [161]. Addition of Ca^2+^ to the diet of rainbow trout reduced accumulation of Cd, which had also been added to their diet [162]. Moreover, Ca^2+^ or Cd^2+^ added to the diet reduced the uptake of Ca^2+^ and Cd^2+^ by the gills [163, 164]. For Ca^2+^, this occurs by the mechanisms that regulate uptake of Ca^2+^ in relation to plasma [Ca^2+^], such as elevation of [Ca^2+^] in the ionocytes or increased levels of the regulatory hormone, stanniocalcin [165]. Cd^2+^ entering the ionocytes across the basolateral membrane from the blood likely has a similar inhibitory effect on Ca^2+^‑ATPase [162]. |
| Aquatic insects | Evidence that Ca^2+^ uptake does not occur by the anal papillae | Removal of the anal papillae from larvae of the mosquito, *A. aegypti*, resulted in increased Ca^2+^ uptake [166]. |

| Subsection | Aspect of Transport | Evidence |  |
| --- | --- | --- | --- |
| Aquatic insects (continued) | Evidence that Ca^2+^ uptake does not occur by the anal papillae | Donini and O'Donnell [167] did not measure net flux of Ca^2+^ across the anal papillae of *A. aegypti*, but much earlier work [168] concluded that divalent ions were not absorbed by epithelial cells of the anal papillae. |  |
| Mollusca | Hemolymph [Ca^2+^] relative to [Ca^2+^] in PW vs. ASW | When *D. polymorpha* was transferred from PW (0.4 mM Ca^2+^) to ASW (1.0 mM Ca^2+^), hemolymph [Ca^2+^] remained greater than that in the water, but hemolymph [Ca^2+^] decreased in ASW (4.0 ± 0.2−4.5 ± 0.2 mM Ca^2+^ in PW vs. 2.2 ± 0.1−2.8 ± 0.1 mM Ca^2+^ in ASW) [169]. As with other ions, this change simply reversed when the *D. polymorpha* were transferred back to PW. |  |
|  | Ion regulation in extrapallial fluids of unionids. | In 5 species sampled from a single mussel bed in the Muskingum River, OH, mean extrapallial fluid [Ca^2+^] ranged from 2.1 to 3.2 mM (range: 1.3−39 mM) [170]. In *Amblema plicata*, extrapallial fluid [Ca^2+^] was maintained at 3 to 4 times the water concentration when water [Ca^2+^] and [Mg^2+^] were similar. |  |
|  | Evidence for the presence of an L-type ECaC and Ca^2+^/1H^+^-exchanger in *L. stagnalis* | Between the trochophore stage and hatching, the Ca^2+^ content of *L. stagnalis* embryos increased from 0.001 nmol embryo^−1^ to nearly 130 nmol embryo^−1^ [171]. Ca^2+^ uptake was inhibited by 10 μM dihydropyridine nifedipine or 100 μM phenylalkylamine verapamil (L-type Ca^+^-channel inhibitors), lanthanum (a ECaC inhibitor), ethoxzolamide (CA inhibitor), and bafilomycin (VHA inhibitor) [172]. In addition, ethoxzolamide and bafilomycin inhibited accumulation of titratable alkalinity, a measure of HCO_3_^−^/CO_3_^2−^, while bafilomycin inhibited efflux of H^+^. While EIPA, an inhibitor that is specific to cation/H^+^‑exchangers, could not be used with the ion‑specific microelectrodes used to measure ion fluxes of single embryos (EIPA interfered with both the Ca^2+^‑ and H^+^‑microelectrodes), [172] showed that EIPA inhibited uptake of Ca^2+^ and accumulation of titratable alkalinity by *L. stagnalis* egg masses, suggesting that the cation exchange may occur via an electrogenic Ca^2+^/1H^+^‑exchanger similar to that used by crustaceans [47, 173]. |  |
| Effects of Ca^2+^ transport by other ions in the water | Variation in affinity of PMCA for Ca^2+^ versus Cd^2+^ and Zn^2+^ | PMCA has about 100 times greater affinity for Cd^2+^ than it does for Ca^2+^ in rainbow trout, and inhibition of Ca^2+^ transport may occur at 0.05 to 0.1 μM Cd^2+^ [174]. For Zn^2+^, PMCA has about 10 times greater affinity than it does for Ca^2+^, and inhibition of Ca^2+^ transport in rainbow trout may occur at 15 μM Zn^2+^ [175] |  |
| Effects of Ca^2+^ transport by other ions in the water (continued) | Variation in affinity and toxicity of divalent metals between yellow perch and rainbow trout | Comparing yellow perch (96‑h LC50 = 72 μM Cd^2+^) and rainbow trout (96‑h LC50 = 0.168 μM Cd^2+^) in moderately hard ([1.20 mM CaCO_3_]) water [111], the *J*_max_ for yellow perch (0.54 ± 0.08 nmol Cd^2+^ g^−1^ wet weight h^−1^ in water with 100 μM Ca^2+^) was less than the J_max_ for rainbow trout (0.80 ± 0.06 nmol Cd^2+^ g^−1^ wet weight h^−1^). Also, the inhibitor constant, −*K*_i[Cd_^2+^_]_, for yellow perch (276.4 ± 30.1 nmol Cd^2+^) was greater than that for rainbow trout (155.2 ± 24.1 nmol Cd^2+^) [176]. This indicates that Cd^2+^ accumulated at a slower rate and required a greater concentration to inhibit Ca^2+^ uptake in yellow perch. These variables are characteristics of the transporter protein, in this case ECaC. |  |
|  | Variation in crayfish sensitivity to low pH | In *C. destructor* exposed to pH 4.5 and 500 μM Ca^2+^, carapace [Ca^2+^] decreased for up to 100 h but then increased again until the end of the experiment at 504 h [115]. In *O. rusticus*, another pH‑intolerant species, hemolymph [Na^+^] decreased in adults exposed to pH 4.0 for 96 h, but there was no significant difference in hemolymph [Na^+^] between adults of the more tolerant *C. robustus* exposed to pH 3.8 and pH 6.5 [114]. |  |
|  | Transport of divalent metals by the H2Na(Ca)E in Crustacea | The inhibition of Cd^2+^ uptake by increased [Ca^2+^] and increased total salinity indicates that the electrogenic H2Na(Ca)E transports divalent metals [177]. |  |
|  | Transport of divalent metals by the ECaC in Crustacea | In *Carcinus maenas*, influx of both Ca^2+^ and Cd^2+^ decreased in the presence of lanthanum, another ECaC blocker [178, 179], suggesting that both ions are taken up across the apical gill epithelia by ECaC. Also, in *C. maenas*, hemolymph [Cd^2+^] decreased with increasing water [Ca^2+^] [180], and uptake rates for Cd^2+^ or Zn^2+^ in *D. magna* decreased with increasing water [Ca^2+^]. In an experiment conducted to develop a biotic ligand model for Cd^2+^, the 48‑h LC50 for *D.* *pulex* increased with increasing Ca^2+^, although the range of [Cd^2+^] (0.15−1.4 μM) was much less than [Ca^2+^] (0.03−1.6 mM) [181], suggesting the affinity of the ECaC for Cd^2+^ was greater than that for Ca^2+^. |  |
|  | Inhibition of Ca^2+^ uptake by Cd^2+^ in a midge, *C. riparius* | In *C. riparius* exposed to Cd^2+^ (0.89−8,700 μM) in moderately hard water (1.4 mM CaCO_3_ hardness), whole‑body [Ca^2+^] had decreased significantly by the first measurement at 1 h [141]. Whole‑body [Ca^2+^] recovered by 48 h at the lowest 2 [Cd^2+^] (0.89 and 8.9 μM), but did not at the greater [Cd^2+^] (160 and 8,700 μM). In *C. riparius*, tolerance to Cd^2+^ may be related in part to relatively rapid removal and secretion of Cd^2+^ by the Malpighian tubules [182]. |  |
| Effects of Ca^2+^ transport by other ions in the water (continued) | Transport of divalent metals by Ca^2+^ transporters on ionocytes in Ephemeroptera and Trichoptera | Rates of Cd^2+^ and Zn^2+^ uptake varied from 11.6 ± 0.8 pmol Cd^2+^ g^−1^ h^−1^ for *Drunella flavilinea* to 0.09 ± 0.00 pmol Cd^2+^ g^−1^ h^−1^ for *Ameletus* sp. [183]. Uptake rates were not related to body weight among all species tested, and neither gill size nor water permeability were related to metal uptake rates. However, the relative numbers of ionocytes on the gills appear related to metal uptake rates. Among mayflies, *Drunella* have greater uptake rates and more ionocytes on their tracheal gills compared to *Siphonuris*. Working with a greater number of species within either the mayfly family, Ephemerellidae (6 species), or the caddisfly family, Hydropsychidae (5 species), Ca^2+^ uptake rates for the caddisflies were much less than those for the mayflies, while within either family, Ca^2+^ uptake rates were inversely related to body mass [184]. |  |
|  |  |  |  |
|  | Competitive inhibition of Cd^2+^ and Zn^2+^ uptake by Ca^2+^ and of Zn^2+^ uptake by Cd^2+^ | Cd^2+^ uptake was competitively inhibited when Ca^2+^ was increased from 0.18 to 0.71 mM in *Drunella*, while both Cd^2+^ and Zn^2+^ uptake were competitively inhibited when Ca^2+^ was increased from 0.18 to 1.4 mM. In *Ceratopsyche sparna*, the presence of Cd^2+^ inhibited Zn^2+^ uptake by 35%, while uptake of Cd^2+^ was not inhibited by the presence of Zn^2+^ [185]. Moreover, increasing the [Ca^2+^] to 1.4 mM from 0.03 mM somewhat inhibited both Cd^2+^ and Zn^2+^ uptake, while ruthenium red, a PMCA inhibitor, decreased the uptake of all 3 ions, indicating that the transporter PMCA is shared by these metals and Ca^2+^. |  |
|  | Effect of verapamil on Cd^2+^ uptake in Trichoptera and Ephemeroptera | Application of verapamil, an L-type Ca^2+^-channel inhibitor, had variable effects ranging from no effect in *Hydropsyche californica* to a 20% reduction in Cd^2+^ uptake in *D. flavilinea*, suggesting that ECaC differs in some way among these aquatic insects [183]. |  |
|  | Differences in the relative affinity of Ca^2+^ vs. Cd^2+^ or Zn^2+^ in Trichoptera and Ephemeroptera | Again looking at a number of species in the Ephemerellidae or Hydropsychidae, Poteat and Buchwalter [184] observed that while uptake rates for Zn^2+^ and Cd^2+^ were strongly correlated (*r* = 0.96, *P* < 0.0001), their respective correlations with Ca^2+^ uptake rates were weaker (Cd^2+^: *r* = 0.49, *P* = 0.10; Zn^2+^: *r* = 0.54, *P* = 0.07) and not statistically significant with the small sample size (*n* = 12) used in these analyses. Moreover, exposure to low [Cd^2+^] (0.89, 8.9, or 89 nM) or [Zn^2+^] (0.015, 0.15, or 1.5 μM) did not inhibit but apparently stimulated Ca^2+^ uptake, suggesting at least that the affinity of any shared transporters for Cd^2+^ or Zn^2+^ is not greater than that for Ca^2+^ and may differ from similar transporters in fish or crustaceans. |  |
|  | Inhibition of divalent metal uptake by elevated [Ca^2+^] in FW bivalves | Uptake of Pb^2+^, Mn^2+^, Cd^2+^, and Co^2+^ were each reduced in 2 bivalves, *Hyridella depressa* and *Velesunio ambiguus*, in water with elevated [Ca^2+^] compared to a background control (1.083 vs. 0.111 mM); the decrease in the uptake rate for each metal was consistent with competitive |  |
| Effects of Ca^2+^ transport by other ions in the water (continued) | Inhibition of divalent metal uptake by elevated [Ca^2+^] in FW bivalves (continued) | inhibition by Ca^2+^ in these transporters [186]. A similar effect was observed for Cd^2+^ in *E. complanata* [187]. |  |
|  | Inhibition of Ca^2+^ uptake by divalent metals in *L. stagnalis* | In *L. stagnalis*, uptake of Ca^2+^ was inhibited by exposure to 0.091 μM Pb^2+^ [188, 189], and hemolymph Ca^2+^ was reduced by exposure to 1.3 μM Co^2+^ [190]. |  |
|  | Elevation of [Ca^2+^] in hemolymph and extrapallial fluids of Mollusca by interaction between divalent metals and Ca^2+^-transporters | In *A. anatina* exposed to 0.002 μM Cd^2+^ for 1 h to 35 d, [Ca^2+^] increased from 3.94 ± 0.3 to 4.49 ± 0.7 mM in the hemolymph and from 3.92 ± 0.25 to 4.19 ± 0.32 mM in the extrapallial fluids, respectively, from control [Ca^2+^] (i.e., in mussels not exposed to Cd^2+^) of 3.14 ± 0.12 to 3.57 ± 0.32 mM in the hemolymph and 3.14 ± 0.35 to 3.59 ± 0.27 mM in the extrapallial fluids [191]. The [Ca^2+^] decreased again as the Cd^2+^ was depurated from the mussels following the end of the exposure to Cd^2+^. A similar increase in the hemolymph [Ca^2+^] occurred in *A. cygnea* exposed to 1 mM Cd^2+^ [192], and PMCA has been specifically identified with immunolabeling on the outer mantle epithelium of this species [193]. Moreover, exposure to Cd^2+^ also reduced tissue [Zn^2+^], another divalent metal, which can be transported by the ECaC and inhibits PMCA activity [191]. A similar decrease in tissue [Zn^2+^] with exposure to Cd^2+^ has been observed in the pulmonate gastropod, *L. stagnalis* [194]*.* However, exposure to low pH (i.e., 4.0−4.5) also has been shown to elevate hemolymph [Ca^2+^] in unionid mussels [125]. |  |

AE, Cl^−^/HCO_3_^−^-exchanger; ASW, artificial saltwater; CA, carbonic anhydrase; EC, extracellular; ECaC, epithelial Ca^2+^-channel; EIPA, 5‑(*N*‑ethyl‑*N*‑isopropyl)amiloride; FW, freshwater; H2Na(Ca)E, H^+^/2Na^+^(or Ca^2+^)-exchanger; IC, intracellular; *J*_max_, maximum uptake rate; LC50, median lethal concentration; MR, mitochondria-rich; NaR, Na^+^/K^+^-ATPase-rich; NCX, Na^+^/Ca^2+^-exchanger; NKA, Na^+^/K^+^-ATPase; PMCA, plasma membrane Ca^2+^-ATPase; PW, pond water; VHA, vacuolar-type H^+^-ATPase

| Subsection | Aspect of Transport | Evidence |
| --- | --- | --- |
| Teleost fish | Absorption of Mg^2+^ from their diet | Rainbow trout absorbed 60% of a single experimental meal that supplied 3.3 mmol Mg^2+^ kg^−1^ fish body mass [161]. |
|  | Absorption of Mg^2+^ by the gills | Common carp fed a diet low in Mg^2+^ became hypomagnesemic, mobilized Mg^2+^ from their bone, and could not meet their needs by absorbing Mg^2+^ from the water, which contained 0.17−0.23 mM Mg^2+^ [195]. |
|  | Acclimation to a low Mg^2+^ diet by adult tilapia | Adult tilapia fed a low Mg^2+^ diet (1 mmol kg^−1^ vs. 30 mmol kg^−1^ in the control) grew at a reduced rate [196], while MR ionocyte density and NKA activity increased. Also, Na^+^ uptake and loss both decreased. Therefore, the adult tilapia were able to acclimate to this diet and maintain blood ion homeostasis [197]. |
|  | Uptake of Mg^2+^ through the gills by tilapia | An uptake rate of 1 μmol Mg^2+^ d^−1^ through the gills was estimated for a 50 g tilapia fed a low Mg^2+^ diet in water with [Mg^2+^] = 0.2 mM, although the transporter is unknown [198]. |
|  | Exposure of juvenile rainbow trout to low Mg^2+^ diet and different water [Mg^2+^] | For juvenile rainbow trout, 1.9 mM Mg^2+^ in water was sufficient to meet the fishes’ Mg^2+^ requirement when fed a low-Mg diet [199]. A minimum diet was 3.2 μmol Mg^2+^ g^−1^ in fish that consumed 0.92−1.19 g dry weight of food over 28 d. The range in the diets tested was 3.2−30 μmol Mg^2+^ g^−1^. Fish in a greater water [Mg^2+^] (41 mM, 20 times the greatest of the other 4 treatments [2.1 mM Mg^2+^]) consumed less feed than fish in other treatments, exhibited greater whole-body [Mg^2+^], exhibited mortality after 2 d, and reached 48% mortality after 14 d. |
| Mollusca | Changes in hemolymph ion concentrations of *D. polymorpha* placed in Mg‑free AFW | Hemolymph [Mg^2+^] in *D. polymorpha*, which is a weaker hyper‑regulator than most other FW bivalves [87], decreased from 1.5 to 0.05 mM over 15 d when individuals were placed in Mg‑free AFW, whereas [Na^+^] and [K^+^] did not change. Hemolymph [Ca^2+^] decreased slightly but then increased to near its original concentration by 15 d, whereas [Cl^−^] decreased from 16.9 to 14.0 mM, and HCO_3_^−^ increased from approximately 1.0 to 4.1 mM [149]. |
|  | Mg^2+^ uptake in Mg-depleted *D. polymorpha* | When Mg‑depleted *D. polymorpha* were placed in AFW with [Mg^2+^] of 0.05, 0.1, or 0.2 mM, net Mg^2+^ uptake increased in relation to the AFW [Mg^2+^], and hemolymph [Mg^2+^] exceeded the AFW [Mg^2+^] after 17 h by 0.1, 0.1, and 0.3 mM, respectively [149]. |
| AFW, artificial freshwater; FW, freshwater; MR, mitochondria-rich; NKA, Na^+^/K^+^-ATPase. | | |

| Subsection | Aspect of Transport | Evidence |
| --- | --- | --- |
| Teleost fish | Isoforms of SLC26a and their location on ionocytes of zebrafish gills | The most common isoforms in zebrafish gills are SLC26a3, SLC26a4 and SLC26a6, and at least SLC26a3 is located in ionocytes on gill filaments and at the base of the gill lamellae, which may or may not be enriched with NKA [200]. The NaR ionocytes have, at least, AE on the apical membrane and VHA on the basolateral membrane [200]. |
|  | Upregulation of SLC26 mRNA and Cl^−^ uptake | When zebrafish were placed in waters with low (0.02 mM) vs. high (2.0 mM), ambient [Cl^−^], relative expression of gill SLC26 mRNA was substantially increased after 3 to 10 d [200]. Similarly, when zebrafish were exposed to elevated [NaHCO_3_], relative expression of gill SLC26 mRNA increased and Cl^−^ uptake rates increased [200]. Studies have also observed increased expression of cytosolic CA mRNA or protein levels in fish placed in soft, low [Cl^−^] waters [201, 202]. |
|  | Metabolon for Cl^−^ uptake | CA is localized close to AE on the apical membrane of an ionocyte specialized for Cl^−^ uptake, and base (i.e., HCO_3_^−^) excretion increases local [HCO_3_^−^] sufficiently to drive electroneutral exchange of Cl^−^ and HCO_3_^−^ by the exchanger (Figure 1). At the same time, VHA on the basolateral membrane, which is invaginated to place it closer to the CA and AE on the apical membrane, removes H^+^ produced by CA and transports it across the basolateral membrane out of the cell maintaining the shift in the reaction equilibrium to produce more HCO_3_^−^ [203]. This ionoregulation activity is facilitated by separation of transporters among different ionocyte types, and Cl^−^ uptake along with greater levels of VHA occur in β-type MR ionocytes in rainbow trout [204]. |
|  | Interspecific differences in inhibition of Cl^−^ influx | In neon tetra, Cl^−^ influx was inhibited at least 60% by SITS, an AE inhibitor, or by thiocyanate (SCN^−^), a Cl^−^-channel inhibitor. In goldfish, Cl^−^ influx was completely inhibited by SITS but was not inhibited by SCN^−^. |
| Crustacea | Separation of gas and ion exchange among different gill filaments and lamina of *Pontastacus leptodactylus* | In *P. leptodactylus*, asymmetrical perfusion of one type of filament with artificial water containing 0.0001 to 1 mM Na^+^ caused electrical potential differences across single filaments, while perfusion of a second type of filament did not cause electrical potential differences [205]. The first filament type was involved in ion uptake, as the epithelium was mitochondria-rich and had an invaginated basolateral membrane as is characteristic of ion‑transporting epithelia. The second filament type was involved in gas exchange, as morphologically its epithelium was much thinner, lacked mitochondria, and was composed of squamous cells [206]. Moreover, the laminae were involved in Cl^−^ uptake [205, 207]. |

| Subsection | Aspect of Transport | Evidence |
| --- | --- | --- |
| Aquatic insects | Differences in Cl^−^ uptake among mosquito larvae | Among the larvae of 4 different mosquitos that inhabit phytotelmata with low ionic water, hemolymph [Cl^−^] ranged from 58 to 78 mM and Cl^−^ uptake rates ranged from < 0.01 to 0.6 nmol g^−1^ h^−1^ [116]. When the mosquito larvae were placed in low pH (3.5) water, Cl^−^ uptake rates unexpectedly increased, possibly suggesting a different uptake mechanism, such as an NCC, although this hypothesis has not been tested with molecular studies. Like Na^+^, Cl^−^ uptake increased by 4-7 times when the mosquitoes were placed in more saline water [116]. Among different populations of *A. aegypti* (a colony maintained by J. Shaw at University of Newcastle upon Tyne vs. one at Ohio State University), Stobbart [208] found that *K*_m_ differed for Cl^−^ (500 μM vs. 0.2 μM, respectively). |
|  | Variation in body wall impermeability to Cl^−^ among a euryhaline and 2 FW species of caddisflies | As with Na^+^, the body wall of the euryhaline caddisfly *L. affinis* was relatively impermeable to Cl^−^ [69], and hemolymph [Cl^−^] was maintained at higher levels at lower external [Cl^−^] (< 100 mM), while mean hemolymph [Cl^−^] remained less than 150 mM at greater external [Cl^−^] (up to 400 mM). Conversely, the FW caddisflies *L. stigma* and *A. nervosa* maintained hemolymph [Cl^−^] at slightly greater levels at very low external [Cl^−^] (< 15 mM), and hemolymph [Cl^−^] increased but was less than the external concentrations at greater external concentrations (15 to 220 mM) [71]. These 2 species generally excreted urine with greater [Cl^−^] than that in their hemolymph at these greater external [Cl^−^]. As hemolymph [Na^+^] and [Cl^−^] were measured on the same larvae, this change to hypotonic hemolymph occurred coincident with high mortality at [NaCl] > 120 mM. |
| Mollusca | Evidence showing exchange of HCO_3_^−^/Cl^−^ exchange is independent of Na^+^ | In *L. subrostrata* maintained in 0.1 mM sodium sulfate (Na_2_SO_4_) solution for about 60 d (vs. a control of DI water), hemolymph HCO_3_^−^ increased from 11.0 to 13.5 mM, while hemolymph Cl^−^ decreased by 89 to 94% (from 11.4 to 1.3 mM) [209]. When the Cl^−^‑depleted, mussels were placed in 1.0 mM solutions of NaCl or the organic salt, choline Cl, net Cl^−^ influx was 0.5−1.0 μmol g^−1^ h^−1^ for the first 3 d compared to near 0 for mussels placed in 0.1 mM Na_2_SO_4_ solutions, while in undepleted mussels placed in a choline Cl solution, hemolymph [HCO_3_^−^] decreased from 12.9 to 8.6 mM. |
|  | Changes in hemolymph [Cl^−^] with changes in water NaCl salinity | When maintained in PW, hemolymph [Cl^−^] (14.6−16.2 mM) of *D. polymorpha* was greater than that of the PW [Cl^−^] (1.4 mM) [169], but when transferred to 10% ASW, the hemolymph [Cl^−^] (44.3−46.9 mM) approached that of the 10% ASW (52.4 mM Cl^−^). As with the other ions, the changes reversed when the mussels were returned to PW. |
| Mollusca (continued) | Changes in hemolymph [Cl^−^] with decreased pH | As in fish and decapods, unionid mussels exposed to low pH (i.e., 4.0−4.5) in either soft (0.11 mM Ca^2+^) or hard (0.46 mM Ca^2+^) water showed decreased hemolymph [Cl^−^], possibly because the VHA activity is inhibited by elevated ambient [H^+^] [125]. In turn, inhibition of VHA activity would affect the production of HCO_3_^−^ from CO_2_ by CA for exchange for Cl^−^ by the AE [203]. |
| Effects of Cl^−^ transport by other ions in the water | Effects of decreased pH on blood [Cl^−^] in fish | In rainbow trout exposed to pH 4.2 for 4 d, total Cl^−^ efflux was 9.4 mmol kg^−1^, with slightly more than two‑thirds by efflux across the gills and the remainder by excretion through the kidneys, resulting in a decrease in blood Cl^−^ of 21.6 mM over the 4 d [95]. Blood [HCO_3_^−^] was also depressed by the acid disturbance, although blood *P*_CO2_ was relatively unchanged, suggesting that acid stress (pH decreased from 7.8 to 7.2 during the 4 d) may have inhibited production of HCO_3_^−^, which in turn inhibited uptake of Cl^−^ by the AE. Similar changes in pH, HCO_3_^−^ and Cl^−^ were observed in *Catostomus commersonii* (white sucker) exposed to pH 4.3 in low [Ca^2+^] (82−92 μM) water [210]. |
|  | Effects of decreased pH on blood [Cl^−^] in Crustacea | Exposure of the crayfish *O. propinquus* to pH 4.0 resulted in net efflux of Cl^−^ from the hemolymph that resulted from a decrease in Cl^−^ influx with little change in Cl^−^ efflux [111]. This was accompanied by a decrease in hemolymph pH and an increase in *P*_CO2_, which decreased HCO_3_^−^ that could be exchanged for Cl^−^. Similarly in the amphipod *Gammarus fossarum*, hemolymph Cl^−^ as well as Na^+^ decreased with decreasing pH [211]. Although the authors did not investigate any mechanisms, the current model suggests that inhibition of H^+^ excretion, which reduces concomitant uptake of Na^+^ and is the cause of the reduction in hemolymph Na^+^ also decreases HCO_3_^−^ production by the CA-catalyzed hydrolysis of CO^2^ along with retention of the HCO_3_^−^ to maintain hemolymph acid/base balance. As a result, there is a concomitant reduction in Cl^−^ uptake by the AE that reduces hemolymph [Cl^−^] [203]. |
|  | Effects of increased pH on blood [Cl^−^] in fish | Rainbow trout exposed to pH 9.5 (0.6 mM Na^+^, 0.8 mM Cl^−^) after acclimation to pH 8.0 exhibited a decrease in Cl^−^ influx (from ≈ 275 μmol kg^−1^ h^−1^ in control to < 50 μmol kg^−1^ h^−1^ after 4‑h exposure to pH 9.5). The [Cl^−^] then increased and was not less than the control after 72 h [212]. As in experiments with low-salinity water, MR ionocyte density and total apical surface increased on gill filaments of fish exposed to pH 9.5. However, in this case, the decreased uptake may also be caused, in part, by a decrease in the availability of HCO_3_^−^ for exchange with Cl^−^ [213]. In addition to increased blood pH, decreased *P_CO2_* and decreased [H^+^], metabolic alkalosis in rainbow trout exposed to pH 9.5 decreased blood [HCO_3_^−^] from 7.50 mM in control fish at pH 8.0 to between 2.58 and 5.04 mM. In rainbow trout exposed to pH 10.5 with or without a glycine buffer, |
| Effects of Cl^−^ transport by other ions in the water (continued) | Effects of increased pH on blood [Cl^−^] in fish (continued) | effects of increased pH on blood [Cl^−^] in fish blood [HCO_3_^−^] increased and [Cl^−^] decreased because of inhibition of the AE [214], possibly associated with an increase in the alkalinity gradient across the apical membrane. As also observed by Wilkie and Wood [212], this ion perturbation was reversed after 1 to 2 d associated with an increase in the proportional exposure of MR ionocytes on the gills. |
|  | NO_2_^−^ uptake in crayfish | Uptake of NO_2_^−^ in *A. astacus* or *Pacifastacus leniusculus* showed Michaelis‑Menten kinetics, suggesting active transport, and NO_2_^−^ competitively inhibited uptake of Cl^−^ [215] as well as Br^−^ [216]. Moreover, uptake rates of NO_2_^−^ increased 24 h following induction of hypercapnia by bubbling the water with 1% CO_2_ and 99% air when hemolymph [HCO_3_^−^] increased. In parallel with the accumulation of NO_2_^−^, NO_3_^−^ also accumulated in the hemolymph but its concentrations were unrelated to any other ion concentration in the water, indicating that it was produced by processes that detoxify the NO_2_^−^ by oxidizing it to NO_3_^−^ [216]. However, there are no effects on respiration similar to those in fish because NO_2_^−^ does not oxidize hemocyanin as it does hemoglobin. When the crayfish were returned to water without NO_2_^−^, hemolymph NO_2_^−^ was eliminated by 7 d and [Cl^−^] returned to control levels, but [NO_3_^−^] remained elevated, suggesting low gill‑epithelial‑membrane permeability for NO_3_^−^ [216]. |
|  | Additional effect of NO_2_^−^ on respiration in fish | In fish, NO_2_^−^ has an added effect on respiration because it oxidizes oxyhemoglobin to methemoglobin converting Fe^2+^ to Fe^3+^ and forms NO_3_^−^ [217]. |
| AE, Cl^−^/HCO_3_^−^-exchanger; ASW, artificial saltwater; CA, carbonic anhydrase; DI, deionized; FW, freshwater; *K*_m_, ion concentration at which the uptake rate is half of *J*_max_; MR, mitochondria-rich; NaR, Na^+^/K^+^-ATPase-rich; NCC, Na^+^/Cl^−^‑cotransporter; NKA, Na^+^/K^+^-ATPase; *P*_CO2_, partial pressure of CO_2_; PW, pond water; SITS, disodium 4 acetamido‑4′ isothiocyanatostilbene-2, 2′ disulfonate; SLC, solute carrier; VHA, vacuolar-type H^+^-ATPase. | | |

| Subsection | Aspect of Transport | Evidence | |
| --- | --- | --- | --- |
| Teleost fish | Fish where the described response to metabolic acidosis has been observed | Such a response to metabolic acidosis resulting from external hypercapnia has been described for common carp [218], *Ictalurus punctatus* (channel catfish) [219], and rainbow trout [220]. |  |
|  | Comparison of the response to metabolic acidosis by American eel (*Anguilla rostrata*) and rainbow trout | In a comparison of American eel and rainbow trout, fish were exposed to external hypercapnia which induced metabolic acidosis and reduced blood pH 0.4 and 0.35 units, respectively [221]. In American eels, Na^+^/H^+^ exchange occurred to compensate for the acidosis, exporting the H^+^, as both branchial *J*_in_^Na+^ and *J*_net_^Na+^ increased relative to controls. In rainbow trout, *J*_net_^Cl−^ was negative as HCO_3_^−^ was accumulated to compensate for the acidosis. Following removal from external hypercapnia, blood pH increased to slightly greater than the original pH as a result of alkalosis. The rainbow trout rapidly recovered from this alkalosis by increasing *J*_net_ ^Cl−^, exporting HCO_3_^−^, but the American eels were unable to increase Cl^−^/HCO_3_^−^ exchange and had not recovered by 24 h [222]. |  |
|  | Response to increases in acid or base load by channel catfish | Channel catfish infused with either NH_4_Cl or NaHCO_3_ to increase their acid or base loads, respectively, excreted H^+^ in response to acidosis and HCO_3_^−^ in response to alkalosis, mostly through the gills [223]. |  |
| Crustacea | Responses of Cl− and Na^+^ to excretion of acid and base units following hemolymph acidosis in *P. leniusculus* | The small net loss of Cl^−^ occurred because of decreased Cl^−^ uptake in exchange for HCO_3_^−^. Na^+^ was unchanged because excretion of acid units (H^+^) maintained the uptake of Na^+^. Upon return to normoxia, the levels of HCO_3_^−^ + CO_3_^2−^ decreased and H^+^ excretion decreased, while net Na^+^ efflux and net Cl^−^ influx brought Na^+^, Cl^−^, and acid‑base balance back to control levels. Part of these exchanges occurred via the antennal gland, and the urine was acidified, while HCO_3_^−^ was reabsorbed. Urinary Na^+^ efflux increased during hyperoxia, and Cl^−^ efflux decreased, particularly after 36 h [224, 225]. |  |
| Mollusca | Response of hemolymph [HCO_3_^−^] to increased hemolymph pH | Although total [CO_3_] were the same (0.2 mM) in PW and 10% ASW, hemolymph [HCO_3_^−^] of *D. polymorpha* increased from 2.38−2.98 mM in PW to 5.31−6.37 mM when the mussels were moved to 10% ASW [169]. Hemolymph pH also increased from 7.74−7.96 in PW to 8.17−8.25 in 10% ASW, while *P*_CO2_ was variable but showed no trend (1.49−3.21 mm Hg). [HCO_3_^−^] again decreased when the mussels were returned to PW. This sequence suggests that HCO_3_^−^ was not excreted to counter the increase in pH. |  |

| Subsection | Aspect of Transport | Evidence |
| --- | --- | --- |
|  | Responses of CA activity in an estuarine and 2 FW bivalves to alterations in salinity | *Rangia cuneata* had greater gill CA activity in 1‰ salinity water, where these bivalves’ blood [Na^+^] and [Cl^–^] were greater than those in the water, compared to 10‰ salinity water, where *Rangia*’s blood [Na^+^] and [Cl^–^] were approximately equal to those in the water [226]. Similarly, the 2 FW bivalves had greater gill CA activity in DI water than in AFW, although *C. fluminea* had greater gill CA activity in both waters because its blood [Na^+^] and [Cl^−^] were greater than those of *L. subrostrata*. Moreover, because the 2 FW bivalves both maintained greater ionic gradients than *R. cuneata*, their gill CA activities were greater than in *R. cuneata*. Conversely, lower hemolymph [Na^+^] and [Cl^−^] in *Anodonta* spp. exposed to Cd^2+^ appear likely to result from inhibition of CA, which reduces the availability of H^+^ and HCO_3_^−^ for exchange [192, 227]. |
|  | Responses of dissolved [Ca^2+^] and pH to inhibition of CA | The addition of acetazolamide, a CA inhibitor, decreased pH and dissolved Ca^2+^ in mantle homogenates of *A. cygnea* [228]. Similarly, inhibition of CA by Cd^2+^ increased [Ca^2+^] in the hemolymph and extrapallial fluids of *A. anatina* [191, 227]. |
| AFW, artificial freshwater; ASW, artificial saltwater; CA, carbonic anhydrase; DI, deionized; FW, freshwater; *J_in_^NA+^*, uptake rate of sodium; *J*_net_*^X^*, net uptake rate of ion; *P*_CO2_, partial pressure of CO_2_; PW, pond water. | | |

| Subsection | Aspect of Transport | Evidence |
| --- | --- | --- |
| Teleost fish (toad) | Characteristics of SO_4_^2−^ transport in European toad (*Bufo bufo*) | When European toad skin was mounted in an Ussing chamber and clamped at −80 mV and with apical Na^+^ conductance eliminated by treatment with amiloride (a Na^+^-transporter inhibitor), transepithelial conductance of SO_4_^2−^ was identical to voltage activated Cl^−^ conductance [229]. |
| Mollusca | Characteristics of SO_4_^2−^ transport in *D. polymorpha* | *D. polymorpha* maintained hemolymph [SO_4_^2−^] 4 to 24 times greater than PW [SO_4_^2−^] (0.2 mM), accumulated ^35^SO_4_ in its hemolymph from the bathing medium, cleared ^35^SO_4_ at a rate 19−21% of that of polyethylene glycol, and the ^35^SO_4_ accumulation was inhibited by treatment with DIDS, an AE inhibitor [230]. However, *D. polymorpha* that had been acclimated to 10% ASW (2.8 mM SO_4_^2−^) cleared ^35^SO_4_ at a similar rate to polyethylene glycol, suggesting that SO_4_^2−^ was not reabsorbed by the kidney [230], as these mussels did not need to conserve this SO_4_^2−^ because their hemolymph concentrations were greater [169]. |
| AE, anion exchanger; ASW, artificial saltwater; DIDS, 4,4′-disorthiocyanato-stilbeen 2,2′-disulfonic acid; PW, pond water | | |

**REFERENCES**

[1] Cutler CP, Cramb G. 2002. Branchial expression of an aquaporin 3 (AQP-3) homologue is downregulated in the European eel *Anguilla anguilla* following seawater acclimation. *J Exp Biol* 205:2643-2651.

[2] Lignot J-H, Cutler CP, Hazon N, Cramb G. 2002. Immunolocalisation of aquaporin 3 in the gill and the gastrointestinal tract of the European eel *Anguilla anguilla* (L.). *J Exp Biol* 205:2653-2663.

[3] Tipsmark CK, Sørensen KJ, Madsen SS. 2010. Aquaporin expression dynamics in osmoregulatory tissues of Atlantic salmon during smoltification and seawater acclimation. *J Exp Biol* 213:368-379.

[4] Zheng H, Dietz TH. 1998. Paracellular solute uptake in the freshwater bivalves *Corbicula fluminea* and *Toxolasma texasensis*. *Biol Bull* 194:170-177.

[5] Kapoor NN. 1979. Osmotic regulation and salinity tolerance of the stonefly nymph, *Paragnetina media*. *J Insect Physiol* 25:17-20.

[6] Peterson MS. 1988. Comparative physiological ecology of centrarchids in hyposaline environments. *Can J Fish Aquat Sci* 45:827-833.

[7] Gonçalves AMM, Castro BB, Pardal MA, Gonçalves F. 2007. Salinity effects on survival and life history of two freshwater cladocerans (*Daphnia magna* and *Daphnia longispina*). *Ann Limnol Internat J Limnol* 43:13-20.

[8] Weider LJ, Hebert PDN. 1987. Ecological and physiological differentiation among low-arctic clones of *Daphnia pulex*. *Ecology* 68:188-198.

[9] Teschner M. 1995. Effects of salinity on the life history and fitness of *Daphnia magna*: variability within and between populations. *Hydrobiologia* 307:33-41.

[10] Jordan PJ, Deaton LE. 1999. Osmotic regulation and salinity tolerance in the freshwater snail *Pomacea bridgesi* and the freshwater clam *Lampsilis teres*. *Comp Biochem Phys A* 122:199-205.

[11] Hanson JA, Dietz TH. 1976. The role of free amino acids in cellular osmoregulation in the freshwater bivalve *Ligumia subrostrata* (Say). *Can J Zool* 54:1927-1931.

[12] de Boeck G, Vlaeminck A, van der Linden A, Blust R. 2000. The energy metabolism of common carp (*Cyprinus carpio*) when exposed to salt stress: An increase in energy expenditure or effects of starvation? *Physiol Biochem Zool* 73:102-111.

[13] Wood CM, Iftikar FI, Scott GR, De Boeck G, Sloman KA, Matey V, Valdez Domingos FX, Duarte RM, Almeida-Val VMF, Val AL. 2009. Regulation of gill transcellular permeability and renal function during acute hypoxia in the Amazonian oscar (*Astronotus ocellatus*): new angles to the osmorespiratory compromise. *J Exp Biol* 212:1949-1964.

[14] Bagherie-Lachidan M, Wright SI, Kelly SP. 2008. Claudin-3 tight junction proteins in *Tetraodon nigroviridis:* cloning, tissue-specific expression, and a role in hydromineral balance. *Am J Phys-Reg I* 294:R1638-R1647.

[15] Bagherie-Lachidan M, Wright SI, Kelly SP. 2009. Claudin-8 and -27 tight junction proteins in puffer fish *Tetraodon nigroviridis* acclimated to freshwater and seawater. *J Comp Phys B* 179:419-431.

[16] Tipsmark CK, Kiilerich P, Nilsen TO, Ebbesson LOE, Stefansson SO, Madsen SS. 2008. Branchial expression patterns of claudin isoforms in Atlantic salmon during seawater acclimation and smoltification. *Am J Phys-Reg I* 294:R1563-R1574.

[17] Tipsmark CK, Baltzegar DA, Ozden O, Grubb BJ, Borski RJ. 2008. Salinity regulates claudin mRNA and protein expression in the teleost gill. *Am J Phys-Reg I* 294:R1004-R1014.

[18] Chasiotis H, Effendi JC, Kelly SP. 2009. Occludin expression in goldfish held in ion-poor water. *J Comp Phys B* 179:145-154.

[19] Chasiotis H, Kolosov D, Kelly SP. 2012. Permeability properties of the teleost gill epithelium under ion-poor conditions. *Am J Phys-Reg I* 302:R727-R739.

[20] Chasiotis H, Wood CM, Kelly SP. 2010. Cortisol reduces paracellular permeability and increases occludin abundance in cultured trout gill epithelia. *Mol Cell Endocrinol* 323:232-238.

[21] Kelly SP, Chasiotis H. 2011. Glucocorticoid and mineralocorticoid receptors regulate paracellular permeability in a primary cultured gill epithelium. *J Exp Biol* 214:2308-2318.

[22] Bui P, Bagherie-Lachidan M, Kelly SP. 2010. Cortisol differentially alters claudin isoforms in cultured puffer fish gill epithelia. *Mol Cell Endocrinol* 317:120-126.

[23] Chasiotis H, Kelly SP. 2011. Permeability properties and occludin expression in a primary cultured model gill epithelium from the stenohaline freshwater goldfish. *J Comp Phys B* 181:487-500.

[24] Fenwick JC, Wendelaar Bonga SE, Flik G. 1999. In vivo bafilomycin-sensitive Na^+^ uptake in young freshwater fish. *J Exp Biol* 202:3659-3666.

[25] Parks SK, Tresguerres M, Goss GG. 2008. Theoretical considerations underlying Na^+^ uptake mechanisms in freshwater fishes. *Comp Biochem Physiol C* 148:411-418.

[26] Parks SK, Tresguerres M, Galvez F, Goss GG. 2010. Intracellular pH regulation in isolated trout gill mitochondrion-rich (MR) cell subtypes: Evidence for Na^+^/H^+^ activity. *Comp Biochem Phys A* 155:139-145.

[27] Parks SK, Tresguerres M, Goss GG. 2009. Cellular mechanisms of Cl^−^ transport in trout gill mitochondrion-rich cells. *Am J Phys-Reg I* 296:R1161-R1169.

[28] Wilson JM, Laurent P, Tufts BL, Benos DJ, Donowitz M, Vogl AW, Randall DJ. 2000. NaCl uptake by the branchial epithelium in freshwater teleost fish: an immunological approach to ion-transport protein localization. *J Exp Biol* 203:2279-2296.

[29] Perry SF, Beyers ML, Johnson DA. 2000. Cloning and molecular characterisation of the trout (*Oncorhynchus mykiss*) vacuolar H(^+^)-ATPase B subunit. *J Exp Biol* 203:459-470.

[30] Li J, Eygensteyn J, Lock RAC, Wendelaar Bonga SE, Flik G. 1997. Na^+^ and Ca^2+^ homeostatic mechanisms in isolated chloride cells of the teleost *Oreochromis mossambicus* analysed by confocal laser scanning microscopy. *J Exp Biol* 200:1499-1508.

[31] Kumai Y, Perry SF. 2012. Mechanisms and regulation of Na+ uptake by freshwater fish. *Resp Physiol Neurobiol* 184:249-256.

[32] Perry SF, Gilmour KM. 2006. Acid–base balance and CO_2_ excretion in fish: Unanswered questions and emerging models. *Resp Physiol Neurobiol* 154:199-215.

[33] Alvarez de la Rosa D, Canessa CM, Fyfe GK, Zhang P-P. 2000. Structure and regulation of amiloride-sensitive sodium channels. *Annu Rev Physiol* 62:573-594.

[34] Bucking C, Wood CM. 2006. Gastrointestinal processing of Na^+^, Cl^−^, and K^+^ during digestion: implications for homeostatic balance in freshwater rainbow trout. *Am J Phys-Reg I* 291:R1764-R1772.

[35] Smith NF, Eddy FB, Talbot C. 1995. Effect of dietary salt load on transepithelial Na^+^ exchange in freshwater rainbow trout (*Oncorhynchus mykiss*). *J Exp Biol* 198:2359-2364.

[36] D'Cruz Leela M, Wood Chris M. 1998. The influence of dietary salt and energy on the response to low pH in juvenile rainbow trout. *Physiol Zool* 71:642-657.

[37] Esbaugh AJ, Perry SF, Bayaa M, Georgalis T, Nickerson J, Tufts BL, Gilmour KM. 2005. Cytoplasmic carbonic anhydrase isozymes in rainbow trout *Oncorhynchus mykiss*: Comparative physiology and molecular evolution. *J Exp Biol* 208:1951-1961.

[38] Georgalis T, Perry SF, Gilmour KM. 2006. The role of branchial carbonic anhydrase in acid-base regulation in rainbow trout *(Oncorhynchus mykiss*). *J Exp Biol* 209:518-530.

[39] Esaki M, Hoshijima K, Kobayashi S, Fukuda H, Kawakami K, Hirose S. 2007. Visualization in zebrafish larvae of Na^+^ uptake in mitochondria-rich cells whose differentiation is dependent on foxi3a. *Am J Phys-Reg I* 292:R470-R480.

[40] Wieczorek H, Brown D, Grinstein S, Ehrenfeld J, Harvey WR. 1999. Animal plasma membrane energization by proton-motive V-ATPases. *Bioessays* 21:637-648.

[41] Boisen AMZ, Amstrup J, Novak I, Grosell M. 2003. Sodium and chloride transport in soft water and hard water acclimated zebrafish (*Danio rerio*). *Biochim Biophys Acta* 1618:207-218.

[42] Edwards SL, Tse CM, Toop T. 1999. Immunolocalisation of NHE3-like immunoreactivity in the gills of the rainbow trout (*Oncorhynchus mykiss*) and the blue-throated wrasse (*Pseudolabrus tetrious*). *J Anat* 195:465-469.

[43] Yan J-J, Chou M-Y, Kaneko T, Hwang P-P. 2007. Gene expression of Na^+^/H^+^ exchanger in zebrafish H^+^-ATPase-rich cells during acclimation to low-Na^+^ and acidic environments. *Am J Physiol-Cell Ph* 293:C1814-C1823.

[44] Lin L-Y, Horng J-L, Kunkel JG, Hwang P-P. 2006. Proton pump-rich cell secretes acid in skin of zebrafish larvae. *Am J Physiol-Cell Ph* 290:C371-C378.

[45] Shih T-H, Horng J-L, Liu S-T, Hwang P-P, Lin L-Y. 2011. Rhcg1 and NHE3b are involved in ammonium-dependent sodium uptake by zebrafish larvae acclimated to low-sodium water. *Am J Phys-Reg I* 302:R84-R93.

[46] Kumai Y, Perry SF. 2011. Ammonia excretion via Rhcg1 facilitates Na+ uptake in larval zebrafish, *Danio rerio,* in acidic water. *Am J Phys-Reg I* 301:R1517-R1528.

[47] Ahearn GA. 1996. The invertebrate electrogenic 2Na^+^/1H^+^ exchanger: Polyfunctional epithelial workstation. *News Physiol Sci* 11:31-35.

[48] Shaw J. 1960. The absorption of sodium ions by the crayfish *Astacus pallipes* Lereboullet : III. The effect of other cations in the external solution. *J Exp Biol* 37:548-556.

[49] de Couet HG, Busquets-Turner L, Gresham A, Ahearn GA. 1993. Electrogenic 2 Na/1 H antiport in crustacean epithelium is inhibited by a monoclonal antibody. *Am J Phys-Reg I* 264:R804-R810.

[50] Glover CN, Wood CM. 2005. Physiological characterisation of a pH- and calcium-dependent sodium uptake mechanism in the freshwater crustacean, *Daphnia magna*. *J Exp Biol* 208:951-959.

[51] Glover CN, Pane EF, Wood CM. 2005. Humic substances influence sodium metabolism in the freshwater crustacean *Daphnia magna*. *Physiol Biochem Zool* 78:405-416.

[52] Zare S, Greenaway P. 1997. Ion transport and the effects of moulting in the freshwater crayfish *Cherax destructor* (Decapoda : Parastacidae). *Aust J Zool* 45:539-551.

[53] Wheatly MG, Ignaszewski LA. 1990. Electrolyte and gas exchange during the moulting cycle of a freshwater crayfish. *J Exp Biol* 151:469-483.

[54] Wheatly MG, Gannon AT. 1995. Ion regulation in crayfish: Freshwater adaptations and the problem of molting. *Am Zool* 35:49-59.

[55] Lucu Č, Towle DW. 2003. Na^+^+K^+^-ATPase in gills of aquatic crustacea. *Comp Biochem Phys A* 135:195-214.

[56] Sarver RG, Flynn MA, Holliday CW. 1994. Renal Na,K-ATPase and osmoregulation in the crayfish, *Procambarus clarkii*. *Comp Biochem Phys A* 107:349-356.

[57] del Duca O, Nasirian A, Galperin V, Donini A. 2011. Pharmacological characterisation of apical Na+ and Cl– transport mechanisms of the anal papillae in the larval mosquito *Aedes aegypti*. *J Exp Biol* 214:3992-3999.

[58] Pullikuth AK, Aimanova K, Kang'ethe W, Sanders HR, Gill SS. 2006. Molecular characterization of sodium/proton exchanger 3 (NHE3) from the yellow fever vector, *Aedes aegypti*. *J Exp Biol* 209:3529-3544.

[59] Rheault MR, Okech BA, Keen SBW, Miller MM, Meleshkevitch EA, Linser PJ, Boudko DY, Harvey WR. 2007. Molecular cloning, phylogeny and localization of AgNHA1: the first Na^+^/H^+^ antiporter (NHA) from a metazoan, *Anopheles gambiae*. *J Exp Biol* 210:3848-3861.

[60] Smith KE, Raymond SL, Valenti ML, Smith PJS, Linser PJ. 2010. Physiological and pharmacological characterizations of the larval *Anopheles albimanus* rectum support a change in protein distribution and/or function in varying salinities. *Comp Biochem Phys A* 157:55-62.

[61] Komnick H, Rhees RW, Abel JH, Jr. 1972. The function of ephemerid chloride cells: Histochemical, autoradiographic and physiological studies with radioactive chloride on *Callibaetis*. *Cytobiologie* 5:65-82.

[62] Wichard W, Komnick H. 1971. Electron microscopical and histochemical evidence of chloride cells in tracheal gills of mayfly larvae. *Cytobiologie* 3:215-228.

[63] Filshie BK, Campbell IC. 1984. Design of an insect cuticle associated with osmoregulation: The porous plates of chloride cells in a mayfly nymph. *Tissue Cell* 16:789-803.

[64] Kapoor NN. 1980. Relationship between gill Na^+^,K^+^-activated ATPase activity and osmotic stress in the plecopteran nymph, *Paragnetina media*. *J Exp Zool* 213:213-218.

[65] Wichard W, Komnick H. 1974. Structure and function of the respiratory epithelium in the tracheal gills of stonefly larvae. *J Insect Physiol* 20:2397-2406.

[66] Kapoor NN, Zachariah K. 1973. Presence of specialized cellular complexes in the tracheal gills of stonefly nymph, *Paragnetina media* (Walker). *Experientia* 29:848-850.

[67] Kapoor NN, Zachariah K. 1973. A study of specialized cells of the tracheal gills of *Paragnetina media* (Plecoptera). *Can J Zool* 51:983-986.

[68] Colby C. 1972. Salt and water balance in stonefly naiads, *Pteronarcys californica* Newport. *Comp Biochem Phys A* 41:851-860.

[69] Sutcliffe DW. 1961. Studies on salt and water balance in caddis larvae (Trichoptera): I. Osmotic and ionic regulation of body fluids in *Limnephilus affinis* Curtis. *J Exp Biol* 38:501-519.

[70] Sutcliffe DW. 1962. Studies on salt and water balance in caddis larvae (Trichoptera): III. Drinking and excretion. *J Exp Biol* 39:141-160.

[71] Sutcliffe DW. 1961. Studies on salt and water balance in caddis larvae (Trichoptera): II. Osmotic and ionic regulation of body fluids in *Limnephilus stigma* Curtis and *Anabolia nervosa* Leach. *J Exp Biol* 38:521-530.

[72] Wichard W, Komnick H. 1973. Fine structure and function of the abdominal chloride epithelia in caddisfly larvae. *Z Zefforsch Mikrosk Anat* 136:579-590.

[73] Leader JP. 1972. Osmoregulation in the larva of the marine caddis fly, *Philanisus plebeius* (Walk.) (Trichoptera). *J Exp Biol* 57:821-838.

[74] Komnick H. 1977. Chloride cells and chloride epithelia of aquatic insects. *Int Rev Cytol* 49:285-328.

[75] Leader JP, Green LB. 1978. Active transport of chloride and sodium by the rectal chamber of the larvae of the dragonfly, *Uropetala carovei*. *J Insect Physiol* 24:685-692.

[76] Moens J. 1975. Ionic regulation of the haemolymph in the larvae of the dragonfly *Aeshna cyanea* (Miiller) (Odonata, Anisoptera). *Arch Int Physiol Biochim* 83:443-451.

[77] Komnick H. 1978. Osmoregulatory role and transport ATPases of the rectum of dragonfly larvae. *Odonatologica* 7:247-262.

[78] Komnick H, Achenbach U. 1979. Comparative biochemical, histochemical and autoradiographic studies of Na^+^/K^+^-ATPase in the rectum of dragonfly larvae (Odonata, Aeshnidae). *Eur J Cell Biol* 20:92-100.

[79] Frisbie MP, Dunson WA. 1988a. Sodium and water balance in larvae of the predaceous diving beetle, *Dytiscus verticalis*: An air-breather resistant to acid-induced sodium loss. *Comp Biochem Phys A* 89:409-414.

[80] Frisbie MP, Dunson WA. 1988b. The effect of food consumption on sodium and water balance in the predaceous diving beetle, *Dytiscus verticalis*. *J Comp Phys B* 158:91-98.

[81] Shaw J. 1955. Ionic regulation and water balance in the aquatic larva of *Sialis lutaria*. *J Exp Biol* 32:353-382.

[82] Zheng H, Dietz TH. 1998. Ion transport in the freshwater bivalve *Corbicula fluminea*. *Biol Bull* 194:161-169.

[83] Coimbra J, Machado J, Fernandes PL, Ferreira HG, Ferreira KG. 1988. Electrophysiology of the mantle of *Anodonta cygnea*. *J Exp Biol* 140:65-88.

[84] Dietz TH. 1978. Sodium transport in the freshwater mussel, *Carunculina texasensis* (Lea). *Am J Phys-Reg I* 235:R35-R40.

[85] Dietz TH, Graves SY. 1981. Sodium influx in isolated gills of the freshwater mussel, *Ligumia subrostrata*. *J Comp Phys B* 143:185-190.

[86] Hudson RL. 1993. Bafilomycin-sensitive acid secretion by mantle epithelium of the freshwater clam, *Unio complanatus*. *Am J Phys-Reg I* 264:R946-R951.

[87] Horohov J, Silverman H, Lynn JW, Dietz TH. 1992. Ion transport in the freshwater zebra mussel, *Dreissena polymorpha*. *Biol Bull* 183:297-303.

[88] Parks SK, Tresguerres M, Goss GG. 2007. Interactions between Na^+^ channels and Na^+^-HCO_3_^−^ cotransporters in the freshwater fish gill MR cell: a model for transepithelial Na^+^ uptake. *Am J Physiol-Cell Ph* 292:C935-C944.

[89] Hiroi J, Yasumasu S, McCormick SD, Hwang P-P, Kaneko T. 2008. Evidence for an apical Na–Cl cotransporter involved in ion uptake in a teleost fish. *J Exp Biol* 211:2584-2599.

[90] Hwang P-P, Perry SF. 2010. 8 - Ionic and acid–base regulation. In Perry SF, Ekker M, Farrell AP, Colin JB, eds, *Fish Physiology*. Vol 29. Academic Press, San Diego, CA, pp 311-344.

[91] Wang Y-F, Tseng Y-C, Yan J-J, Hiroi J, Hwang P-P. 2009. Role of SLC12A10.2, a Na-Cl cotransporter-like protein, in a Cl uptake mechanism in zebrafish (*Danio rerio*). *Am J Phys-Reg I* 296:R1650-R1660.

[92] Hwang P-P, Lee T-H, Lin L-Y. 2011. Ion regulation in fish gills: Recent progress in the cellular and molecular mechanisms. *Am J Phys-Reg I* 301:R28-R47.

[93] Lee Y-C, Yan J-J, Cruz SA, Horng J-L, Hwang P-P. 2011. Anion exchanger 1b, but not sodium-bicarbonate cotransporter 1b, plays a role in transport functions of zebrafish H^+^-ATPase-rich cells. *Am J Physiol-Cell Ph* 300:C295-C307.

[94] Inokuchi M, Hiroi J, Watanabe S, Lee KM, Kaneko T. 2008. Gene expression and morphological localization of NHE3, NCC and NKCC1a in branchial mitochondria-rich cells of Mozambique tilapia (*Oreochromis mossambicus*) acclimated to a wide range of salinities. *Comp Biochem Phys A* 151:151-158.

[95] McDonald DG, Wood CM. 1981. Branchial and renal acid and ion fluxes in the rainbow trout, *Salmo gairdneri*, at low environmental pH. *J Exp Biol* 93:101-118.

[96] Hirata T, Kaneko T, Ono T, Nakazato T, Furukawa N, Hasegawa S, Wakabayashi S, Shigekawa M, Chang M-H, Romero MF, Hirose S. 2003. Mechanism of acid adaptation of a fish living in a pH 3.5 lake. *Am J Phys-Reg I* 284:R1199-R1212.

[97] Gonzalez RJ, Wood CM, Wilson RW, Patrick ML, Bergman HL, Narahara A, Val Adalberto L. 1998. Effects of water pH and calcium concentration on ion balance in fish of the Rio Negro, Amazon. *Physiol Zool* 71:15-22.

[98] Wilson RW, Wood CM, Gonzalez RJ, Patrick ML, Bergman HL, Narahara A, Val AL. 1999. Ion and acid-base balance in three species of Amazonian fish during gradual acidification of extremely soft water. *Physiol Biochem Zool* 72:277-285.

[99] Gonzalez RJ, Preest MR. 1999. Ionoregulatory specializations for exceptional tolerance of ion‐poor, acidic waters in the neon tetra (*Paracheirodon innesi*). *Physiol Biochem Zool* 72:156-163.

[100] Gonzalez Richard J, Dalton Valerie M, Patrick Marjorie L. 1997. Ion regulation in ion‐poor acidic water by the blackskirt tetra (*Gymnocorymbus ternetzi*), a fish native to the Amazon River. *Physiol Zool* 70:428-435.

[101] Wood CM, Robertson LM, Johannsson OE, Val AL. 2014. Mechanisms of Na^+^ uptake, ammonia excretion, and their potential linkage in native Rio Negro tetras (*Paracheirodon axelrodi, Hemigrammus rhodostomus*, and *Moenkhausia diktyota*). *J Comp Phys B* 184:877-890.

[102] Gonzalez RJ, Wilson RW, Wood CM, Patrick ML, Val AL. 2002. Diverse strategies for ion regulation in fish collected from the ion‐poor, acidic Rio Negro. *Physiol Biochem Zool* 75:37-47.

[103] Freda J, McDonald DG. 1988. Physiological correlates of interspecific variation in acid tolerance in fish. *J Exp Biol* 136:243-258.

[104] Gonzalez RJ, Dunson WA. 1989. Acclimation of sodium regulation to low pH and the role of calcium in the acid-tolerant sunfish *Enneacanthus obesus*. *Physiol Zool* 62:977-992.

[105] Kumai Y, Bahubeshi A, Steele S, Perry SF. 2011. Strategies for maintaining Na^+^ balance in zebrafish (Danio rerio) during prolonged exposure to acidic water. *Comp Biochem Phys A* 160:52-62.

[106] Gonzalez-Mariscal L, Contreras RG, Bolivar JJ, Ponce A, Chavez de Ramirez B, Cereijido M. 1990. Role of calcium in tight junction formation between epithelial cells. *Am J Physiol-Cell Ph* 259:C978-C986.

[107] Stuart RO, Sun A, Panichas M, Hebert SC, Brenner BM, Nigam SK. 1994. Critical role for intracellular calcium in tight junction biogenesis. *Journal of Cellular Physiology* 159:423-433.

[108] McWilliams PG. 1982. The effects of calcium on sodium fluxes in the brown trout, *Salmo trutta*, in neutral and acid water. *J Exp Biol* 96:439-442.

[109] McDonald DG. 1983. The interaction of environmental calcium and low pH on the physiology of the rainbow trout, *Salmo gairdneri*: I. Branchial and renal net ion and H^+^ fluxes. *J Exp Biol* 102:123-140.

[110] Frain WJ. 1987. The Effect of external sodium and calcium concentrations on sodium fluxes by salt-depleted and non-depleted minnows, *Phoxinus phoxinus* (L.). *J Exp Biol* 131:417-425.

[111] Wood CM, Rogano MS. 1986. Physiological responses to acid stress in crayfish (*Orconectes*): Haemolymph ions, acid–base status, and exchanges with the environment. *Can J Fish Aquat Sci* 43:1017-1026.

[112] Jensen FB, Malte H. 1990. Acid-base and electrolyte regulation, and haemolymph gas transport in crayfish, *Astacus astacus*, exposed to soft, acid water with and without aluminium. *J Comp Phys B* 160:483-490.

[113] Potts WTW, Fryer G. 1979. The effects of pH and salt content on sodium balance in *Daphina magna* and *Acantholeberis curvirostris* (Crustacea: Cladocera). *J Comp Phys B* 129:289-294.

[114] Hollett L, Berrill M, Rowe L. 1986. Variation in major ion concentration of *Cambarus robustus* and *Orconectes rusticus* following exposure to low pH. *Can J Fish Aquat Sci* 43:2040-2044.

[115] Ellis BA, Morris S. 1995. Effects of extreme pH on the physiology of the Australian 'yabby' *Cherax destructor*: acute and chronic changes in haemolymph carbon dioxide, acid-base and ionic status. *J Exp Biol* 198:395-407.

[116] Patrick ML, Ferreira RL, Gonzalez RJ, Wood CM, Wilson RW, Bradley TJ, Val AL. 2002. Ion regulatory patterns of mosquito larvae collected from breeding sites in the Amazon rain forest. *Physiol Biochem Zool* 75:215-222.

[117] Havas M, Hutchinson TC. 1983. Effect of low pH on the chemical composition of aquatic invertebrates from tundra ponds at the Smoking Hills, N.W.T., Canada. *Can J Zool* 61:241-249.

[118] Rowe L, Berrill M, Hollett L. 1988. The influence of season and pH on mortality, molting and whole-body ion concentrations in nymphs of the mayfly *Stenonema femoratum*. *Comp Biochem Phys A* 90:405-408.

[119] Rowe L, Berrill M, Hollett L, Hall RJ. 1989. The effects of short-term laboratory pH depressions on molting, mortality and major ion concentrations in the mayflies *Stenonema femoratum* and *Leptophlebia cupida*. *Hydrobiologia* 184:89-97.

[120] Twitchen ID. 1990. The physiological bases of resistance to low pH among aquatic insect larvae. In Mason BJ, ed, *The Surface Waters Acidification Programme*. Press Syndicate of the University of Cambridge, Cambridge, U.K., pp 413-418.

[121] Lechleitner RA, Cherry DS, Cairns J, Jr., Stetler DA. 1985. Ionoregulatory and toxicological responses of stonefly nymphs (Plecoptera) to acidic and alkaline pH. *Arch Environ Contam Toxicol* 14:179-185.

[122] Whipple AV, Dunson WA. 1993. Amelioration of the toxicity of H^+^ to larval stoneflies by metals found in coal mine effluent. *Arch Environ Contam Toxicol* 24:194-200.

[123] Rockwood JP, Coler RA, Chih-Ming Y. 1988. The effect of aluminum in soft water at low pH on osmoregulation and ionic balance in the dragonfly *Libellula julia* Uhler. *Comp Biochem Physiol C* 91:499-502.

[124] Rockwood JP, Coler RA. 1991. The effect of aluminum in soft water at low pH on water balance and hemolymph ionic and acid-base regulation in the dragonfly *Libellula julia* Uhler. *Hydrobiologia* 215:243-250.

[125] Pynnönen K. 1990. Physiological responses to severe acid stress in 4 species of freshwater clams (Unionidae). *Arch Environ Contam Toxicol* 19:471-478.

[126] Bury NR, Wood CM. 1999. Mechanism of branchial apical silver uptake by rainbow trout is via the proton-coupled Na+channel. *Am J Phys-Reg I* 277:R1385-R1391.

[127] Grosell M, Wood CM. 2002. Copper uptake across rainbow trout gills: Mechanisms of apical entry. *J Exp Biol* 205:1179-1188.

[128] Goss GG, Gilmour K, Hawkings G, Brumbach JH, Huynh M, Galvez F. 2011. Mechanism of sodium uptake in PNA negative MR cells from rainbow trout, *Oncorhynchus mykiss* as revealed by silver and copper inhibition. *Comp Biochem Phys A* 159:234-241.

[129] Pyle GG, Kamunde CN, McDonald DG, Wood CM. 2003. Dietary sodium inhibits aqueous copper uptake in rainbow trout (*Oncorhynchus mykiss*). *J Exp Biol* 206:609-618.

[130] Kamunde CN, Grosell M, Lott JNA, Wood CM. 2001. Copper metabolism and gut morphology in rainbow trout (*Oncorhynchus mykiss*) during chronic sublethal dietary copper exposure. *Can J Fish Aquat Sci* 58:293-305.

[131] Galvez F, Wood CM. 2002. The mechanisms and costs of physiological and toxicological acclimation to waterborne silver in juvenile rainbow trout (*Oncorhynchus mykiss*). *J Comp Physiol B Biochem System Environ Physiol* 172:587-597.

[132] Laurén DJ, McDonald DG. 1986. Influence of water hardness, pH, and alkalinity on the mechanisms of copper toxicity in juvenile rainbow trout, *Salmo gairdneri*. *Can J Fish Aquat Sci* 43:1488-1496.

[133] Welsh PG, Skidmore JF, Spry DJ, Dixon DG, Hodson PV, Hutchinson NJ, Hickie BE. 1993. Effect of pH and dissolved organic carbon on the toxicity of copper to larval fathead minnow (*Pimephales promelas*) in natural lake waters of low alkalinity. *Can J Fish Aquat Sci* 50:1356-1362.

[134] Bianchini A, Grosell M, Gregory SM, Wood CM. 2002. Acute silver toxicity in aquatic animals is a function of sodium uptake rate. *Environ Sci Technol* 36:1763-1766.

[135] Grosell M, Gerdes RM, Brix KV. 2006. Chronic toxicity of lead to three freshwater invertebrates—*Brachionus calyciflorus, Chironomus tentans*, and *Lymnaea stagnalis*. *Environ Toxicol Chem* 25:97-104.

[136] Hemelraad J, Holwerda DA, Wijnne HJA, Zandee DI. 1990. Effects of cadmium in freshwater clams. I. Interaction with essential elements in *Anodonta cygnea*. *Arch Environ Contam Toxicol* 19:686-690.

[137] Lopes-Lima M, Freitas S, Pereira L, Gouveia E, Hinzmann M, Checa A, Machado J. 2012. Ionic regulation and shell mineralization in the bivalve *Anodonta cygnea* (swan mussel) following heavy-metal exposure. *Can J Zool* 90:267-283.

[138] Brix KV, Esbaugh AJ, Grosell M. 2011. The toxicity and physiological effects of copper on the freshwater pulmonate snail, *Lymnaea stagnalis*. *Comp Biochem Physiol C* 154:261-267.

[139] Gramigni E, Tadini-Buoninsegni F, Bartolommei G, Santini G, Chelazzi G, Moncelli MR. 2009. Inhibitory effect of Pb^2+^ on the transport cycle of the Na^+^,K^+^-ATPase. *Chem Res Toxicol* 22:1699-1704.

[140] Rogers JT, Patel M, Gilmour KM, Wood CM. 2005. Mechanisms behind Pb-induced disruption of Na^+^ and Cl^−^ balance in rainbow trout (*Oncorhynchus mykiss*). *Am J Phys-Reg I* 289:R463-R472.

[141] Gillis PL, Wood CM. 2008. The effect of extreme waterborne cadmium exposure on the internal concentrations of cadmium, calcium, and sodium in *Chrionomus riparius* larvae. *Ecotoxicol Environ Saf* 71:56-74.

[142] Craig A, Hare L, Tessier A. 1999. Experimental evidence for cadmium uptake via calcium channels in the aquatic insect *Chironomus staegeri*. *Aquat Toxicol* 44:255-262.

[143] Croghan PC, Curra RA, Lockwood APM. 1965. The electrical potential difference across the epithelium of isolated gills of the crayfish *Austropotamobius pallipes* (Lereboullet). *J Exp Biol* 42:463-474.

[144] Harvey WR. 1992. Physiology of V-ATPases. *J Exp Biol* 172:1-17.

[145] Wieczorek H. 1992. The insect V-ATPase, a plasma membrane proton pump energizing secondary active transport: molecular analysis of electrogenic potassium transport in the tobacco hornworm midgut. *J Exp Biol* 172:335-343.

[146] Boudko DY, Moroz LL, Linser PJ, Trimarchi JR, Smith PJ, Harvey WR. 2001. In situ analysis of pH gradients in mosquito larvae using non-invasive, self-referencing, pH-sensitive microelectrodes. *J Exp Biol* 204:691-699.

[147] Smith KE, VanEkeris LA, Linser PJ. 2007. Cloning and characterization of AgCA9, a novel α-carbonic anhydrase from *Anopheles gambiae* Giles sensu stricto (Diptera: Culicidae) larvae. *J Exp Biol* 210:3919-3930.

[148] O'Donnell MJ, Maddrell SHP. 1983. Paracellular and transcellular routes for water and solute movements across insect epithelia. *J Exp Biol* 106:231-253.

[149] Dietz TH, Lessard D, Silverman H, Lynn JW. 1994. Osmoregulation in *Dreissena polymorpha*: the importance of Na, Cl, K, and particularly Mg. *Biol Bull* 187:76-83.

[150] Dietz TH, Wilcox SJ, Byrne RA, Lynn JW, Silverman H. 1996. Osmotic and ionic regulation of North American zebra mussels (*Dreissena polymorpha*). *Am Zool* 36:364-372.

[151] Dietz TH, Neufeld DH, Silverman H, Wright SH. 1998. Cellular volume regulation in freshwater bivalves. *J Comp Phys B* 168:87-95.

[152] Dietz TH, Byrne RA. 1990. Potassium and rubidium uptake in freshwater bivalves. *J Exp Biol* 150:395-405.

[153] Wilcox SJ, Dietz TH. 1995. Potassium transport in the freshwater bivalve Dreissena polymorpha. *J Exp Biol* 198:861-868.

[154] Byrne RA, Dietz TH. 1997. Ion transport and acid-base balance in freshwater bivalves. *J Exp Biol* 200:457-465.

[155] Cleveland L, Little EE, Ingersoll CG, Wiedmeyer RH, Hunn JB. 1991. Sensitivity of brook trout to low pH, low and elevated aluminum concentrations during laboratory pulse exposures. *Aquat Toxicol* 19:303-317.

[156] Saito K, Nakamura N, Ito Y, Hoshijima K, Esaki M, Zhao B, Hirose S. 2010. Identification of zebrafish FXYD11a protein that is highly expressed in ion-transporting epithelium of the gill and skin and its possible role in ion homeostasis. *Front Physiol* 1:129.

[157] Craig PM, Wood CM, McClelland GB. 2007. Gill membrane remodeling with soft-water acclimation in zebrafish (*Danio rerio*). *Physiol Genomics* 30:53-60.

[158] Shahsavarani A, Perry SF. 2006. Hormonal and environmental regulation of epithelial calcium channel in gill of rainbow trout (*Oncorhynchus mykiss*). *Am J Phys-Reg I* 291:R1490-R1498.

[159] Galvez F, Wong D, Wood CM. 2006. Cadmium and calcium uptake in isolated mitochondria-rich cell populations from the gills of the freshwater rainbow trout. *Am J Phys-Reg I* 291:R170-R176.

[160] Perry SF, Shahsavarani A, Georgalis T, Bayaa M, Furimsky M, Thomas SLY. 2003. Channels, pumps, and exchangers in the gill and kidney of freshwater fishes: Their role in ionic and acid-base regulation. *J Exp Zool Part A* 300A:53-62.

[161] Bucking C, Wood CM. 2007. Gastrointestinal transport of Ca^2+^ and Mg^2+^ during the digestion of a single meal in the freshwater rainbow trout. *J Comp Phys B* 177:349-360.

[162] Baldisserotto B, Chowdhury MJ, Wood CM. 2005. Effects of dietary calcium and cadmium on cadmium accumulation, calcium and cadmium uptake from the water, and their interactions in juvenile rainbow trout. *Aquat Toxicol* 72:99-117.

[163] Baldisserotto B, Kamunde C, Matsuo AYO, Wood CM. 2004. Acute waterborne cadmium uptake in rainbow trout is reduced by dietary calcium carbonate. *Comp Biochem Physiol C Toxicol Pharmacol* 137:363-372.

[164] Baldisserotto B, Kamunde C, Matsuo AYO, Wood CM. 2004. A protective effect of dietary calcium against acute waterborne cadmium uptake in rainbow trout. *Aquat Toxicol* 67:57-73.

[165] Verbost PM, Flik G, Fenwick JC, Greco A-M, Pang PKT, Wendelaar Bonga SE. 1993. Branchial calcium uptake: Possible mechanisms of control by stanniocalcin. *Fish Physiol Biochem* 11:205-215.

[166] Barkai AI, Williams RW. 1983. The exchange of calcium in larvae of the mosquito *Aedes aegypti*. *J Exp Biol* 104:139-148.

[167] Donini A, O'Donnell MJ. 2005. Analysis of Na^+^, Cl^-^, K^+^, H^+^ and NH_4_^+^ concentration gradients adjacent to the surface of anal papillae of the mosquito *Aedes aegypti*: application of self-referencing ion-selective microelectrodes. *J Exp Biol* 208:603-610.

[168] Wigglesworth VB. 1933. The effect of salts on the anal gills of the mosquito larva. *J Exp Biol* 10:1-14.

[169] Byrne RA, Dietz TH. 2006. Ionic and acid-base consequences of exposure to increased salinity in the zebra mussel, *Dreissena polymorpha*. *Biol Bull* 211:66-75.

[170] Pietrzak JE, Bates JM, Scott RM. 1976. Constituents of unionid extrapallial fluid. II. pH and metal ion composition. *Hydrobiologia* 50:89-93.

[171] Taylor HH. 1977. The ionic and water relations of embryos of *Lymnaea stagnalis*, A feshwater pulmonate mollusc. *J Exp Biol* 69:143-172.

[172] Ebanks SC, O'Donnell MJ, Grosell M. 2010. Characterization of mechanisms for Ca^2+^ and HCO_3_^–^/CO_3_^2–^ acquisition for shell formation in embryos of the freshwater common pond snail *Lymnaea stagnalis*. *J Exp Biol* 213:4092-4098.

[173] Ahearn GA, Mandal PK, Mandal A. 2001. Biology of the 2Na^+^/1H^+^ antiporter in invertebrates. *J Exp Zool* 289:232-244.

[174] Verbost PM, Flik G, Lock RAC, Wendelaar Bonga SE. 1988. Cadmium inhibits plasma membrane calcium transport. *J Membr Biol* 102:97-104.

[175] Hogstrand C, Verbost PM, Bonga SE, Wood CM. 1996. Mechanisms of zinc uptake in gills of freshwater rainbow trout: Interplay with calcium transport. *Am J Phys-Reg I* 270:R1141-R1147.

[176] Niyogi S, Wood CM. 2004. Kinetic analyses of waterborne Ca and Cd transport and their interactions in the gills of rainbow trout (*Oncorhynchus mykiss*) and yellow perch (*Perca flavescens*), two species differing greatly in acute waterborne Cd sensitivity. *J Comp Phys B* 174:243-253.

[177] Bjerregaard P, Depledge MH. 1994. Cadmium accumulation in *Littorina littorea*, *Mytilus edulis* and *Carcinus maenas*: The influence of salinity and calcium ion concentrations. *Mar Biol* 119:385-395.

[178] Pedersen TV, Bjerregaard P. 1995. Calcium and cadmium fluxes across the gills of the shore crab, *Carcinus maenas*. *Mar Pollut Bull* 31:73-77.

[179] Bondgaard M, Bjerregaard P. 2005. Association between cadmium and calcium uptake and distribution during the moult cycle of female shore crabs, *Carcinus maenas*: An in vivo study. *Aquat Toxicol* 72:17-28.

[180] Wright DA. 1977. The effect of calcium on cadmium uptake by the shore crab *Carcinus maenas*. *J Exp Biol* 67:163-173.

[181] Clifford M, McGeer JC. 2010. Development of a biotic ligand model to predict the acute toxicity of cadmium to *Daphnia pulex*. *Aquat Toxicol* 98:1-7.

[182] Leonard EM, Pierce LM, Gillis PL, Wood CM, O’Donnell MJ. 2009. Cadmium transport by the gut and Malpighian tubules of *Chironomus riparius*. *Aquat Toxicol* 92:179-186.

[183] Buchwalter DB, Luoma SN. 2004. Differences in dissolved cadmium and zinc uptake among stream insects: Mechanistic explanations. *Environ Sci Technol* 39:498-504.

[184] Poteat MD, Buchwalter DB. 2014. Calcium uptake in aquatic insects: influences of phylogeny and metals (Cd and Zn). *J Exp Biol* 217:1180-1186.

[185] Poteat MD, Díaz-Jaramillo M, Buchwalter DB. 2012. Divalent metal (Ca, Cd, Mn, Zn) uptake and interactions in the aquatic insect *Hydropsyche sparna*. *J Exp Biol* 215:1575-1583.

[186] Markich SJ, Jeffree RA. 1994. Absorption of divalent trace metals as analogues of calcium by Australian freshwater bivalves: an explanation of how water hardness reduces metal toxicity. *Aquat Toxicol* 29:257-290.

[187] Wang Y, Evans RD. 1993. Influence of calcium concentrations on cadmium uptake by the freshwater mussel *Ellptio complanata*. *Can J Fish Aquat Sci* 50:2591-2596.

[188] Brix KV, Esbaugh AJ, Munley KM, Grosell M. 2012. Investigations into the mechanism of lead toxicity to the freshwater pulmonate snail, *Lymnaea stagnalis*. *Aquat Toxicol* 106–107:147-156.

[189] Grosell M, Brix KV. 2009. High net calcium uptake explains the hypersensitivity of the freshwater pulmonate snail, Lymnaea stagnalis, to chronic lead exposure. *Aquat Toxicol* 91:302-311.

[190] de Schamphelaere KAC, Koene JM, Heijerick DG, Janssen CR. 2008. Reduction of growth and haemolymph Ca levels in the freshwater snail *Lymnaea stagnalis* chronically exposed to cobalt. *Ecotoxicol Environ Saf* 71:65-70.

[191] Ngo HTT, Gerstmann S, Frank H. 2011. Subchronic effects of environment-like cadmium levels on the bivalve *Anodonta anatina* (Linnaeus 1758): I. Bioaccumulation, distribution and effects on calcium metabolism. *Toxicol Environ Chem* 93:1788-1801.

[192] Faubel D, Lopes-Lima M, Freitas S, Pereira L, Andrade J, Checa A, Frank H, Matsuda T, Machado J. 2008. Effects of Cd^2+^ on the calcium metabolism and shell mineralization of bivalve *Anodonta cygnea*. *Mar Freshwat Behav Physiol* 41:131-146.

[193] Lopes-Lima M, Bleher R, Forg T, Hafner M, Machado J. 2008. Studies on a PMCA-like protein in the outer mantle epithelium of *Anodonta cygnea*: insights on calcium transcellular dynamics. *J Comp Phys B* 178:17-25.

[194] Présing M, V.-Balogh K, Salánki J. 1993. Cadmium uptake and depuration in different organs of *Lymnaea stagnalis* L. and the effect of cadmium on the natural zinc level. *Arch Environ Contam Toxicol* 24:28-34.

[195] van der Velden JA, Flik G, Spanings FAT, Verburg TG, Kolar ZI, Wendelaar Bonga SE. 1992. Physiological effects of low-magnesium feeding in the common carp, *Cyprinus carpio*. *J Exp Zool* 264:237-244.

[196] van der Velden JA, Flik G, Wendelaar Bonga SE. 1992. Prolactin cell activity and ion regulation in tilapia, *Oreochromis mossambicus* (Peters): Effects of a low magnesium diet. *J Fish Biol* 40:875-885.

[197] van der Velden JA, Spanings FAT, Flik G, Zegers C, Kolar Z, Wendelaar Bonga SE. 1991. Growth rate and tissue magnesium concentration in adult freshwater tilapia, *Oreochromis mossambicus* (Peters), fed diets differing in magnesium content. *J Fish Biol* 39:83-91.

[198] Bijvelds MJC, Flik G, Kolar ZI, Wendelaar Bonga SE. 1996. Uptake, distribution and excretion of magnesium in *Oreochromis mossambicus*: Dependence on magnesium in diet and water. *Fish Physiol Biochem* 15:287-298.

[199] Shearer KD, Åsgård T. 1992. The effect of water-borne magnesium on the dietary magnesium requirement of the rainbow trout (*Oncorhynchus mykiss*). *Fish Physiol Biochem* 9:387-392.

[200] Perry SF, Vulesevic B, Grosell M, Bayaa M. 2009. Evidence that SLC26 anion transporters mediate branchial chloride uptake in adult zebrafish (*Danio rerio*). *Am J Phys-Reg I* 297:R988-R997.

[201] Gilmour KM, Perry SF. 2009. Carbonic anhydrase and acid–base regulation in fish. *J Exp Biol* 212:1647-1661.

[202] Lin T-Y, Liao B-K, Horng J-L, Yan J-J, Hsiao C-D, Hwang P-P. 2008. Carbonic anhydrase 2-like a and 15a are involved in acid-base regulation and Na^+^ uptake in zebrafish H^+^-ATPase-rich cells. *Am J Physiol-Cell Ph* 294:C1250-C1260.

[203] Tresguerres M, Katoh F, Orr E, Parks SK, Goss Greg G. 2006. Chloride uptake and base secretion in freshwater fish: A transepithelial ion-transport metabolon? *Physiol Biochem Zool* 79:981-996.

[204] Hawkings GS, Galvez F, Goss GG. 2004. Seawater acclimation causes independent alterations in Na^+^/K^+^- and H^+^-ATPase activity in isolated mitochondria-rich cell subtypes of the rainbow trout gill. *J Exp Biol* 207:905-912.

[205] Barradas C, Dunel-Erb S, Lignon J, Péqueux A. 1999. Superimposed morphofunctional study of ion regulation and respiration in single gill filaments of the crayfish *Astacus leptodactylus*. *J Crust Biol* 19:14-25.

[206] Dunel-Erb S, Barradas C, Lignon J. 1997. Morphological evidence for the existence of two distinct types of mitochondria rich cells in the gill of the crayfish *Astacus leptodactylus* Eschscholtz. *Acta Zool* 78:195-203.

[207] Barradas C, Wilson JM, Dunel-Erb S. 1999. Na^+^/K^+^-ATPase activity and immunocytochemical labeling in podobranchial filament and lamina of the freshwater crayfish *Astacus leptodactylus* Eschscholtz: evidence for the existence of sodium transport in the filaments. *Tissue Cell* 31:523-528.

[208] Stobbart RH. 1967. The effect of some anions and cations upon the fluxes and net uptake of chloride in the larva of *Aedes aegypti* (L.), and the nature of the uptake mechanisms for sodium and chloride. *J Exp Biol* 47:35-57.

[209] Scheide JI, Dietz TH. 1982. The effects of independent sodium and chloride depletion on ion balance in freshwater mussels. *Can J Zool* 60:1676-1682.

[210] Hõbe H, Wood CM, McMahon BR. 1984. Mechanisms of acid-base and ionoregulation in white suckers (*Catostomus commersoni*) in natural soft water. 1. Acute exposure to low ambient pH. *J Comp Phys B* 154:35-46.

[211] Felten V, Guerold F. 2004. Haemolymph Na^+^ and Cl^-^ loss in *Gammarus fossarum* exposed in situ to a wide range of acidic streams. *Dis Aquat Org* 61:113-121.

[212] Wilkie MP, Wood CM. 1994. The effects of extremely alkaline water (pH 9·5) on rainbow trout gill function and morphology. *J Fish Biol* 45:87-98.

[213] Wilkie MP, Laurent P, Wood CM. 1999. The physiological basis for altered Na^+^ and Cl^−^ movements across the gills of rainbow trout (*Oncorhynchus mykiss*) in alkaline (pH = 9.5) water. *Physiol Biochem Zool* 72:360-368.

[214] McGeer James C, Eddy FB. 1998. Ionic regulation and nitrogenous excretion in rainbow trout exposed to buffered and unbuffered freshwater of pH 10.5. *Physiol Zool* 71:179-190.

[215] Harris RR, Coley S. 1991. The effects of nitrite on chloride regulation in the crayfish *Pacifastacus leniusculus* Dana (Crustacea: Decapoda). *J Comp Phys B* 161:199-206.

[216] Jensen FB. 1996. Uptake, elimination and effects of nitrite and nitrate in freshwater crayfish (*Astacus astacus*). *Aquat Toxicol* 34:95-104.

[217] Stormer J, Jensen FB, Rankin JC. 1996. Uptake of nitrite, nitrate, and bromide in rainbow trout, (*Oncorhynchus mykiss*): effects on ionic balance. *Can J Fish Aquat Sci* 53:1943-1950.

[218] Claiborne JB, Heisler N. 1984. Acid-base regulation and ion transfers in the carp (*Cyprinus carpio*) during and after exposure to environmental hypercapnia. *J Exp Biol* 108:25-43.

[219] Cameron JN, Iwama GK. 1987. Compensation of progressive hypercapnia in channel catfish and blue crabs. *J Exp Biol* 133:183-197.

[220] Goss GG, Perry SF. 1993. Physiological and morphological regulation of acid–base status during hypercapnia in rainbow trout (*Oncorhynchus mykiss*). *Can J Zool* 71:1673-1680.

[221] Hyde DA, Perry SF. 1989. Differential approaches to blood acid-base regulation during exposure to prolonged hypercapnia in two freshwater teleosts: the rainbow trout (*Salmo gairdneri*) and the American eel (*Anguilla rostrata*). *Physiol Zool* 62:1164-1186.

[222] Goss GG, Perry SF. 1994. Different mechanisms of acid-base regulation in rainbow trout (*Oncorhynchus mykiss*) and American eel (*Anguilla rostrata*) during NaHCO_2_ infusion. *Physiol Zool* 67:381-406.

[223] Cameron JN, Kormanik GA. 1982. The acid-base responses of gills and kidneys to infused acid and base loads in the channel catfish, *Ictalurus punctatus*. *J Exp Biol* 99:143-160.

[224] Wheatly MG, Toop T. 1989. Physiological responses of the crayfish *Pacifastacus leniusculus* to environmental hyperoxia: II. Role of the antennal gland in acid-base and ion regulation. *J Exp Biol* 143:53-70.

[225] Wheatly MG, Toop T, Morrison RJ, Yow LC. 1991. Physiological responses of the crayfish *Pacifastacus leniusculus* (Dana) to environmental hyperoxia. III. Intracellular acid-base balance. *Physiol Zool* 64:323-343.

[226] Henry RP, Saintsing DG. 1983. Carbonic anhydrase activity and ion regulation in three species of osmoregulating bivalve molluscs. *Physiol Zool* 56:274-280.

[227] Ngo HTT, Gerstmann S, Frank H. 2011. Subchronic effects of environment-like cadmium levels on the bivalve *Anodonta anatina* (Linnaeus 1758): III. Effects on carbonic anhydrase activity in relation to calcium metabolism. *Toxicol Environ Chem* 93:1815-1825.

[228] Istin M, Girard JP. 1970. Dynamic state of calcium reserves in freshwater clam mantle. *Calc Tiss Res* 5:196-205.

[229] Larsen EH, Simonsen K. 1988. Sulfate transport in toad skin: Evidence for mitochondria-rich cell pathways in common with halide ions. *Comp Biochem Phys A* 90:709-714.

[230] Dietz TH, Byrne RA. 1999. Measurement of sulfate uptake and loss in the freshwater bivalve *Dreissena polymorpha* using a semi-microassay. *Can J Zool* 77:331-336.
